# “*Tazomoka* Is Not a Problem”. Local Perspectives on Malaria, Fever Case Management and Bed Net Use in Madagascar

**DOI:** 10.1371/journal.pone.0151068

**Published:** 2016-03-04

**Authors:** Chiarella Mattern, Dolorès Pourette, Emma Raboanary, Thomas Kesteman, Patrice Piola, Milijaona Randrianarivelojosia, Christophe Rogier

**Affiliations:** 1 Epidemiology Unit, Institut Pasteur de Madagascar, Antananarivo, Madagascar; 2 Centre Population et Développement (CEPED), Institut de Recherche pour le Développement, Paris, France; 3 Université Catholique de Madagascar, Antananarivo, Madagascar; 4 Malaria research Unit, Institut Pasteur de Madagascar, Antananarivo, Madagascar; 5 Institut Pasteur de Madagascar, Antananarivo, Madagascar; Centre for Geographic Medicine Research Coast, KENYA

## Abstract

**Background:**

Although its incidence has been decreasing during the last decade, malaria is still a major public health issue in Madagascar. The use of Long Lasting Insecticidal Nets (LLIN) remains a key malaria control intervention strategy in Madagascar, however, it encounters some obstacles. The present study aimed to explore the local terminology related to malaria, information channels about malaria, attitude towards bed nets, and health care seeking practices in case of fever. This article presents novel qualitative findings about malaria. Until now, no such data has been published for Madagascar.

**Methods:**

A comparative qualitative study was carried out at four sites in Madagascar, each differing by malaria epidemiology and socio-cultural background of the populations. Seventy-one semi-structured interviews were conducted with biomedical and traditional caregivers, and members of the local population. In addition, observations of the living conditions and the uses of bed net were conducted.

**Results:**

Due to the differences between local and biomedical perspectives on malaria, official messages did not have the expected impact on population in terms of prevention and care seeking behaviors. Rather, most information retained about malaria was spread through informal information circulation channels. Most interviewees perceived malaria as a disease that is simple to treat. Tazomoka (“mosquito fever”), the Malagasy biomedical word for malaria, was not used by populations. Tazo (“fever”) and *tazomahery* (“strong fever”) were the terms more commonly used by members of the local population to refer to malaria related symptoms. According to local perceptions in all areas, tazo and tazomahery were not caused by mosquitos. Each of these symptoms required specific health recourse. The usual fever management strategies consisted of self-medication or recourse to traditional and biomedical caregivers. Usage of bed nets was intermittent and was not directly linked to protection against malaria in the eyes of most Malagasy people.

**Conclusions:**

This article highlights the conflicting understanding of malaria between local perceptions and the biomedical establishment in Madagascar. Local perceptions of malaria present a holistic vision of the disease that includes various social and cultural dimensions, rather than reflecting one universal understanding, as in the biomedical image. The consideration of this “holistic vision” and other socio-cultural aspects surrounding the understanding of malaria is essential in implementing successful control intervention strategies.

## Introduction

For several decades, Madagascar has been characterized by recurrent political crises [1]. This instability has led to a weakening of the population and health system in many ways [2]. In this context, malaria remains a major public health issue in Madagascar. At the same time, intensive control interventions have led to a decrease of the malaria burden during the last decade in most African endemic countries [3]. Until 2007, malaria was the most common cause of hospital mortality and the second cause of morbidity in Malagasy children below 5. In 2011, it was the 10^th^ most common cause of hospital mortality and the 4^th^ cause of morbidity in children below 5 in Madagascar [4]. Despite this progress, outbreaks occurred in various locations throughout the island in 2012 [5]. The country’s epidemiological profile is unique: there are five different epidemiological patterns of malaria depending on (i) the seasonality of transmission, (ii) the duration of the transmission season, and (iii) whether it is a region prone to outbreaks or not. The National Malaria Control Program currently implements the following interventions: Intermittent Preventive Treatment (IPT) during pregnancy, Long Lasting Insecticidal Nets (LLIN) distributions, Indoor Residual Spraying (IRS) campaigns, fever case management with systematic diagnostic by Rapid Diagnostic Tests (RDT) or microscopy, and treatment of non-complicated malaria cases by Artemisinin-based Combination Therapy (ACT). Anti-malaria treatment is available for free or at low price at the various steps of the health pyramid down to the community level where community health workers are trained to test and deliver treatment to children under 5 years of age. However, the most recent Malaria Indicator Survey [6] results show that less than 20% of febrile children receive artemisinin-based combination therapy (ACT).

Despite the evidence that the use of LLINs prevents malaria-related morbidity and mortality, the use of LLINs encounters some obstacles [7, 8]. Several qualitative studies have shown the underlying factors for the use of bed nets as prevention modality against malaria: access to information [9], discomfort [10, 11], design of bed nets and practical and technical difficulties related to the installation of the net above the mat [12], costs of bed nets and economic resources of households [13, 14], education [15–17], local illness perceptions [8, 18] organizational obstacles of the health system (lack of staff at clinics, insufficient number of LLINs distributed) [19], nights away from home [20, 21], and perception of mosquito density [8]. The first aim of this study is to explore these issues in Madagascar.

This literature shows that health seeking behavior is influenced by perceptions of the seriousness of a disease, among other factors [22–25] such as the cost of treatment, knowledge of its cause and ability to diagnose and treat, and episodes of diseases previously experienced by peers. In case of simple fever or uncomplicated malaria, fast, close, and cheap solutions are preferred [23, 26, 27]; self-medication based on industrial pharmaceutical drugs or traditional remedies. When the disease takes a serious nature, (e.g. loss of appetite, poor general condition, seizures, coma) there is a "special appeal" [26, 27] to health care practitioner or traditional healer. The choice to use one option or the other is not exclusive and can be done simultaneously. The international literature also emphasizes the role of collectivity in decision making and health care management [28]. Although qualitative studies on malaria are abundant on these issues on the international level, (e.g. perceptions on malaria, health seeking behavior), the information in this research field are rare in Madagascar. The second aim of this study is to address this knowledge-gap by exploring these issues in Madagascar.

The final aim of this study was to examine the socio-cultural factors associated with the use of malaria control interventions. We analyzed the social representation of malaria and the malaria prevention modalities used by populations in four endemic areas of Madagascar. Specifically, this article intended to analyze malaria related perceptions and fever case management, and the bed net practices resulting from these perceptions. The data presented here concerns the qualitative component of a nationwide survey on malaria conducted in 2012–2013 in Madagascar, led by the Epidemiological and Malaria Unit of the Institut Pasteur de Madagascar. This project was aimed at measuring the coverage and effectiveness of malaria control interventions in terms of infection, morbidity and mortality, and the factors associated with their effectiveness. This was measured by both qualitative and quantitative approaches. The project was named MEDALI (Mission d’Etude des Déterminants de l’Accès aux Méthodes de Lutte antipaludique et de leur Impact) [29] (S1 File). The qualitative component was supervised by D.P. Investigative tools and analyses grid were elaborated by D.P., C.M and E.R. All data were collected by the same team [30].

## Materials and Methods

### Study setting

Madagascar is a huge island and extends over 500 km from east to west and 1500 km from north to south. The relief divides the island into three bands: a narrow coastal strip in the east, the Highlands in the center, and an area of low plains and plateaus in the West. The country is characterized by a large diversity of climatic variations: the east of the country may suffer from floods at the same time that the South encounters long periods of drought.

Madagascar's diversity is also reflected in the origin of the Malagasy population, which remains an ethnological enigma. The contribution of South East Asia and Central and Eastern Africa is visible in the social organization, language, and religion. All the peoples of Madagascar share a culture that represents a synthesis of these two inputs, one of which may predominate by region [31, 32]. This diversity of origins, climates, and landscapes, induces a mosaic of populations and practices that are difficult to represent in a single study. So, to capture practices as diverse as possible, we chose four different geographical areas among the 31 zones of the MEDALDI project: an area in the East (Mananjary), one in the Fringe (Moramanga), one in the Highlands (Fianarantsoa) and one isolated rural area, located in the north of the island (Antsohihy). The selection of these qualitative investigation areas was based on epidemiological criteria, social and cultural features of ethnic groups and the vector control interventions implemented in these sites: LLIN (Moramanga, Mananjary, Antsohihy) and IRS (Moramanga and Fianarantsoa) (S1 Fig).

In terms of malaria transmission, the selected regions have different epidemiological profiles. The region of Mananjary in the East of the island is characterized by high and permanent transmission; populations are exposed to vectors on a daily frequency throughout the year. The region of Moramanga is located on the fringe between the coast and highlands, and is characterized by a low and seasonal transmission. The region of Fianarantsoa is located on the central highlands where the transmission is almost nul. This area is characterized by an economic immigration that facilitates importation of malaria. The region of Antsohihy in the North is characterized by moderate and seasonal transmission, and variable presence of mosquitoes. In February 2012 an outbreak, which was attributed to malaria after epidemiological and parasitological investigations, caused many deaths in this area. In all areas the main activity is rice cultivation. The difference between the regions lies in their level of wealth as indicated by differences in housing materials and household’s belongings. The northern area (Antsohihy) is characterized by cash crops for export (cocoa, vanilla) which tends to make it richer than other areas. Lack of social and health infrastructures, roads, and markets represents a significant obstacle to development in the all the areas investigated.

Data was collected between May and December 2012. The study was designed in two steps: in August 2012 an exploratory mission was conducted in order to identify the *fokontany* of investigation- the smallest Malagasy administrative entity—and October and December 2012 in-depth missions between were carried out.

### Data collection and analysis

An anthropological approach was taken to data collection, the following qualitative tools were used: (i) semi-structured interviews of various categories of individuals (community members, health workers, traditional healers and traditional birth attendants), (ii) direct observations (living conditions, installation and use of bed nets, environment, water supply point, hygiene, queue for consultation at the health center, availability of health workers) and (iii) informal interviews in the context of a annual mass distribution of LLINs. Observations allowed a comparison between practices and discourses about usage of bed nets.

In order to standardize interviews, we developed interview templates. A total of 71 in-depth interviews were carried out with two categories of participants: community members (men and women) and healthcare providers. More specifically, 54 interviews with community members (26 men and 28 women) and 17 with health workers (biomedical health workers–nurses, doctors, community health agents–and traditional healers and birth attendants) were carried out. For the identification of participants amongst community, purposive sampling was used to ensure gender and age ratio (between 20 and 80 years of age). One out of two houses was invited to participate, until we reached our objectives in terms of gender and age ratios and data saturation. A minimum quota of 14 interviews per area was determined.

All persons solicited for the study agreed to participate. The interview template for villagers focused on fever and malaria (vernacular words used to refer to malaria, perceptions, causes, symptoms and steps of the care seeking), malaria prevention practices, information channels about malaria and knowledge of prevention messages. The health worker’s template focused on treatment of malaria, time spent for providing care, difficulties met in providing care, information channels about malaria and involvement in sensitization and prevention actions. We interviewed villagers at home, and professionals at their work place. The interviews were carried out in the Malagasy language with an interpreter. The National Ethics Committee under the Ministry of Health of Madagascar gave us ethical clearance for this study. We obtained verbal consent from participants before starting or recording interviewees, written consent was not required by the Committee for qualitative studies. Additionally, in order to receive authorization to work in these areas, and in respect of the health and administrative hierarchy in place in Madagascar, courtesy visits were made to the administrative authorities (mayors) and sanitary authorities (medical inspectors) at Regional Health Centers. On the local level, the *fokontany* presidents informed the community of our presence during village meetings. Along with these efforts, we also presented ourselves to traditional leaders to facilitate access to households.

The number of interviews was determined by the data saturation criterion and the time limit for each mission on the field. The objective was to include the same number of men as women, divided according to different age groups (under 20 years, 20–30 years old, 30–40 years old and more than 40 years old). Our choice of interlocutors was dependent upon availability at the time of our investigation. One in two eligible households was interviewed. In all areas, all doctors, nurses, traditional healers were interviewed.

All the interviews were fully transcribed and translated in French by a team of five bilingual translators. We analyzed the interviews according to a thematic analysis chart that was designed to identify the themes cited in each interview and to make comparisons in order to highlight differences and recurrences in the discourses.

## Results

Results presented here concern communication issues on malaria, perceptions of malaria, health seeking behavior in case of fever, and use of mosquito nets.

### Information channels about malaria

The Malagasy word for malaria is *tazomoka* which literally means "mosquito fever". *Tazomoka* is the word used by the biomedical establishment for malaria. However, this term was almost never used by interviewees, and when it was used, it was onlyafter a diagnosis had been done by biomedical health workers.

Several channels disseminate information on tazomoka: radio, posters, mass awareness campaigns during village meetings and consultations with health professionals. Interviewees say they appreciate the information transmitted about *tazomoka* on the radio. However, such information is sometimes misunderstood. For example, one man said he heard on the radio that sickle cell anemia is a new disease transmissible through mosquitos. A woman claims to have heard that *tazomoka* is a "problem in Tana".

*«* Tazomoka *is a problem especially in Tana*, *according to what we hear on the radio*. *Here it is not a problem» [Woman*, *60 years old]*.

Village meetings were places of socialization and opportunities to exchange with their neighbors. Most interviewees were not able to recall all of the information given during these meetings. For example, in all areas they retained a specific message "you have to sleep under a mosquito net" but did not retain information on maintenance of the nets or duration of treatment effectiveness. At the time of the outbreak in Antsohihy, official messages seemed not to have had the expected impact on population: for instance, one of the messages transmitted by the health staff consisted in saying that the vectors of *tazomoka* were only female mosquitoes, which could be identified by their long legs.

*During the epidemic*, *a message was sent by health staff*: *it is only the female mosquitoes that give malaria*. *But patients asked me "How to recognize female mosquito*?*" I told them that it was the one with the long legs [Health Staff*, *40 years old]*.

National organizations and various NGOs disseminate many messages related to health (e.g. family planning, prevention against diarrhea, tuberculosis). These messages were sometimes competing, and confused the information that was received. As a result, for instance, some interviewees came to believe that the cause of the malaria outbreak in Antsohihy was related to a hygiene issue and they cleaned their hands as often as possible to protect themselves.

According to both caregivers and patients, given the lack of time available for consultation with biomedical health professionals, only minimal information was transmitted at this occasion.

*When we go for a consultation to the doctor*, *he gives no explanation of the cause or name of the disease*. *He just gives the treatment*. *The doctor asks the patient's symptoms without even touching him [Female*, *26 years old]*.

When the question was asked, interviewees couldn’t tell the name of the drug delivered at the health center during consultation for malaria related symptoms. Likewise, most women didn’t know the names and the purpose of the drugs received during pre-natal consultation (including ITPs). On this particular issue, many caregivers said they did not disclose the name of ITP to pregnant women in order to avoid self-medication. Biomedical health professionals acknowledged that during consultation they only gave the information on the drug dosage. Limited time available for consultation was an explanation to this lack of communication. Another explanation advanced was that in the countryside, the patient is seen as illiterate, and not able to understand such details. This perception was largely shared among health staff. At the same time, we observed a lack of interest of patients for the biomedical names of diseases and details about treatments prescribed. Interviewees reported that they stop treatment when they feel better, without taking into account the treatment period prescribed by the caregiver. Consultation is not perceived as a place where questions can be asked.

*During the consultation*, *we do not usually ask the doctor where the disease comes from*. *I didn’t study long at school so I'm not used to do analysis on the origin of the disease*. *I believe in what the doctor says because he has studied health [Man*, *65 years old]*.

We observed that most information retained about malaria was spread through informal information circulation channels such as rumors, by word of mouth or discussions between closest friends/relatives.

### Local perspectives on malaria

The symptoms related to malaria (fever, seizures) are not interpreted by the population as necessarily being linked to *tazomoka*, (i.e. the infectious disease that occurs due to the bite of a mosquito). Two popular nosological entities were frequently used by interviewees to refer to malaria: *Tazo* (“fever”) which referred to the sensation of warm body and simple fever and *tazomahery* (“strong fever”), when high fever was accompanied by seizures. *Tazo* in everyday language means “fever” and may be accompanied by more symptoms defined as frequent and benign: flu, cough, and diarrhea. The symptoms of *tazomahery* are similar to the biomedical category of severe malaria.

*Tazo* was perceived as simple to treat through self-medication, the habitual care seeking behavior in case of illness. For the biomedical health professionals, the difficulty in the differential diagnosis of malaria, coupled with frequent shortages of Rapid Diagnostic Tests (RDT), has been frequently cited by caregivers as a problem in the management of the disease.

*If there are no RDT because of a stock shortage*, *which happens two or three times a year*, *you can confuse the symptoms*. *Sometimes typhoid fever might be confused with malaria*. *That's why shortages of RDT are problematic because then one must treat the patient for both diseases at once*. *This includes an increase in the cost of therapy [Health Staff*, *age unknown]*.

According to local perceptions in all areas, *tazo* or *tazomahery* were not caused by mosquito. *Tazo* was mainly caused by an imbalance between the individual and the environment, such as a weather change. In Antsohihy, *tazomahery* was perceived as a supernatural disease brought about by ancestors’ as a sanction due to the violation of an ancestral taboo (e.g. bathing in a sacred lake). In Mananjary, it was mainly interpreted within a social dimension through the power of witchcraft. In local perceptions, from the time when convulsions appear, the disease was considered as serious and worrying. But these symptoms were not interpreted as belonging to *tazomoka*.

The outbreak attributed to malaria by health authorities in February of 2012 marked the memory of Antsohihy’s inhabitants. A health worker in Antsohihy declared that during the rainy season of 2012, 70% of consultations were related to malaria. The villagers gave various reasons to the outbreak, but only few people recognized *tazomoka* to be the cause, because of four main reasons. The first reason was the low density of mosquitoes observed during the outbreak. The second was that so far, mosquitoes and *tazomoka* had never been fatal in the view of the respondents. In certain circumstances before the outbreak, villagers slept outside of their houses without mosquito nets, e.g. when the cattle farmers annually migrated to seek water and pasture land for the herds of zebus, or when they harvested rice. According to our speakers, in spite of lack of protection, these age-old practices had never brought about death. The third reason for which *tazomoka* was not incriminated was that during the outbreak, the usual *tazo* management practices, i.e. self-medication, did not give the expected results, reduction of fever and recovery. The last reason that confused the population was that the symptoms during the outbreak (diarrhea, vomiting, rapid loss of consciousness after the first signs of fever) were different from the usual symptoms of *tazo*. For all these reasons, for the villagers, the cause of the outbreak couldn’t be *tazomoka*.

*I think it can’t be* tazo *because that disease has never killed*. *Then I don’t understand why this year it would be stronger than before*. *Before it could be cured with Paracetamol*®. *But this year it gave such a bad headache; it’s another reason to say that it was not* tazomoka *[Man*, *70 years old]*.

The populations looked for other causes of the disease. Since mainly children suffered and died from the outbreak, some blamed the last tetanus vaccination campaign, which had taken place a few weeks before and targeted children. Others, who had received various sensitization messages in the previous months, identified dirt and lack of hygiene as causes of the outbreak. Some interviewees, puzzled by the immediate consequences of the disease, gave a supernatural explanation. The outbreak was then interpreted as a sign coming from “up above” of the ancestors’ anger because of the lack of respect for a sacred place.

In agreement with the numerous interpretations of the origins of the outbreak, many prevention attitudes came up: refusal to participate to the vaccination campaigns that followed the outbreak, increased caution as to hygiene and food eaten, construction of latrines, wearing amulets and traditional objects of protection. For the minority of individuals who had assimilated the association between outbreak and mosquito, more regular use of bed nets.

*In my view*, *after the outbreak there was no change in behavior with the bed nets use for people who do not believe that it is the mosquito that gives* tazomoka. *They continue to discuss out in the full moon even if it's evening*. *But for those who have been persuaded by the message*, *a behavioral change can be observed [Man*, *40 years old]*.

### Care seeking behavior in case of fever

In all sites, most people affected by fever declared resorting to self-medication based on plants and pharmaceuticals. Most individuals bought drugs from the informal sector: grocers, street vendors, individuals who have drugs for sale at home, or itinerant hawkers.

*When the child begins to have warm skin*, *the mother will buy Quinine*, *Paracetamol*® *at the teacher who has drugs at her home*. *The next morning*, *sometimes the fever has dropped*. *But if not*, *if the mother sees the child does not eat*, *does not play*, *she will talk to his neighbors who will advise her to take him to the doctor*. *But it is often when the child begins to have seizures that their parents bring him to the doctor [Woman*, *52 years old]*.

*The usual practice when a person is sick is to buy drugs found at the local grocery store*. *Either the person does not want to go to the hospital because it is too far*, *or he has no money*. *Then*, *he waits until the disease gets worse before going to the hospital*. *So it becomes difficult to treat the disease [Man*, *38 years old]*.

Self-medication practices were also observed in villages where there was a public health center and its drugstore (Fianarantsoa and Antsohihy). In all study sites, reasons mentioned for using the informal sector included convenience (availability of drugs day and night, social proximity of the vendors), geographical and economic affordability (retail at lower costs and/or on credit, availability of drugs within the isolated villages).

When resorting to these retailers, the process was always the same: either the patient described his symptoms and the seller offered drug(s) that he thought adequate, or the patient had already made his choice based on a previous pathological experience. It was observed in all areas that the close circle (neighbors, relatives) and word of mouth played an essential role in the choice of drugs and in care seeking practices. Previous episodes of fever, for which a doctor's visit had been made, guided the choice of drug when new episode of fever appeared. One would then rely on an old prescription to buy drugs.

*It's the gossip of the neighborhood that determines which medication you have to take for self-medication*. *For example*, *if your neighbor brought his child to the doctor and he was cured*, *he will tell his neighbors that these drugs worked on the child*, *so others will do the same if the same symptoms appear [Caregiver*, *30 years old]*.

Only when the patient’s health got worse, or when the available community resources were exhausted, did he resort to a health facility. As a consequence, he would get a medical diagnosis and received proper treatment only at the end of the care seeking process. In the case of malaria, we observed that there were about three days between the moment fever appeared and the consultation at a health unit.

People declared that, once they arrived at the health center, there was a long queue before getting to the consultation, and that health staff only spent a few minutes on consultation. Some patients had to pay for the ACT treatment, as not all interviewees were informed of the gratuity of the ACT treatment.

Along with self-medication based on industrial pharmaceutical drugs, the use of traditional herbal remedies was also mentioned in fever case management, especially when the cause of fever was believed to be caused by a magic spell.

*I prefer to take at the same time drugs prescribed by doctors and go to traditional healers because modern medicine cannot cure what traditional can heal*, *and vice versa [Man*, *64 years old]*.

### Reported prevention modalities about bed nets

In all areas, the most frequent reason for bed nets use was protection from inconvenience caused by insects, e.g. stings from fleas and mosquitoes or noise nuisance from mosquitoes.

*I use the net because I don’t like when little insects fall on the bed*, *insects can enter into children’s ears*. *Another reason why I use bed nets is because it protects against the cold*. *Because children are too young to think about covering themselves with bed sheets and it’s this way they catch fever [Woman*, *50 years old]*.

Beyond protection against insects, other factors promoting use of bed nets were mentioned such as protection of the intimacy of the parents. Bed net usage was reported to increase when mosquitoes were present in the environment. According to the interviewees, Mananjary was an area where mosquitoes were present in swarms. In that area, the bed net was part of families’ daily lives, and in the rainy season, when mosquitoes were more numerous, the speakers were more careful in the use of it. The bed net was part of the birth and wedding kit; it was part of the equipment the couple must have to live together. According to interviews with men, it was perceived more as the sign of a man’s capacity to support a family than a means of protection. In the three other areas, due to the perceived sparse density of mosquitoes during the year, bed nets were used to protect against the cold in the winter, or as decoration, as it was seen in Antsohihy. With its colored ribbons and its openings on the four sides, the “four doors bed net” was quite popular in Antsohihy. These bed nets cost between 20 000 and 100 000 ariary (approx. 7.00–35.00 euros), on the contrary, LLINs were free of charge during distribution campaigns and 3 000–4 000 ariary (approx. 1.00–1.30 euros) in shops and drugstores. The richer families that could choose between a free LLINs or a “four doors bed net” would buy the latter, or would make a “four door bed net” with several LLINs. Poorer families would use free LLINs and, in case of shortage, they would not use their resources to buy additional mosquito nets to accommodate all family members.

In all study sites, the prioritization in the use of nets was largely influenced by messages that emphasized on the vulnerability of the target beneficiaries—pregnant and lactating women, and children less than 5 years- leading to the idea that other family members would be less exposed to *tazomoka*. In two out of four study sites, men said they were not at risk to get *tazomoka*.

*I do not use a net*, *because I do not have children*. *I'm a man*, *I do not often get sick [Man*, *24 years old]*.

Right after these vulnerable groups, parents were prioritized in the use of nets, since it preserved the couple’s privacy. In three out of four study sites, it was mentioned that children of the same gender over ten years of age could not share a same bed because of traditional taboos (*fady*).

*We only have one net and it’s me who’s sleeping under it with my six years old child and my husband because as I’m pregnant*, *so I’m more likely to catch tazomoka*. *My two other children (7 and 9 years old) doesn’t sleep under nets [Woman*, *27]*.

*As we don’t have toddlers at home anymore*, *we only use nets when there are mosquitos because tazomoka only affects children [Men*, *48 years old]*.

Some reasons mentioned for the non-use of bed nets were common in all study sites: the feeling of suffocation especially with high temperature even during the night, irritation of the skin in contact with the net, loss of light in the room, and difficulties related to the fixing of the nets in tight spaces. Several local prevention measures to avoid having mosquitoes into the houses were cited: insecticide coils, burning leaves (cypress, orange or mango), or diverting smoke from charcoal and wood cook stoves into houses.

Observations were made of alternative usage of nets: fishing or crop protection. But observations implicated that such nets were already patched and stitched together and could not be used as protection anymore. In case of distribution of new bed nets, the pervious nets were used until they were useless. This was especially true if the new nets distributed were not appreciated because of the color, type of net veil, or type of mesh.

*I have not yet installed the new net; it is still stored in its packaging with clothing*. *I do not like it because it has large meshes; I still use the old one because they are smaller*. *In addition*, *the product on the new net gives the cold*. *The fabric is stiff and uncomfortable*, *my hair hang in there [Woman*, *26 years old]*.

## Discussion

While from an epidemiological perspective, malaria is a critical health issue in Madagascar, in local perspectives, the general population does not consider *tazomoka* as a key public health problem. In local perspectives, malaria related symptoms were categorized into two types of popular nosological entities [33]: *tazo* and *tazomahery*. Both of these nosological entities are associated with specific causes. For the interviewees, only *tazomahery* requires use of specialized care other than the habitual self-medication: consultation and drugs prescribed by a biomedical health worker. A popular definition of malaria as a simple fever has already been noted in other studies [23, 34–36]. As our results demonstrate, the messages surrounding malaria affects the way in which it is understood [37].

Traditional beliefs and biomedical explanations are not mutually exclusive one of the other, e.g. interviewees reported resorting both to the biomedical sphere, through self-medication with pharmaceutical drugs, and to the traditional sphere, attributing the cause of the symptoms to a violation of an ancestral taboo, simultaneously.

In all areas studied, the plurality of prevention messages has led to confusion between diseases, and more importantly for public health initiatives, between their cause and prevention modalities. In the case of the malaria outbreak in Antsohihy, rumors demonstrated a significant distrust in the biomedical interpretation of the disease. Messages such as those promoting bed nets’ protective powers for the most vulnerable targets, i.e. pregnant women and children less than 5 years of age, resulted in less careful prevention practices among children between 5 and 15 years. As a consequences, children of this age category, as well as men, were often sleeping without protection from bed nets, however both groups share a similar risk in terms of infection and malaria-associated morbidity [38, 39]. Moreover, the way these malaria messages were interpreted introduced a gender dimension in the perception of malaria.

The results of this study also highlight unequal power distribution in the doctor-patient relationship, which also has been shown in other contexts [39]. Medical consultation is not designed as a space for exchange of information. It is rather a meeting between the representatives of two "cultures", a professional culture and popular culture, each with its own language and understanding of disease.

At this point, several recommendations can be made to improve the situation. First, concerning the content of awareness campaigns; until now, popular nosological entities used by the general population to refer to malaria, *tazo* and *tazomahery*, have never been included in malaria eradication campaigns. We recommend to re-imagine malaria and to re-cast malaria back into broader terms that takes better account of these nosological entities [37]. Along the same line, we recommend dropping all messages that do not concern prevention and fever case management, such as the identification of female Anopheles as the vector. "Mass" diffusion of the information (radio, posters, and general meetings) should be replaced by methods based on interpersonal contacts. In parallel, given that usage of bed nets is intermittent and not directly linked to protection against malaria, the mosquito net promotion messages might rely more on the benefits highlighted by the general population: e.g. protection against insects, comfort, and preservation of intimacy. We also recommend avoiding standardized messages. Promotion messages for bed nets should be adapted to local specificities and social realities [40]. For example, messages should not be the same in the areas where the use of the net is already a local habit as compared to areas where there is strong resistance to the use of the net.

Concerning bed net distribution guidelines, it may be necessary to distribute more nets so that all family members can sleep under a net, especially in areas where it is taboo for opposite-sex children to sleep side by side. Finally, we think that the improvement of the relationship between care providers and patients is a key factor and should be considered as a priority for the Ministry of health in Madagascar. A majority of respondents deplore the lack of listening and empathy on behalf of health professionals. This could be explained by the social distance between these categories of actors [41]. This new approach would strengthen public confidence towards the health care system, and improve communication between caregivers and population about disease causes, symptoms, and treatment. To do this, health professionals should be trained in the social and psychological aspects of the doctor-patient relationship.

### Methodological limitations

With the aim of understanding the barriers to adherence to malaria control strategies, we mainly analyzed local perspectives, through the concepts of risk perception and gravity of malaria. One limitation of this study lies in the overly specific focus on malaria without a more local understanding of the body, health, and well-being.

## Conclusion

The present article highlights tensions between the local and biomedical perceptions of malaria in all registers explored in this study: care seeking behaviors, disease explanations, understanding of prevention messages, doctor-patient relationship, and prevention behaviors. During sensitization campaigns and consultations, malaria control actors and biomedical health staff use a jargon specific to the biomedical perspective of malaria. Our findings demonstrate that this approach is not efficient in terms of popular adherence to the control strategies, particularly concerning bed nets use and fever case management. Local perspectives on malaria present a holistic vision of malaria that includes multiple social and cultural dimensions, rather than reflecting one universal vision, as in the biomedical image of the disease. The consideration of these socio-cultural aspects in malaria control intervention strategies is essential to a better popular adhesion to malaria control strategies.

## Supporting Information

S1 Fig Map of the investigative areas. Christophe Rogier, Monitoring committee (MEDALI), Institut Pasteur de Madagascar 2013.
(DOCX)

S1 File Rapport final de l’étude qualitative (Medali). Pourette D, Mattern C, Raboanary E: Antananarivo; 2013.
(PDF)
