# Supplementary material for: “Tazomoka Is Not a Problem”. Local Perspectives on Malaria, Fever Case Management and Bed Net Use in Madagascar
Source: PLoS One. 2016 Mar 4;11(3):e0151068. doi: 10.1371/journal.pone.0151068 (PMC4778873; doi:10.1371/journal.pone.0151068)
Supplement: S1 Fig — Christophe Rogier, Monitoring committee (MEDALI), Institut Pasteur de Madagascar 2013. (DOCX) [file pone.0151068.s001.docx]

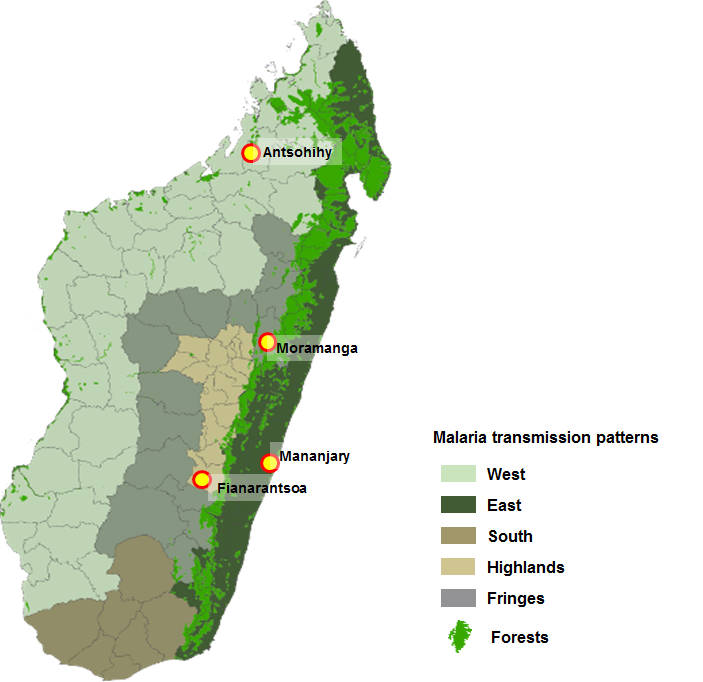


**Figure 1 : Investigative areas**

S1 Figure1: Map of the investigation areas, Christophe Rogier, Monitoring committee (MEDALI), Institut Pasteur de Madagascar 2013.
