# Supplementary material for: “Tazomoka Is Not a Problem”. Local Perspectives on Malaria, Fever Case Management and Bed Net Use in Madagascar
Source: PLoS One. 2016 Mar 4;11(3):e0151068. doi: 10.1371/journal.pone.0151068 (PMC4778873; doi:10.1371/journal.pone.0151068)
Supplement: S1 File — Pourette D, Mattern C, Raboanary E: Antananarivo; 2013. (PDF) [file pone.0151068.s002.pdf]

RAPPORT DE MISSION

Mission n° 12INI110

Dates de la période considérée : du 14/05/2012 au 18/02/2013

Madagascar

« Mission d'étude d'impact des interventions du Fonds mondial »

PROJET MEDALI (Mission d'Etudes des Déterminants de l'Accès aux méthodes de Lutte antipaludique et de leur Impact) - VOLET QUALITATIF

Dr Dolorès POURETTE (Institut de Recherche pour le Développement, CEPED UMR 196)
Chiarella MATTERN (Institut Pasteur de Madagascar)
Emma RABOANARY (Institut Pasteur de Madagascar)

Mission financée par l'Initiative 5%. L'Initiative 5% est financée par le Ministère des Affaires Etrangères et européennes (MAEE) en contribution indirecte au Fonds mondial de lutte contre le sida, la tuberculose et le paludisme (FM), et mise en œuvre par France Expertise Internationale (FEI).

Le présent compte-rendu ne reflète pas nécessairement les points de vue et opinions du MAEE, du FM ou de FEI.

INITIATIVE 5%
SIDA, TUBERCULOSE, PALUDISME

Le Fonds mondial
De lutte contre le sida, la tuberculose et le paludisme

FEI
France Expertise Internationale

Liberté • Égalité • Fraternité
RÉPUBLIQUE FRANÇAISE
MINISTÈRE DES AFFAIRES ÉTRANGÈRES ET EUROPÉENNES

Institut Pasteur de Madagascar

IRD
Institut de recherche pour le développement

CePeD
Centre Population & Développement

UCM

EQUIPE D'INVESTIGATION MEDALI

- **Coordinateur :**
  Mr Thomas KESTEMAN (Unité de Recherche sur le Paludisme, Institut Pasteur de Madagascar)

- **Co-investigateurs :**
  - Dr Jemima MAHATOVO (IPM)
  - Pr Milijaona RANDRIANARIVELOJOSIA (IPM)
  - Dr Patrice PIOLA (IPM)
  - Dr Dolorès POURETTE (IRD/UMR CEPED/Université Catholique de Madagascar)
  - Mme Chiarella MATTERN (IPM)
  - Mme Emma RABOANARY (IPM)
  - Mr Livah RABEARISON (IPM)
  - Mme Tahiry RABEANDRIAMARO (IPM)

- **Responsables scientifiques :**
  - Pr Christophe ROGIER (IPM)
  - Dr Dolorès POURETTE (IRD/UMR CEPED/UCM)
  - Dr Frédéric SANDRON (IRD/UMR CEPED)
  - Pr Maryse GAIMARD (Université de Dijon)

REMERCIEMENTS

L'équipe de recherche remercie vivement :

- Pr Sophie GOEDEFROIT (Université Paris-Descartes/UMR CEPED), Représentante de l'IRD à Madagascar
- Pr Christophe ROGIER, directeur de l'IPM
- Mr Thomas KESTEMAN (IPM) et toute l'équipe d'investigation MEDALI
- Le Programme National de Lutte contre le Paludisme (PNLP)
- Le Ministère des Affaires Etrangères et européennes (MAEE)
- Le Fonds mondial de lutte contre le sida, la tuberculose et le paludisme (FM)
- France Expertise Internationale (FEI)
- L'ensemble des enquêtés et des personnes nous ayant facilité l'accès aux terrains

RESUME

Objectifs : L'objectif général de cette étude exploratoire est d'étudier les facteurs socio-comportementaux interagissant avec les interventions de lutte antipaludique, entravant ou facilitant leur efficacité. Il s'agit notamment d'identifier les raisons explicatives du recours aux formations sanitaires en cas de fièvre, de la prise du TPI (traitement préventif intermittent des femmes enceintes), et de l'acceptation des pratiques de préventions proposées (CAID – aspersion d'insecticide intra-domiciliaire et MID – moustiquaire imprégnée d'insecticide).

Méthodologie : Des enquêtes qualitatives par entretiens semi-directifs et observations ont été menées dans 4 sites situés dans des contextes épidémiologiques variés : Moramanga, Antsohihy, Fianarantsoa, Mananjary. 70 entretiens ont été réalisés, dont 18 avec des prestataires de soins. Les entretiens exploraient trois thématiques : les perceptions et pratiques usuelles en cas de fièvre (chez l'adulte et l'enfant) ; les pratiques de prévention du paludisme ; la connaissance et l'accès au traitement.

Résultats :

Des perceptions floues du paludisme

Dans les quatre zones, les perceptions populaires confondent le « paludisme » (*tazomoka*) et les fièvres (*tazo*). Le lien entre le vecteur et la maladie n'est pas clair. Dans les perceptions, *tazomoka* n'est pas uniquement dû aux piqûres de moustiques, mais aussi au manque d'hygiène, à la fatigue (Moramanga), au fait de manger certains fruits (Fianarantsoa). Quel que soit le faciès épidémiologique de la zone étudiée, le paludisme n'est pas perçu par la population comme un problème de santé grave ou préoccupant. Les femmes enceintes et les enfants de moins de 5 ans sont perçus comme étant particulièrement vulnérables.

Faible recours aux formations sanitaires et retard aux soins

Les principaux problèmes dans la prise en charge du paludisme restent le faible recours aux formations sanitaires et le retard aux soins. Ce retard relève de plusieurs causes : les difficultés financières, l'éloignement des structures de santé, le manque d'information, les recours usuels en cas de maladie (automédication, puis recours au guérisseur traditionnel et/ou à l'Agent communautaire, puis recours ultime aux structures de santé s'il n'y a pas d'amélioration).

Le TPI : méconnaissances des femmes et indisponibilité des traitements

Dans les quatre zones étudiées, rares sont les femmes qui affirment avoir pris le traitement préventif contre le paludisme pendant leur grossesse Soit elles ont pris des médicaments sans qu'on leur explique de quoi il s'agissait et sans qu'elles s'en souviennent, soit le TPI n'a pas été donné (des soignants ont évoqué des ruptures de stocks). De manière générale, on observe un faible niveau de connaissances des patients sur leur maladie et les médicaments, en raison des difficultés de communication entre soignants et soignés.

La MID : une utilisation variable, rarement en lien avec la protection contre le paludisme

L'utilisation des moustiquaires est justifiée en premier lieu par la gêne occasionnée par les moustiques. La protection contre le paludisme est citée comme deuxième raison d'utilisation de la MID à Mananjary seulement. De nombreux facteurs favorisent ou limitent l'utilisation des MID : la température, le vent, la luminosité, la préservation de l'intimité du couple, l'impossibilité pour les enfants de sexes différents de dormir sur la même couche à partir d'un certain âge (variable selon les zones). Lorsque le nombre de MID est insuffisant dans un ménage, ce sont les enfants de moins de 5 ans et les femmes enceintes qui dorment en priorité sous la moustiquaire.

Le message de sensibilisation selon lequel « il faut dormir sous moustiquaire » est bien retenu par la population, mais la raison de cette nécessité (se protéger contre le paludisme) n'est pas toujours

intégrée. Les consignes d'utilisation et de lavage ne sont pas retenues ni appliquées dans leur intégralité. La durée d'efficacité du produit n'est pas connue.

La CAID : de nombreux désagréments et une efficacité rarement perçue

Le lien entre la CAID et le moustique (et à fortiori la lutte contre le paludisme) n'est pas fait. La CAID est en effet perçue comme un moyen de lutter contre les petits insectes (puces et cafards principalement), mais pas forcément contre les moustiques. D'autre part, de nombreux désagréments dus aux aspersions ont été rapportés : mauvaises odeurs, maux de tête, dégradations des murs, décès de poules… Enfin, le produit aspergé est souvent perçu comme peu efficace (voire pas efficace du tout) ou efficace mais pendant un temps très limité. Rares sont les personnes qui refusent l'aspersion à leur domicile, car ces campagnes émanent des autorités, et les populations ne veulent pas se distinguer par des comportements récalcitrants.

SOMMAIRE GENERAL

I. OBJECTIFS DE LA MISSION 8

1. Objectif général 8
2. Objectifs spécifiques 8
3. Méthodologie 8
4. Résultats attendus 8
5. Composition de l’équipe en charge du volet qualitatif de MEDALI 8

II. DEROULEMENT DE LA MISSION 9

1. Déroulement des activités 9
2. Activités réalisées 9

2.1. Activites préparatoires 9

2.2 Enquêtes approfondies 12

2.3 Analyses des données 13

III. RESULTATS PAR ZONE 14

I. ZONE 1 : MORAMANGA 14

II. ZONE 2 : ANTSOHIHY 33

III. ZONE 3 : FIANARANTSOA 59

IV. ZONE 4 : MANANJARY 73

IV. LEÇONS APPRISES 97

V. RECOMMANDATIONS/PRECONISATIONS 101

VI. ANNEXES : CANEVAS D’ENTRETIENS 104

ACRONYMES ET ABREVIATIONS

AC : Agent de santé communautaire
ACT : Artemisinin based combination therapy
ADRA : Adventist Development Relief Agency
CAID : Campagne d'aspersion intra-domiciliaire
CPN : Consultation prénatale
CSB : Centre de santé de base
MEDALI : Mission d'Etudes des Déterminants de l'Accès aux méthodes de Lutte antipaludique et de leur Impact
MID : Moustiquaire Imprégnée à efficacité durable
ONG : Organisation non gouvernementale
PCIMEc : Prise en charge intégrée des maladies de l'enfant au niveau communautaire
PNLP : Programme national de lutte contre le paludisme
PSI : Population Services International
SSME : Semaine de la santé de la mère et de l'enfant
TDR : Test de diagnostic rapide
TPI : Traitement préventif intermittent

I. OBJECTIFS DE LA MISSION

1. Objectif général

L'objectif général est de réaliser une enquête exploratoire sur les déterminants socio-comportementaux interagissant avec les interventions de lutte antipaludique, entravant ou facilitant leur efficacité.

2. Objectifs spécifiques

- Identifier les déterminants des recours aux formations sanitaires en cas de fièvre
- Identifier les déterminants de la prise du TPI (traitement préventif intermittent des femmes enceintes)
- Identifier les déterminants de l'acceptation des pratiques de préventions proposées (CAID – aspersion d'insecticide intra-domiciliaire et MID – moustiquaire imprégnée d'insecticide)

L'enquête explore trois thématiques :

- Les perceptions et pratiques usuelles en cas de fièvre (chez l'adulte et l'enfant)
- Les pratiques de prévention du paludisme
- La connaissance et l'accès au traitement

3. Méthodologie

Des enquêtes qualitatives par entretiens semi-directifs et observations ont été menées dans 4 sites d'enquête choisis de façon à tenter de maximiser la diversité des contextes épidémiologiques et populationnels (aspects socio-culturels en particulier).

4. Résultats attendus

- une meilleure connaissance des pratiques préventives et curatives en cas de fièvre
- une meilleure connaissance des obstacles à l'adoption des mesures de prévention contre le paludisme et à l'accès aux soins
- des pistes pour orienter les campagnes de prévention et les discours accompagnant les interventions antipaludiques

5. Composition de l'équipe en charge du volet qualitatif de MEDALI

- Dolores Pourette, docteure en anthropologie, chercheure à l'IRD
- Chiarella Mattern, socio-anthropologue IPM
- Emma Raboanary, doctorante IPM
- Farasoa Ravelomandeha (interprétariat)
- Rachel Rasoazafimike (interprétariat)

II.DEROULEMENT DE LA MISSION

1. Déroulement des activités

Les activités se sont déroulées selon le calendrier suivant.

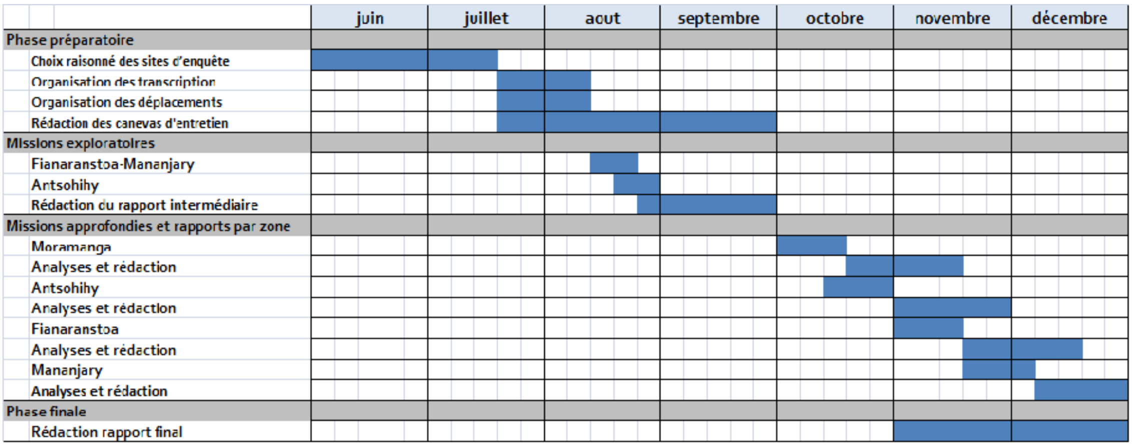

2. Activités réalisées

Cette étude qualitative a été mise en place en deux phases : une phase d'enquête exploratoire et une phase d'enquête approfondie. Au préalable, une revue de la littérature sur la thématique précise du paludisme a été réalisée, ainsi qu'une recherche bibliographique pour les zones investiguées, afin d'identifier les caractéristiques socio-culturelles propres à chacune des zones.

2.1. Activités préparatoires

2.1.1. Identifications des zones

Tout au long de la mise en place du projet MEDALI, le volet qualitatif de l'étude s'est déroulé simultanément au volet quantitatif, les 4 sites d'interventions pour l'étude qualitative ont été sélectionnées parmi les sites de passage de l'équipe quantitative, dans l'objectif de croiser les données recueillies. Cependant, au sein même des zones sélectionnées, le *fokontany* choisi pour l'enquête qualitative différait de ceux investigués par le volet quantitatif dans un souci de ne pas imposer aux villageois les passages de deux équipes d'investigations en une courte période. Parallèlement, nous voulions éviter d'un côté comme de l'autre les interférences entre équipes.

Les 4 zones d'enquête pour le volet qualitatif ont été sélectionnées sur base des différents faciès de paludismes présents sur l'île, dans le but de récolter des informations le plus complètes possibles quant aux modes de préventions disponibles sur l'île. Parallèlement, nous étions guidés par la volonté de saisir le plus large panel possible d'attitudes et

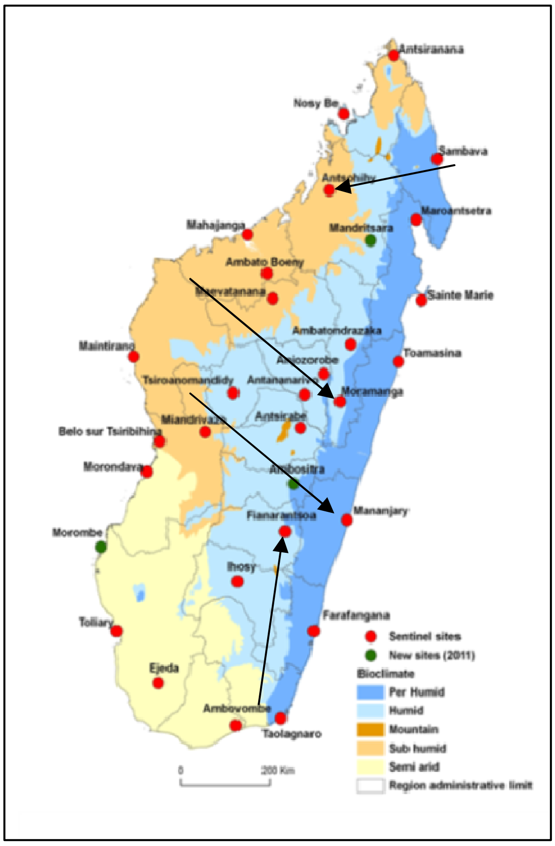

pratiques face à la maladie, au traitement et à la prévention et pour ce faire, choisir des zones aux caractéristiques socio-culturelles différentes (groupe ethnique, situation géographique : côtier/hautes terres, etc). Les zones sélectionnées sont donc les suivantes : Moramanga, Antsohihy, Fianarantsoa et Mananjary (indiquées par des flèches sur la carte).

2.1.2 Enquête exploratoire : introduction sur les sites

L'objectif de cette première phase du projet était de réaliser une reconnaissance des lieux afin de faciliter l'introduction au sein des sites : accessibilité géographique, mise en place de la logistique (identification de logement), introduction au niveau de la communauté, visites de courtoisie et prise de contacts auprès des autorités locales (administratives, traditionnelles, personnel de santé et autres personnes influentes). Il s'agissait également de repérer l'agenda des évènements de la région (marchés, etc.) afin d'assurer une disponibilité maximale des personnes interrogées, ainsi que d'identifier des relais et traducteurs avec lesquels travailler durant les missions approfondies.
Au vu de la diversité des langues locales, des us et coutumes et des cosmogonies qui caractérisent Madagascar, cette étape préalable était incontournable à la bonne mise en place de l'étude approfondie.

A) Introduction auprès des autorités des formations sanitaires et identification des fokontany d'intervention

La rencontre avec les autorités sanitaires (directeur régional de la santé, médecin inspecteur, médecin chef CSB 1 et 2) avait pour double objectif de faciliter l'intégration au sein du district et des communes ainsi qu'à renforcer les collaborations avec les différents niveaux de hiérarchie. Parallèlement, ces rencontres visaient l'identification des *fokontany* d'intervention, au sein même des zones délimitées. A chaque niveau de la pyramide sanitaire, la question du choix du *fokontany* d'intervention était posée au vu des critères de sélection définis :

- le *fokontany* choisi devait être représentatif pour l'ensemble de la région quant à l'accessibilité géographique aux formations sanitaires
- un autre critère est l'observation, par les prestataires de soin interrogés, de comportement de réticences quant aux moyens de prévention ou aux traitements
- enfin, par souci de richesse d'information sur notre thématique, nous demandions à nos interlocuteurs de nous indiquer les *fokontany* où le taux d'incidence du paludisme était le plus élevé (pour des raisons environnementales comme la présence de corridor forestier ou pour des raisons pratiques telles que la présence de lieux de passage largement fréquentés qui pourraient donner lieu au paludisme de voyageurs, *i.e.* importé).

	**ZONES**	**MORAMANGA**	**ANTSOHIHY**	**FIANARANTSOA**	**MANAJARY**
	**COMMUNES**	AMBOHIBARY	AMPANDRIAKILANDY	ANDRAINJATO CENTRE	TSARAVARY
	**FOKONTANY**	BEFOTSY	AMPANDRIAKILANDY	SAHAMENA	AMPASIMBOLA
**CRITERES DE SELECTION**	**SITUATION GEOGRAPHIQUE**	Fokontany installé de part et d'autre d'une route	Zone accessible par la route	Le fokontany longe un corridor forestier	Présence de forêt autour du fokontany
	**PROFIL EPIDEMIOLOGIQUE**	Zone de transmission présumée faible et saisonnière	Zone de transmission présumée moyenne et saisonnière	Zone à transmission nulle ou très faible, contigüe au faciès Est : présence de paludisme importé	Zone de transmission présumée élevée et pérenne

A ce stade-ci de l'introduction sur le terrain, notre équipe se présentait clairement comme travaillant pour l'Institut Pasteur sur une enquête qualitative sur le paludisme. Mais plus nous nous rapprochions des villages d'intervention, plus il fallait être prudent quant à la définition des raisons de notre présence parmi les communautés (en l'occurrence le paludisme) ainsi qu'à l'affiliation avec l'Institut Pasteur. Nous risquions en effet un biais : dérèglement des attitudes habituelles dû à une peur de contrôle (les familles ne dormant pas sous moustiquaires les installeraient uniquement pour la durée de notre passage, les zones peu touchées par le paludisme deviendraient dans les dires des interlocuteurs subitement fortement touchées dans l'espoir d'obtenir de l'aide sous forme financière, etc). Parallèlement, dans les zones familières avec l'Institut Pasteur (Moramanga), nous risquions de bénéficier des nombreuses expériences d'enquêtes et autres au sein du *fokontany*, qu'elles aient été fructueuses et riches pour la population ou au contraire qu'elles aient été vécues comme une mauvaise expérience.

B) INTRODUCTION AUPRES DES AUTORITES LOCALES : ADMINISTRATIVES ET TRADITIONNELLES

Suite aux rencontres avec les autorités sanitaires, nous nous sommes introduites auprès des autorités administratives au niveau communal (maire) et au niveau des *fokontany* et hameaux (chef de *fokontany et chef de village dans les hameaux*). Ensuite, étant donné notre présence durant une durée relative au sein des villages, nous avons également rencontré les autorités traditionnelles tels que les *zokiolona* et a*mpanjaka* afin d'assurer une bonne intégration au sein des *fokontany*.

2.1.3 Préparation des canevas d'entretien

Deux canevas d'entretien ont été conçus : un pour les habitants, un pour les prestataires de soins. Ces canevas ont été testés le premier jour de la première mission sur le site de Moramanga, en collaboration avec deux médecins du PNLP qui nous avaient accompagnés, dans un objectif de

familiarisation aux outils de recherche qualitative et de transfert de compétences. Les canevas sont présentés en annexes.

2.2 Enquêtes approfondies

Le travail d'enquête comprend les entretiens avec les enquêtés, les observations menées dans les villages et au domicile des enquêtés, ainsi que les séances quotidiennes de debriefing menées par l'équipe de recherche et les interprètes.

Afin de favoriser la neutralité des réponses des enquêtés, il a été décidé que l'équipe de recherche ne se présenterait pas au nom de l'Institut Pasteur de Madagascar, institut très connu de la population malgache. Chiarella Mattern, Emma Raboanary et leurs interprètes se sont présentées comme des étudiantes en sociologie de l'Université d'Antananarivo faisant une recherche sur le paludisme. Dolorès Pourette s'est présentée comme anthropologue de l'Institut de Recherche pour le Développement. L'équipe de recherche se déplaçait avec un chauffeur et un véhicule de l'Institut Pasteur de Madagascar. Le logo de l'Institut sur le véhicule a été masqué.

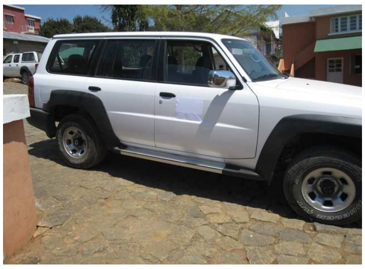

Les entretiens ont été menés à l'aide d'un guide d'entretien. L'anonymat et la confidentialité des données ont été garantis aux enquêtés.

Au total, 70 entretiens formels ont été réalisés, dont 18 avec des prestataires de soins (8 Agents communautaires, 3 médecins, 3 infirmières, 2 guérisseurs traditionnels, 1 matrone, 1 sœur). De nombreuses discussions informelles avec des villageois et d'autres acteurs (épiciers, pasteur, directeur d'école…) ainsi que des observations (des conditions de vie des villageois, de l'utilisation des moustiquaires, des mise en place des CAID…) complètent les entretiens formels.

2.2.1 Site 1 : Moramanga (8-20 octobre 2012)

L'enquête approfondie sur ce premier site a dans un premier temps constitué une phase de test des canevas d'entretien et de la méthodologie et une phase de rodage de l'enquête. L'équipe de recherche était au complet pour cette phase de test et de rodage, afin de pouvoir réajuster les outils si nécessaire. Cette phase a en outre constitué une session de familiarisation aux outils qualitatifs et de transfert de compétences pour les deux médecins du PNLP qui nous ont accompagnées. L'enquête a dans un second temps été menée par Dolorès Pourette et Emma Raboanary (interprétariat).

Au total, 17 interviews ont été réalisées : 4 avec des prestataires de soins, 7 avec des femmes[1], 6 avec des hommes[2].

[1] 2 femmes entre 25 et 29 ans, 2 femmes entre 30 et 39 ans, 2 femmes entre 50 et 59 ans, 1 femme de 66 ans.
[2] 1 homme de 24 ans, 3 hommes entre 30 et 39 ans, 1 homme de 61 ans, 1 homme de 80 ans.

2.2.2 Site 2 : Antsohihy (18-30 octobre)

L'enquête a été réalisée par Chiarella Mattern et Farasoa Ravelomandeha (interprétariat). Au total 20 entretiens ont été réalisés : 6 avec des prestataires de soin, 7 avec des femmes[3], 7 avec des hommes[4].

2.2.3 Site 3 : Fianarantsoa (5-19 novembre 2012)

L'enquête a été conduite par Emma Raboanary et Rachel Rasoazafimike (interprétariat). Au total 18 entretiens ont été réalisés : 4 avec des prestataires de soin, 7 avec des femmes[5], 7 avec des hommes[6].

2.2.4 Site 4 : Mananjary (21 novembre-2 décembre 2012)

L'enquête a été menée par Chiarella Mattern et Farasoa Ravelomandeha (interprétariat), avec la supervision de Dolorès Pourette. Au total 15 entretiens ont été réalisés : 4 avec des prestataires de soin, 5 avec des femmes[7], 6 avec des hommes[8]. Douze entretiens informels ont été réalisés dans le cadre de l'évaluation de la distribution de moustiquaires.

2.3 Analyses des données

Tous les entretiens ont été transcrits dans leur intégralité, puis analysés selon la méthode classique d'analyse thématique utilisée dans les enquêtes qualitatives.

[3] 2 femmes entre 20 et 30 ans, 2 entre 30 et 40 ans, 3 entre 50 et 65 ans.
[4] 1 homme entre 20 et 30 ans, 4 entre 30 et 40 ans, 1 entre 40 et 50 ans, 1 entre 50 et 65 ans.
[5] 4 femmes entre 20 et 29 ans, 1 femme de 49 ans, 2 femmes entre 50 et 59 ans.
[6] 2 hommes entre 20 et 29 ans, 2 entre 40 et 49 ans, 1 de 58 ans, 2 entre 60 et 69 ans.
[7] 1 femme entre 20 et 29 ans, 2 entre 30 et 39 ans, 2 entre 50 et 65 ans.
[8] 2 hommes entre 20 et 29 ans, 3 entre 30 et 40 ans, 1 entre 50 et 65 ans.

III. RESULTATS PAR ZONE

I. ZONE 1 : MORAMANGA

Le rapport sur la zone d'étude de Moramanga a été rédigé par Emma Raboanary.

Table des matières

1. Contexte et generalites **16**
1.1 Localisation géographique 16
1.2 Contexte épidémiologique du paludisme à Moramanga 17
1.2.1 Situation du paludisme à Moramanga 17
1.2.2 Situation du paludisme dans la commune d'Ambohibary 17
1.3 Justification du choix de fokontany 17
1.3.1 Enjeu de la continuité de la lutte contre le paludisme dans le *fokontany* 17
1.3.2 Choix du *fokontany* Befotsy 18
2. Resultats **19**
2.1. Connaissances de la population sur le paludisme 19
2.1.1 Le paludisme n'est pas perçu comme un problème de santé au sein de la communauté 19
2.1.2 Le paludisme concernerait surtout les femmes enceintes et les enfants de moins de 5 ans 19
2.1.3 Connaissances partielles sur les symptômes du paludisme 19
2.1.4 Diverses interprétations autour des causes du paludisme 20
2.2 Difficultés rencontrées par les soignants dans le diagnostic et la prise en charge du paludisme 21
2.2.1 Rupture de stock de TDR 21
2.2.2 Reflexes thérapeutiques de la population en cas de fièvre non favorable à la détection précoce du paludisme 21
2.2.3 Précisions concernant l'automédication en cas de fièvre 22
2.2.4 Recours à la médecine traditionnelle 23
2.2.5 Problème de légitimité des agents communautaires et limites de leurs fonctions 24
2.2.6 Connaissances par rapport au traitement du paludisme 24
2.3 Les pratiques préventives de la population 27
2.3.1 La MID 27
2.3.2 La CAID 29
2.3.3 Le TPI 29
3. Conclusions **32**

1.1 Localisation géographique

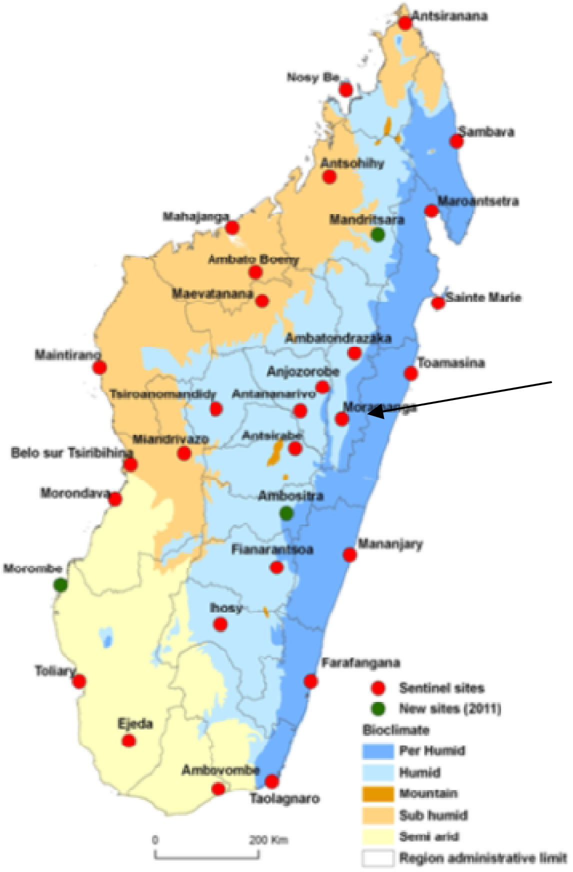

Nous avons mené notre enquête dans le *fokontany* Befotsy qui se trouve dans la Commune rurale d'Ambohibary à environ 10 km au Nord Est de Moramanga Ville (se trouvant sur la Route Nationale 44). Deux hameaux ont été sélectionnés : Befotsy, le chef-lieu de la commune, et Mahazina, un hameau se situant à 5km à l'Ouest du chef-lieu. Ce dernier est plus éloigné des formations sanitaires et est entouré de rizières et de forêt. Les populations locales vivent essen-tiellement de l'agriculture et les femmes trouvent leur revenu dans le tissage et la vannerie.

Il existe deux infrastructures sanitaires au sein de la Commune Rurale d'Ambohibary : le CSB1 d'Ambodiakatra et le CSB2 se situant dans le chef lieu même c'est à dire à Ambohibary.
Les distances de parcours entre les hameaux sélectionnés et ces deux types de structures de soin sont présentées approximativement dans le tableau suivant :

Source : Institut Pasteur de Madagascar, Rapport Sites sentinelle, 2011

	Distance
**(chef-lieu) Befotsy – CSB1 Ambodiakatra**	Environ 1km
**(chef-lieu) Befotsy - CSB2 Ambohibary**	Environ 3km
**Mahazina – CSB1 Ambodiakatra**	Environ 6km
**Mahazina – CSB2 Ambohibary**	Environ 8km

Estimation distance du trajet entre les 2 hameaux et les 2 Formations sanitaires publiques dans la commune d'Ambohibary, terrain en octobre 2012.

Nous pouvons voir à travers ce tableau que le hameau Mahazina est 6 fois plus éloigné du CSB1, et 3 fois plus éloigné du CSB2 que Befotsy se trouvant à proximité du grand axe de la route nationale 44. Cette situation illustre bien deux réalités différentes en matière d'accessibilité géographique au sein d'un même fokontany à plusieurs hameaux. En saison

sèche, les villageois accèdent à pied ou à bicyclette aux formations sanitaires publiques précitées (aussi bien pour les villageois du Mahazina que ceux du chef lieu), l'accessibilité géographique pose peu de problème. En revanche la piste est moins praticable en saison des pluies surtout pour le cas de Mahazina, ce qui peut prolonger la durée du trajet.

Les difficultés d'accès géographique se posent notamment pour les malades, qui sont dans l'incapacité physique d'effectuer un tel trajet à pied ou à bicyclette, ce qui nécessite de mobiliser d'autres ressources (humaines, de temps, financières…), et donc plus d'investissements.

1.2 Contexte épidémiologique du paludisme à Moramanga

1.2.1 Situation du paludisme à Moramanga

Comme nous l'avons déjà signalé auparavant, Moramanga relève du faciès du paludisme des marges et est caractérisé par une transmission présumée faible et saisonnière. Les stratégies de lutte mises en place sont les suivantes : la CAID, la MID, le TPI, le PCIMEc.

1.2.2 Situation du paludisme dans la commune d'Ambohibary

D'après les perceptions et observations de certains soignants, le nombre de cas de paludisme a considérablement diminué vers l'année 2005 dans la commune d'Ambohibary. Depuis quelques années ils constatent qu'il n'y a plus que 2 à 3 cas par an au niveau des formations sanitaires publiques[9] de la commune. Selon leur analyse, cette baisse est associée à l'application effective des stratégies de lutte préventive mises en place par la politique nationale de santé, à savoir l'utilisation de la MID, la réalisation de la CAID, l'utilisation du TDR qui a facilité le diagnostic et la prise en charge du paludisme.

1.3 Justification du choix de fokontany

1.3.1 Enjeu de la continuité de la lutte contre le paludisme dans le fokontany

Tant pour les soignants que pour les villageois du *fokontany*, le paludisme ne semble plus être un problème de santé.

> « *Avant le paludisme était un problème important mais actuellement il diminue* » [Soignant, 38 ans]
>
> « *Avant, j'ai habité à Antsihanaka, à l'ouest d'Ambatondrazaka, là bas il y avait beaucoup de tazomoka…Mes enfants y ont attrapé cette maladie, il y a une dizaine d'année, alors j'avais acheté une moustiquaire suivant le conseil du médecin, et je l'ai utilisée. Ce n'était qu'après qu'il y avait eu une campagne de distribution de moustiquaire…Ici, il y a beaucoup moins de tazomoka. On nous avait également donné gratuitement des moustiquaires. On les utilise pendant la saison des pluies. On nous les a distribuées*

[9] Ce qui n'informe pas les éventuels cas qui peuvent aller dans les structures sanitaires privées ou des formations sanitaires publiques qui se situent à une échelle de pyramide plus supérieure. Données seulement limitée au niveau des deux formations sanitaires publiques de la commune.

gratuitement, donc nous devons les utiliser…Cela ne me dérange pas du tout, au contraire on peut dormir tranquillement parce que les moustiques ne peuvent pas nous déranger » [Femme, 53 ans]

L'hétérogénéité de l'épidémiologie du paludisme est sous l'influence de différents facteurs, à savoir le rôle de la distribution des anophèles et leur capacité vectorielle, les conditions bioclimatiques et environnementales qui conditionnent le développement des anophèles vecteurs potentiels du paludisme, mais aussi l'immunité acquise à l'occasion des infections plasmodiales.

La continuité de la lutte contre le paludisme est cruciale, malgré le fait qu'elle soit perçue par la population et par les soignants comme une maladie peu inquiétante. Selon J. Mouchet, et. al, en 2004[10], dans ce type de zone, la transmission peut varier d'une année à l'autre, certaines années peuvent être favorables au développement des vecteurs, d'autres pas. Cependant, toujours selon lui, une interruption des mesures de lutte pourrait provoquer une poussée épidémique, si la transmission n'est plus artificiellement freinée par les luttes antivectorielles ou chimioprophylactiques. L'acquisition de l'immunité de la population peut être inexistante ou limitée, si bien que toutes les personnes sont exposées au risque de paludisme, sans distinction d'âge, de sexe ou de condition (*e.g.* pendant la grossesse ou en dehors de la grossesse).

1.3.2 Choix du fokontany Befotsy

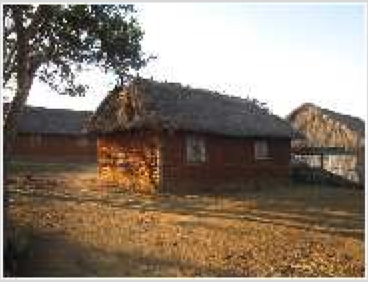

HAMEAU MAHAZINA
PHOTO, D. POURETTE, OCT.2012

Le choix du *fokontany* Befotsy s'est fait en fonction de l'accessibilité géographique par rapport aux structures de soins, ainsi qu'en fonction de l'intérêt de pouvoir mettre en évidence les diverses connaissances, attitudes et pratiques adoptées face au contexte épidémiologique du paludisme spécifique de cette zone.

[10] J. Mouchet, et al. 2004, *Biodiversité du paludisme dans le monde.* John Libey Eurotext. Disponible sur : [http://books.google.mg/books?id=qz1cXrXTFpwC&printsec=frontcover&hl=fr#v=onepage&q&f=false]

2.1. Connaissances de la population sur le paludisme

2.1.1 Le paludisme n'est pas perçu comme un problème de santé au sein de la communauté

Les principales maladies citées par les personnes que nous avons enquêtées étaient le *tazo* ou la fièvre, viennent ensuite la diarrhée, la grippe, la toux les maux de dents. Les personnes enquêtées n'ont pas évoqué spontanément le paludisme parmi les maladies les plus fréquentes dans le village. C'est lorsque nous leur avons demandé s'ils connaissaient des maladies transmises par les moustiques qu'ils ont mentionné le *tazomoka*, un terme connu mais peu utilisé, aussi bien par les habitants du chef lieu, que ceux du hameau de Mahazina[11]. Le paludisme est donc une maladie qui n'est pas perçue comme un problème de santé à l'heure actuelle.

> « *Avant il y avait beaucoup de tazomoka dans le fokontany, actuellement il n'y en a presque plus, c'est peut-être lié par le fait que les gens dorment tous sous moustiquaire* » [Femme, 25 ans]

2.1.2 Le paludisme concernerait surtout les femmes enceintes et les enfants de moins de 5 ans

Le message le plus fréquemment retenu par les interlocuteurs, parmi ceux diffusés lors des campagnes de distribution des MID, est la plus grande vulnérabilité des femmes au *tazomoka*, notamment les femmes enceintes, et celle des enfants de moins de 5 ans. Pour la majorité, cette maladie est souvent perçue comme un problème de santé qui touche principalement, voir exclusivement les femmes enceintes et les enfants de moins de 5 ans. Un homme, par exemple, ne se sentait pas concerné par l'utilisation de la moustiquaire, puisqu'il n'a pas d'enfant.

> « *Je n'utilise pas une moustiquaire, parce que je n'ai pas d'enfant. En plus je suis un homme, je ne tombe pas souvent malade* » [Homme, 24 ans].

2.1.3 Connaissances partielles sur les symptômes du paludisme

Les connaissances des enquêtés sur les symptômes du paludisme sont partielles. Les symptômes mentionnés sont les suivants : température élevée, corps chaud, sensation de froid, céphalées, frissonnement, tremblement, vomissement et anorexie. Certains ajoutaient la sensation d'avoir les jambes lourdes et l'apparition de boutons rouges surtout sur le visage. L'apparition de boutons rouges était surtout évoquée dans le hameau Mahazina. Cela peut s'expliquer par le fait que la population est plus exposée aux piqûres de moustiques, du fait qu'elle vit à proximité de la rizière et des forêts par rapport aux villageois qui habitent

[11] Lorsqu'ils ont été questionné sur les différentes sortes de *tazo* ou fièvre, les enquêtés ont systématiquement évoqué *tazo tsotra* et *tazomahery*. Ce n'est qu'après avoir demandé s'ils connaissaient une maladie transmise par le moustique qu'ils ont évoqué le terme *tazomoka.*

dans le chef lieu. Ces observations démontrent une certaine confusion entre les symptômes de la maladie et les traces laissées par des moustiques, pas forcément vecteurs du paludisme.

Les symptômes du paludisme les plus fréquemment identifiés peuvent être associés au *tazo tsotra* ou fièvre simple, mais non au paludisme grave. Cette observation rejoint les propos de certains soignants qui affirment qu'il existe une confusion parmi la population entre la fièvre et le paludisme.

2.1.4 Diverses interprétations autour des causes du paludisme

2.1.4.1 Causes hygiéniques et environnementales

Si la majorité des enquêtés connaissent le *tazomoka* comme maladie transmise par le moustique, d'autres causes du *tazomoka* ont été évoquées : la malpropreté et le manque d'hygiène. En effet, dans les messages véhiculés pour la prévention du paludisme, à part l'adoption des diverses luttes préventives comme l'utilisation de la MID et la réalisation de la CAID, on sensibilise la population à débroussailler, à combler les flaques d'eau pour les éliminer afin d'éviter la prolifération des moustiques. Ces messages sont présents lors des sensibilisations et des campagnes de lutte contre d'autres maladies[12].

Ainsi certains enquêtés ont affirmé que le *tazomoka* pouvait aussi être dû à la malpropreté, d'autres pensent qu'on peut être affecté par cette maladie lorsqu'on ne se lave pas avec du savon. Par ailleurs, quelques-uns émettent l'hypothèse que le paludisme pourrait se transmettre par l'air impur que l'on respire ou par l'eau que l'on boit.

2.1.4.2 Autres causes évoquées

Certains affirment que le paludisme « *peut apparaître comme ça* » sans qu'il y ait une raison identifiable. Cette réponse met plutôt en évidence un manque d'explication et de compréhension par rapport à la maladie de la part des enquêtés.

D'autres disent que le paludisme peut être dû à une grosse fatigue, au fait d'avoir beaucoup travaillé dans les champs. La fatigue pourrait constituer un terrain propice au paludisme. Ce genre de réponse n'est pas seulement propre à Madagascar, elle s'observe également dans d'autres pays d'Afrique, comme en Côte d'Ivoire[13]. Ceci est notamment dû au fait que les activités quotidiennes en milieu rural sont concentrées dans les travaux des champs, ce qui implique des efforts physiques intenses.

Certains expliquent que les enfants seraient plus vulnérables que les adultes face au paludisme par le fait que les adultes auraient une meilleure condition physique. Même en cas de piqûre de moustique, ils ne risqueraient pas d'être affectés par cette maladie s'ils ont le « *vatana salama* » ou corps sain, qu'ils définissent comme le fait d'être bien nourri et en bonne santé.

---

[12] Diarrhée, malnutrition, infection respiratoire aigues etc.…

[13] B. Kouakou, 2011, L'étiologie socioculturelle comme fil conducteur de la lutte communautaire contre le paludisme en Côte d'Ivoire. AUF. Cahier Santé. Vol 21 N°2.

Selon des enquêtés, l'existence d'une affection pathologique quelconque[14] peut faire apparaître ou aggraver le paludisme. Ils évoquent souvent l'expression *atody tazo* dont la traduction littérale serait « œufs de la fièvre ». Ceci signifie que l'on « couve » une maladie à l'intérieur de soi et qu'il suffit qu'il y ait un état pathologique pour faire apparaître la fièvre. Ceci laisse refléter que pour certains, le paludisme serait aussi une maladie déjà présente dans le corps et qui se cacherait derrière d'autres maladies.

2.2 Difficultés rencontrées par les soignants dans le diagnostic et la prise en charge du paludisme

2.2.1 Rupture de stock de TDR

Les ruptures de stock de TDR ont été signalées dans le CSB, ce qui porte un impact sur la détection des cas de paludisme, voir le contrôle d'une épidémie au sein d'une communauté. En outre, les agents communautaires enquêtés ne disposaient pas de TDR.

2.2.2 Reflexes thérapeutiques de la population en cas de fièvre non favorable à la détection précoce du paludisme

Comme nous l'avons évoqué plus haut, le retard de prise en charge du paludisme est évoqué comme un problème majeur par les soignants, les malades peuvent arriver au centre de santé 3 à 4 jours après l'apparition des premiers symptômes de cette maladie. Selon les soignants, ceci est dû à la confusion entre la fièvre et le paludisme, au recours à l'automédication et à la consommation de plantes médicinales, qui sont des modes de prise en charge perçus comme moins couteux. Au-delà des obstacles financiers, les soignants associent surtout ces comportements aux mauvaises habitudes et à un manque d'instruction.

En effet, après le recoupement des informations auprès des populations, le recours au centre de santé apparaît comme l'ultime recours. Les personnes souffrantes ne se rendent dans un centre de santé que lorsqu'elles constatent que la maladie s'aggrave, au bout de plusieurs jours. Plusieurs scénarios peuvent se présenter en cas de fièvre chez les villageois : soit ils ont recours à l'automédication, soit ils attendent en espérant que la fièvre passe, puisqu'elle est souvent liée au froid ou aux grosses fatigues, soit ils consomment une plante pour atténuer la maladie en attendant de trouver de l'argent pour aller au centre de santé.

> « *On attrape le tazo à cause de la fatigue, j'attends 3 jours pour voir si ça passe, je me repose un petit peu, en même temps je consomme de l'aferontany, en général ça passe au bout de 3 jours* » [Femme, 66 ans]

On peut voir à travers ce discours que les pratiques de soins sont ancrées dans les expériences personnelles, de la maladie et de soins. Les individus se fient donc à leurs propres expériences et recourent à des pratiques connues, jusqu'au moment où la maladie s'aggrave et que la situation dépasse leur savoir-faire. Souvent la fièvre est banalisée par les populations. Ces conceptions et reflexes thérapeutiques contribuent à une détection retardée du paludisme.

[14] Sans qu'ils aient précisé de quelles maladies il s'agit après leur avoir demandé.

Par ailleurs, les difficultés financières des familles ont été identifiées par les soignants comme un obstacle à l'accès aux soins, comme le montre le cas relaté ci-dessous.

> Deux enfants de moins de cinq ans venant d'un hameau d'un *fokontany* situé à quelques kilomètres au nord de Befotsy, sont décédés du paludisme en 2011 dans l'une des deux formations sanitaires publiques.
> *« L'enfant avait eu beaucoup de fièvre, on a fait le test, le TDR était positif, c'était du paludisme grave. Le traitement devait se faire par injection, mais ça coutait 5000 ariary et la famille n'avait pas les moyens pour en acheter. Quelques heures après il n'a pas survécu, il était mort ! C'était trop tard…la famille me disait que le petit garçon avait déjà eu la fièvre 4 jours avant de l'emmener ici, la formation sanitaire se trouvait en même temps loin du village. Ils ont cru que c'était une simple fièvre et lui ont donné du paracétamol, mais la fièvre disparaissait et réapparaissait, puis ça s'est aggravé… Les gens négligeaient les agents communautaires, peut être parce que ce ne sont pas des médecins»* [Soignant, 38 ans].

Mais les explications plus approfondies vont au-delà du manque d'instruction, des mauvaises habitudes et des difficultés financières. D'autres obstacles au recours aux soins ont été signalés, comme le manque de temps. Il apparaît enfin que la santé n'est pas une priorité comparée à la nécessité de travailler pour survivre.

L'absence des agents de santé (notamment lorsqu'ils ont une activité à l'extérieur du fokontany) oblige les malades à aller dans d'autres formations sanitaires et implique la mobilisation de plus de ressources (financières, humaines et en temps) donc plus d'investissements. L'isolement de certains *fokontany*/hameaux est un autre facteur contribuant à augmenter les couts indirects liés à la recherche de soins.

2.2.3 Précisions concernant l'automédication en cas de fièvre

En cas de fièvre, les individus ont affirmé qu'ils prennent du diclofénac, du paracétamol, du cotrim (cotrimazole), de l'aspirine, du paludar® (sulfadoxine pyriméthamine), du palustop®, de la chloroquine, ou de l'amoxicilline. Ces médicaments sont disponibles en pharmacie mais aussi dans certaines épiceries.

> « *En cas de tazo je vais chez l'épicier et je lui demande quel est le médicament que je dois prendre. C'est lui qui me conseille d'utiliser tel ou tel médicament* » [Femme, 59 ans]

La prescription d'un épicier pour traiter la fièvre dépend de la maladie avec laquelle elle est associée. Par exemple : du diclofénac en cas de rage de dents. Ce dernier compte sur son expérience personnelle pour donner des conseils à ses clients. Il donne aussi du paludar® en cas de fièvre. Ceci s'avère être une pratique à risque si le traitement ne correspond pas à la maladie.

Enfin, un autre scénario évoqué par certaines personnes est le recours à la médecine traditionnelle suite à l'échec du traitement à base de médicaments « modernes » : la consommation des plantes si le *tazo* ou la fièvre ne part pas au bout de 3 jours. Le traitement précédent sera alors perçu comme inefficace.

Ceux qui ont déjà eu le paludisme et qui ont été guéris par les professionnels de la santé font davantage confiance en ces derniers plutôt qu'en d'autres stratégies comme l'automédication par exemple.

Certains affirment qu'ils préfèrent consommer des plantes en espérant que la fièvre disparaisse afin d'éviter les dépenses. Ceux-ci ne consultent que si la maladie persiste.

Quelques noms de plantes utilisées en cas de fièvre ont été citées : *Aferontany, mandresy*, *ravina valangirina* pour lutter contre la douleur des muscles en cas de *tazo, ravintsara* contre le mal de tête. En ce qui concerne le dosage, chacun fait à sa manière. Le risque dans cette pratique réside dans le fait que les gens ne seront pas en mesure de connaître et de contrôler le degré de toxicité et les effets réels des plantes.

Selon certains soignants, l'automédication et le retard au recours aux soins s'observent sur toute la commune. Selon eux, ce premier réflexe d'automédication n'est pas valable uniquement pour le paludisme ou la fièvre mais pour d'autres maladies également : diarrhée, toux, grippe, etc.

2.2.4 Recours à la médecine traditionnelle

Selon un agent communautaire, certains villageois auraient recours à des devins guérisseurs pour soigner le paludisme qui leur prescrirait des plantes telles que les *aferontany*. Ces individus associent souvent les maladies à l'ensorcellement ou aux mauvais sorts. Les villageois affirment qu'il existe des devins guérisseurs, mais nous n'avons pas recueilli d'autres discours de la part de la population par rapport à cette fréquentation des soignants traditionnels. C'est un sujet qui semble être difficile à aborder notamment pour des raisons d'illégalité de la pratique de la médecine traditionnelle et des raisons religieuses.

> « *Il y a un tradipraticien ici, mais je ne peux pas vous le montrer parce que j'ai peur que ça va se retourner contre moi, parce que peut-être qu'il n'a pas envie qu'on sache ce qu'il fait* » [Agent communautaire, 27 ans]

> *« Oui il y a un devin guérisseur ici, certains y vont parce qu'ils pensent souvent qu'ils ont été ensorcelés en cas de maladie. Je n'y vais pas parce que je suis croyante. Chacun a sa propre vie je ne rentre pas dans la vie des autres »* [Femme, 33 ans]

2.2.5 Problème de légitimité des agents communautaires et limites de leurs fonctions

D'après certains soignants, « les gens négligent les agents communautaires… », au sens où ils ne les considèrent pas comme des acteurs de santé.
Ceci semble être confirmé par le propos de certains villageois qui ont évoqué que ces acteurs sont en effet perçus comme des gens « ordinaires »[15] qui prescrivent en même temps des médicaments.

> *« Les gens d'ici veulent voir de leur propre yeux que ces agents communautaires ont des matériels, un bureau pour travailler en tant qu'agent de santé ce sont des gens comme nous »* [Homme, 38 ans].

Cette négligence nécessite une remise en cause sur la perception de la population et leur relation de confiance entre les agents communautaires qui constituent à la base, un terrain propice à la diffusion et à la réception des messages en matière de comportements de lutte contre les différentes maladies y compris le paludisme.

Les agents de santé communautaires enquêtés n'ont ni TDR ni ACT à disposition. Ils ne peuvent donc pas diagnostiquer le paludisme ou le traiter.

De ce fait, ils préconisent du paracétamol ou de l'aspirine en présence d'un enfant fiévreux et ne pensent à une suspicion de paludisme que si la fièvre ne disparaît au bout de 3 jours. Dans ce cas, ils renvoient les malades dans une formation sanitaire publique.
Les agents de santé communautaires sont demandeurs de davantage de formations, et souhaiteraient avoir plus de responsabilités dans le diagnostic et la distribution des médicaments. Ce problème aggrave leur perte de légitimité.

2.2.6 Connaissances par rapport au traitement du paludisme

La majorité des personnes enquêtées ne connaissaient pas le nom du médicament ACT. La méconnaissance du nom des médicaments a plusieurs explications. D'une part les patients semblent ne pas demander de précisions quant au traitement lorsqu'ils vont chez les médecins. D'autre part, comme nous l'avons vu ci-dessus, les agents communautaires du *fokontany* n'ont pas eu de formation en matière d'utilisation de TDR et n'ont pas à leur disposition de l'ACT si bien qu'ils ne peuvent pas renforcer les connaissances en matière de prise en charge du paludisme. Enfin, les soignants percevant les malades comme étant moins instruits, il semblerait que rares sont les informations transmises concernant les traitements.

[15] Des personnes vues de la même hauteur que les habitants du village

Recours à l'automédication en médicaments modernes ou traditionnels

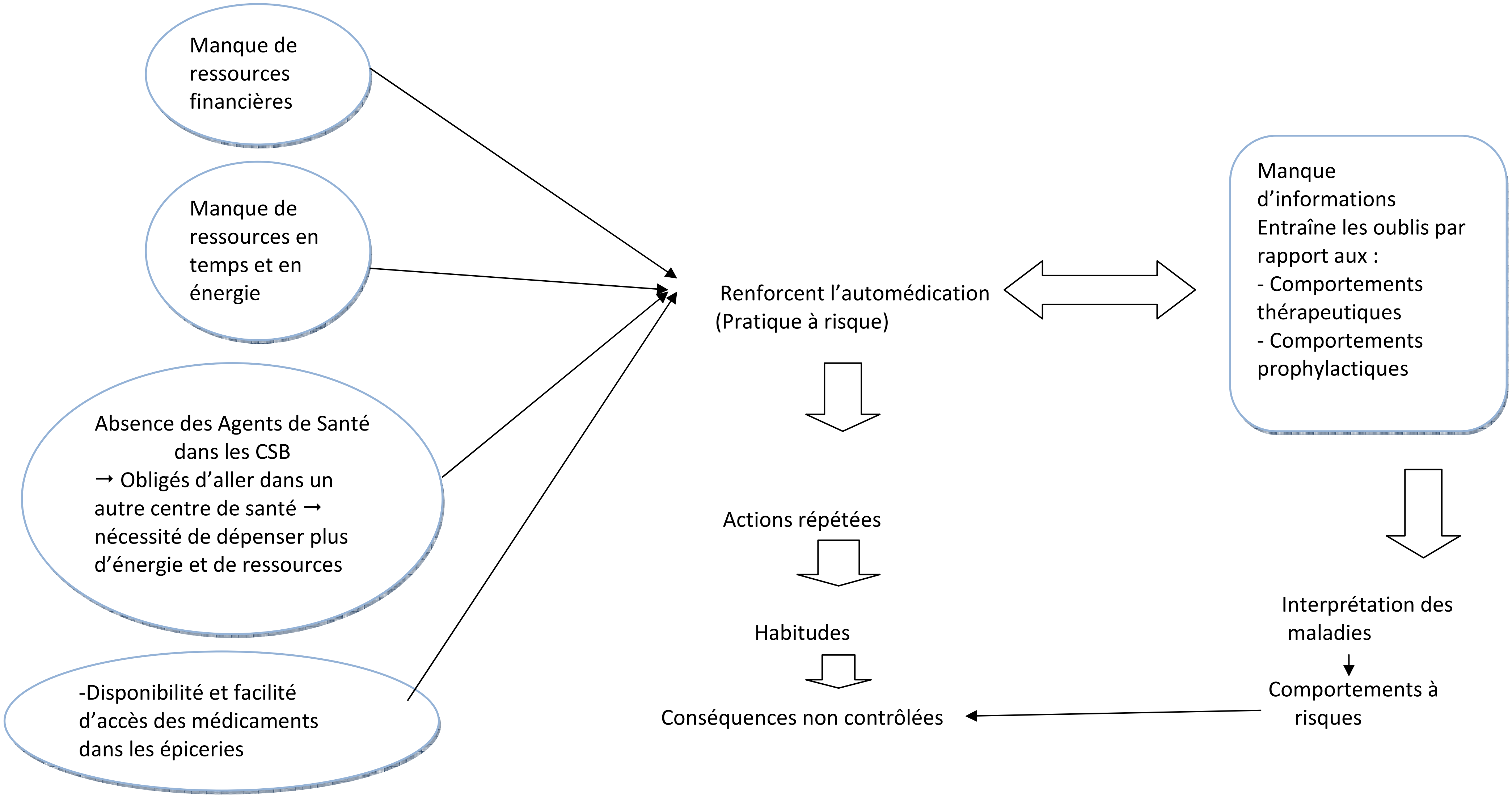

Attente avant le recours aux soins

Manque de ressources financières

Attendre le temps de trouver de l'argent

Utilisation des plantes en espérant pouvoir atténuer la maladie, le temps de trouver de l'argent pour aller dans un Centre de Santé

On espère que la maladie va passer après quelques jours. « Tazo » et fatigue souvent associés : donc attente

Si la fièvre ne part pas au bout de 3 jours (après avoir pris les médicaments prescrits par l'AC en cas de fièvre) : suspicion de paludisme par l'AC (absence de TDR et d'ACT)

Risques aggravation de la maladie
Plus de dépense pour soigner la maladie

2.3 Les pratiques préventives de la population

2.3.1 La MID

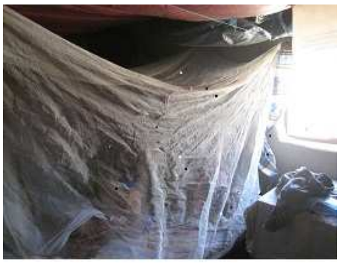

En général, les villageois que nous avons enquêtés ont affirmé avoir reçu des moustiquaires pendant la campagne de distribution qui a eu lieu en 2010 et ont compris qu'elle doit être utilisée pour se protéger contre les piqûres de moustiques. Lors de notre passage[16] dans les habitations, nous avons pu observer que la majorité n'installait pourtant pas les moustiquaires, et disait préférer les mettre pendant la saison des pluies durant laquelle il y a une prolifération des moustiques. Les personnes enquêtées connaissent cependant les consignes selon lesquelles : « il faut dormir sous moustiquaire » surtout concernant les enfants de moins de 5 ans et les femmes enceintes.

Ce sont prioritairement les femmes enceintes et les enfants de moins de 5 ans qui dorment systématiquement sous moustiquaires. Certains affirmaient qu'au-delà de 5 ans, l'enfant ne court plus aucun risque par rapport au *tazomoka.* Cette croyance est fausse d'un point de vue médical.

> « *Notre enfant a toujours dormi sous moustiquaire jusqu'à ce qu'il a eu 6 ans. Au-delà de 5 ans, il n'y a plus aucun risque par rapport au tazomoka* » [Femme, 30 ans]
>
> « *J'ai toujours dormi sous moustiquaire lorsque j'étais enceinte jusqu'à ce que notre enfant a eu plus de 5 ans* » [Femme, 39 ans]

Mais le lien entre l'utilisation de ce moyen de prévention et la protection contre le paludisme n'est pas souvent établi. Il existe d'autres raisons d'utilisation des moustiquaires, évoquées par la plupart des enquêtés, qui sont liées à leur bien-être :

- La protection contre les désagréments causés par la piqûre des moustiques.
- La protection contre le vent au moment des siestes, qui se font généralement à un moment dans l'après- midi.
- La protection contre d'autres insectes dérangeants comme les mouches et les cafards également pendant les siestes.
- La protection contre le froid pendant l'hiver.

Les villageois connaissent également les consignes de lavage et d'utilisation, mais souvent de manière partielle. Et les discours montrent des écarts entre la connaissance de ces consignes et leur mise en application. Par exemple, certaines personnes font sécher la moustiquaire au soleil après l'avoir lavée, alors qu'il est indiqué de la faire sécher à l'ombre, la MID n'est pas toujours aérée avant son utilisation, certains la pendent à l'intérieur de la maison 24 h avant son utilisation au lieu de l'exposer à l'extérieur et à l'ombre ou bien la lavent à la rivière…

[16] Mois d'octobre 2012 (saison sèche).

La plupart des enquêtés savent que l'effet du produit peut diminuer si on le fait sécher au soleil. En revanche, la durée de l'efficacité des moustiquaires est méconnue par la population. Cependant, diverses pratiques en matière d'entretien et de lavage naissent à travers cette méconnaissance : dans certains cas, le lavage se fait rarement de peur que le produit ne parte, tandis que dans d'autres cas certains hommes pensent que la saleté diminue l'efficacité du produit si bien qu'ils les lavent avec de l'eau chaude…

Des désagréments liés à l'utilisation de la moustiquaire ont également été évoqués :

- Sensation d'étouffement à l'intérieur de la moustiquaire

- Grandeur des mailles

Certains se plaignent que les mailles des moustiquaires sont grandes, et que les moustiques peuvent piquer de travers une fois que l'effet du produit diminue[17].

- Raisons culturelles

Dormir à l'intérieur d'une moustiquaire renvoie à l'idée de la mort. En effet dans certaines pratiques de veillée mortuaire les défunts sont mis à l'intérieur d'une moustiquaire.

Un autre obstacle à l'utilisation des moustiquaires réside dans un tabou : les frères et sœurs n'ont pas le droit de dormir ensemble sous une même moustiquaire, à partir de l'âge de 12 à 13 ans. Deux frères ne peuvent plus non plus dormir dans le même lit à partir de 14 ans. Le problème de l'insuffisance du nombre de moustiquaires par famille a donc été posé.

- Insuffisance de MID

Une fois déchirées, les gens n'ont pas toujours la possibilité de racheter d'autres moustiquaires.

- La moustiquaire diminue la luminosité

- La MID provoque une irritation chez les enfants

- Honte d'utiliser une moustiquaire sale

Certaines enquêtées ont honte d'utiliser une moustiquaire sale qui se noircit vite au contact de la fumée pendant la cuisson des aliments à l'intérieur des maisons.

> *« Je ne mets pas la moustiquaire, quand elle est sale, parce qu'elle se noircit vite au contact de la fumée pendant la cuisson de la nourriture à l'intérieur de la maison. J'ai honte. Ici au village on vit avec ma belle famille »* [Femme, 25 ans].

[17] Pour les enquêtés, les: effets du produit diminuent lorsqu'ils observent que les insectes ne tombent plus au contact de la moustiquaire

- Exiguïté de la moustiquaire

Les formes de moustiquaires rectangulaires sont préférées par rapport aux formes arrondies, car elles sont plus larges et présentent plus d'ouverture, et donne à la fois beaucoup plus d'espace.

2.3.2 La CAID

La CAID est souvent connue sous le nom de DDT[18]. Pour la population c'est un insecticide qui tue les petits insectes mais pas spécialement les moustiques.

2.3.2.1 Désagréments causés par la CAID

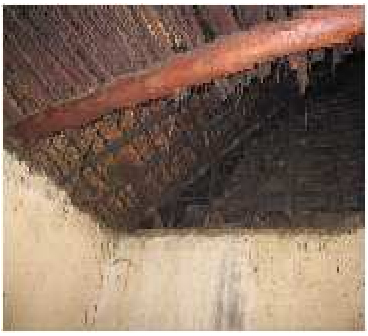

Quelques cas de refus ont été quand même été signalés par les autorités locales. En effet, les enquêtés ont exprimé de nombreux désagréments causés par le produit : il écaillerait les murs, des volailles seraient mortes suite à l'aspersion, les mauvaises odeurs persisteraient à l'intérieur des habitations après l'aspersion. Des enquêtés ont également mentionné la crainte d'être volé par les agents aspergeurs.

Type de mur, terre battue peint en fleur de Chaux. Photos D. Pourette, oct. 2012.

2.3.2.2 Perception sur l'efficacité du produit

Selon l'observation des villageois, la durée de l'efficacité du produit varie d'une semaine à un mois. Les villageois s'attendent plus à ce que le produit élimine les petites insectes comme les puces et les cafards mais pas spécialement les moustiques. Pour eux, plus le produit est clair, moins il serait efficace : les puces et les cafards réapparaitraient rapidement. Ce constat a pu faire naître une suspicion de la part des villageois vis-à-vis des agents aspergeurs, qui se livreraient à un trafic de produit.

> *« Nous constatons que le produit est clair, on ne sait plus s'ils utilisent le bon dosage. …Ils font ce qu'ils veulent, ils utilisent bien leur tête pour avoir de l'argent »* [Homme, 61 ans]

Malgré les inconvénients perçus par la population au sujet de la CAID, peu de personnes refusent les campagnes d'aspersions car elles émanent des autorités locales.

2.3.3 Le TPI

Seulement 2 femmes sur 7 ont affirmé avoir pris des médicaments contre le paludisme pendant leur grossesse. D'autres ne se souviennent plus du nom des médicaments qu'elles

[18] DDT : Dichloro diphényl trichloroétane, nom autrefois utilisé pour la campagne d'aspersion intra domiciliaire (lutte anti-vectorielle dans le cadre de la lutte contre le paludisme) dans les années 1980-90.

ont pris au cours de leur grossesse et ne savaient pas s'ils avaient un lien avec le paludisme, si bien qu'il nous a été difficile d'évaluer les connaissances en matière de TPI. De manière générale, les individus rencontrés acceptent de prendre les médicaments donnés par le médecin, même sans savoir de quoi il s'agit. Ceci pourrait s'expliquer, dans le cadre du TPI, par deux raisons. Soit les femmes ont une confiance « aveugle » envers le médecin, soit elles n'osent pas demander d'explication, craignant de ne pas être capable de comprendre les explications qui leur seront données, du fait de leur niveau d'instruction.

> *« Nous ne demandons plus ce que le médecin nous donne, il sait ce qu'il fait, nous n'avons pas poursuivi de longues études comme lui »* [Villageoise, 25 ans]

On émet également l'hypothèse que les soignants sont parfois réticents à donner des explications aux patients peu instruits, comme nous l'avons déjà mentionné.
Les ruptures de stock en TPI ont été également signalées par certains soignants comme étant la cause possible de la non-réception du traitement par les femmes. Ce qui laisse supposer qu'il y a des moments où certaines femmes enceintes ne peuvent pas en bénéficier.

2.3.4 Moyens locaux pour faire éloigner les moustiques

D'autres moyens sont mis en œuvre par les individus pour lutter contre les moustiques :

- L'utilisation des insecticides en spirale (mais peu d'enquêtés disent les utiliser en raison du coût),
- Cuisiner dans la maison car la fumée éloignerait les moustiques,
- Brûler les cyprès à l'intérieur de la maison,
- Attirer les moustiques à l'aide d'un feu, à l'extérieur de la maison, en proximité de la porte principale, puis fermer les ouvertures une fois que les moustiques s'approchent du feu.

Schéma représentatif sur les causes de la non-adoption des comportements de préventions

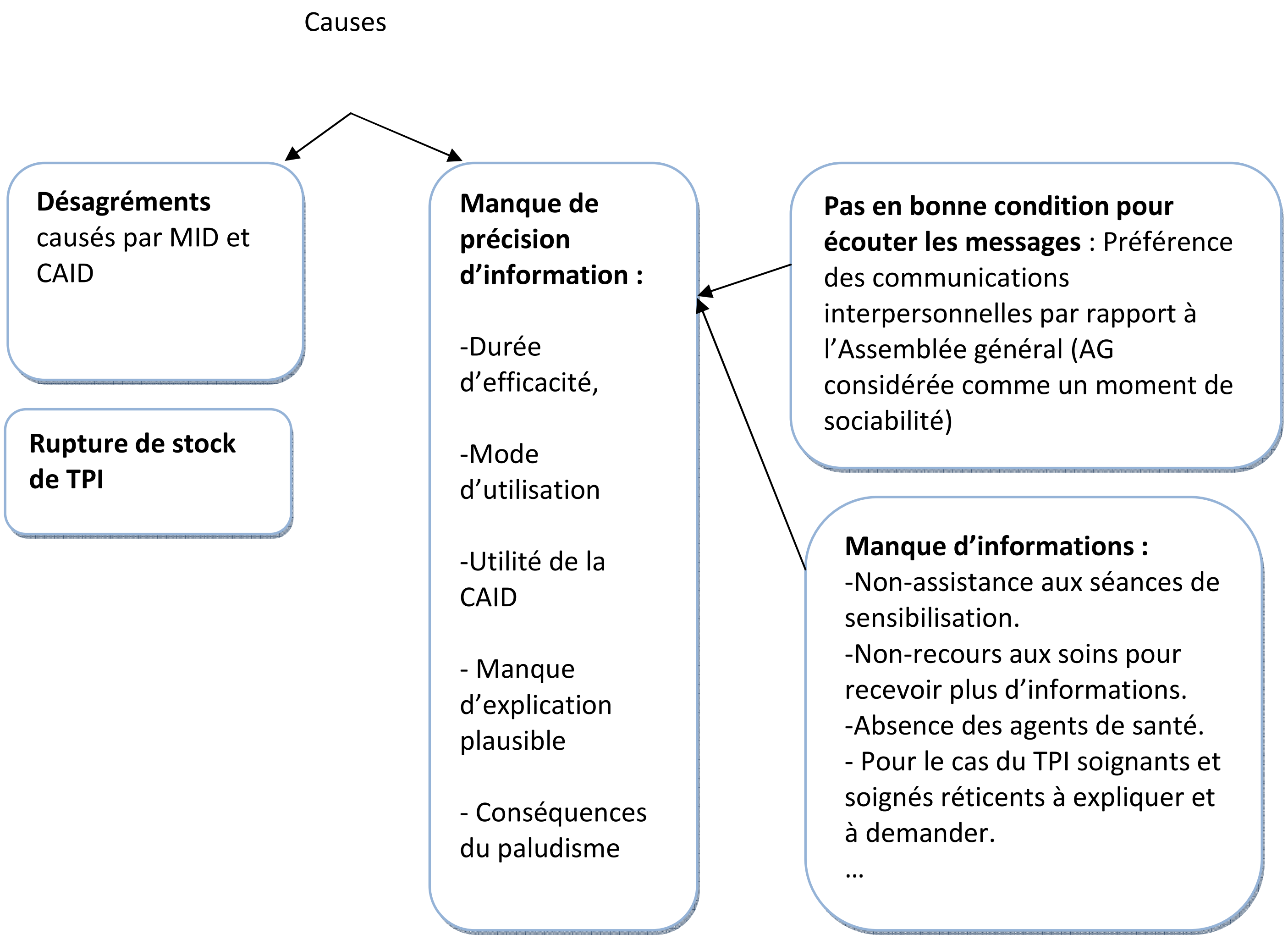

D'après nos résultats, les populations enquêtées dans cette zone ont des connaissances floues sur le paludisme, notamment en matière de reconnaissance des symptômes et de l'étiologie. Ces méconnaissances laissent naître diverses interprétations et l'adoption de comportements qui exposent les populations à des risques.

Nous avons donc constaté :

- le fait que le paludisme n'est pas perçu par la population comme un problème de santé préoccupant, et qu'il est perçu comme une maladie concernant surtout les femmes et les enfants de moins de 5 ans,
- une confusion entre les symptômes et les maladies, cette confusion étant parfois engendrée par la superposition des messages de prévention ayant des visées différentes,
- l'insuffisance d'informations et le manque de précisions dans la diffusion des messages (également liés à la superposition des messages),
- des problèmes de perceptions quant aux formations sanitaires publiques et aux agents communautaires,
- un environnement favorable à l'automédication : précarité socioéconomique, possibilité de se procurer des médicaments dans les épiceries (sans contrôle, moins chers, qui ne nécessite pas un long déplacement), facilité d'accès aux plantes médicinales, croyances concernant l'ensorcellement comme origine des maladies.

Nous avons également constaté des lacunes concernant la communication au sujet des stratégies de prévention. Les recommandations, lorsqu'elles sont suivies, ne le sont pas forcément en raison des objectifs visés (lutter contre le paludisme). Il en ressort une nécessité de mieux adapter les messages de prévention et de communication dans un souci de compréhension de la population.
Concernant les traitements prophylactiques et thérapeutiques, les ruptures de stock en TPI et en TDR ne contribuent pas à une lutte efficace contre ce problème de santé, si bien qu'ils méritent sérieusement d'être pris en compte.

II. ZONE 2 : ANTSOHIHY

Le rapport sur la zone d’étude d’Antsohihy a été rédigé par Chiarella Mattern.

TABLE DES MATIÈRES

1. CONTEXTE ET GENERALITES ... 36
1.1 Situation géographique ... 36
1.2 Caractéristiques socio-économiques ... 36
1.3 Les stratégies de lutte contre le paludisme ... 36
1.4 L'importance des ancêtres ... 37
2. NOTE METHODOLOGIQUE ... 38
2.1 Changement dans le choix de fokontany ... 38
2.2 L'entrée par l'épidémie ... 38
3. MALADIES ET ITINERAIRES THERAPEUTIQUES ... 39
3.1 Maladies considérées comme graves et fréquentes ... 39
3.1.1 Le tazo *ou la fièvre* ... 39
3.1.2 La bilharziose ... 40
3.1.3 La diarrhée ... 40
3.2 Itinéraires thérapeutiques ... 40
3.2.1 L'automédication comme premier réflexe ... 40
3.2.2 Le recours aux structures sanitaires en cas d'aggravation de l'état de santé ... 42
3.2.3 Le recours aux guérisseurs traditionnels ... 42
3.2.4 Le cas particulier de l'épidémie de paludisme de mai 2012 ... 43
3.2.5 Les femmes en charge de la santé des enfants ... 43
4. INTERPRETATION DE LA RECRUDESCENCE DE PALUDISME ... 44
4.1 Déroulement de la prise en charge des malades durant l'épidémie ... 44
4.2 Une pluralité d'interprétations : rumeurs et confusions ... 45
4.2.1 « Le tazomoka *ne tue pas »* ... 45
A. La vaccination comme cause de l'épidémie ... 46
B. « C'est la saleté qui a tué » ... 46
C. Une maladie qui vient d'en-haut ... 46
D. Autres explications ... 47
4.2.2 Une reconnaissance peu diffusée du paludisme comme cause de l'épidémie ... 47
4.3 Graphe d'interprétation de l'épidémie ... 47
5. MAIS ALORS LE PALUDISME, C'EST QUOI ? ... 49
6. PREVENTION ET TRAITEMENT CONTRE LE PALUDISME ... 50
6.1 Une priorisation au sein des ménages pour l'utilisation de la moustiquaire ... 50
6.2 Pas de dépenses opérées pour la prévention ... 51
6.3 Une utilisation intermittente de la moustiquaire ... 51
6.3.1 Les avantages de dormir sous moustiquaire ... 51
6.3.2 Les raisons d'une absence d'utilisation ... 52
6.3.3 MID versus moustiquaire du marché ... 52
6.4 Utilisation faite des MID usagées ... 52
6.5 Moustiquaires 4 portes : la « Rolls-Royce » des moustiquaires ... 53
6.6 Autres moyens de protection contre les piqûres de moustique ... 53
6.7 « La moustiquaire n'est pas suffisante » ... 53
6.8 Ressenti quant aux traitements contre le paludisme ... 54

7. CONCLUSIONS : CONFUSIONS **55**
7.1 Incompréhension face à l'apparition de symptômes nouveaux pour une maladie ancienne 55
7.2 Pluralité des traitements donnés pour une même affection 55
7.3 Quand la concurrence des messages de sensibilisation entraine la confusion 56
7.4 Complexité et inadéquation des messages de prévention contre paludisme 56
8. RECOMMANDATIONS **57**
8.1 Prise en compte des facteurs favorisant un changement de comportement 57
8.2 Repenser l'organisation des distributions et sensibilisations 57

1.1 Situation géographique

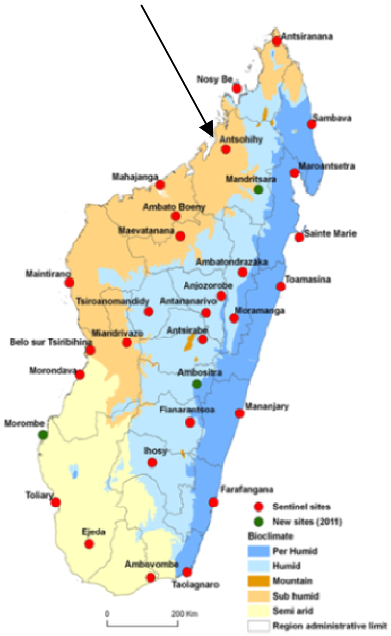

Le *fokontany* d'Ampandriakilandy se situe à 10 km d'Antsohihy, il est le chef-lieu de la commune d'Ampandriakilandy. Il est composé 2119 habitants. La commune regroupe 16 *fokontany* et est divisée en deux par un cours d'eau qui la traverse du Nord-Ouest au Sud-Est. Durant la saison des pluies, ce cours d'eau présente une frontière naturelle bloquant l'accès au 7 *fokontany* du sud, rendus accessibles par pirogue uniquement. Le Maire, un enseignant fonctionnaire à la retraite (collège) parle un français parfait et est engagé dans sa commune qu'il estime trop vaste, il lui est quasiment impossible d'être présent dans l'ensemble de la région. Son fils est agent de santé communautaire.

1.2 Caractéristiques socio-économiques

Notre *fokontany* d'intervention est accessible par une piste de 3 km difficilement praticable en saison de pluies. Il est doté d'un CSB II et se trouve à 4 km d'un centre de santé privé, tenu par des sœurs.

Les activités principales de la région sont l'élevage de zébu et l'agriculture (riz). Comme pour une grande majorité de la population malgache, l'alimentation de base est le riz. Durant la période de soudure, c'est le maïs qui est consommé. L'habitat est construit en terre battue, les maisons visitées sont décorées d'affiches, meubles, matelas, signes extérieurs d'une certaine aisance.

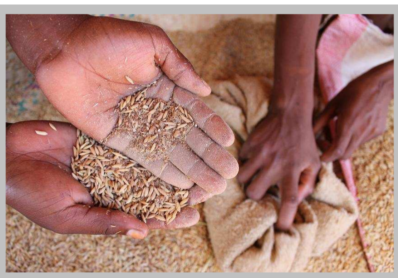

LE RIZ SOURCE DE VIE
[photo de F.Ravelomandeha]

1.3 Les stratégies de lutte contre le paludisme

La région d'Antsohihy relève du faciès tropical du paludisme. Elle est caractérisée par une méso-endémicité et une transmission moyenne et saisonnière. Les stratégies de lutte contre le paludisme dans cette région se caractérisent par la distribution de MID (de masse ou de routine) ainsi que la prise en charge des femmes enceintes : le TPI. La PCIMEc est également de mise depuis un peu plus d'un an. Jusqu'en 2010, cette région bénéficiait également de la CAID.

La région a connu une épidémie de paludisme au mois de mai 2012. Les chiffres prouvent que nous pouvons en effet ici parler d'épidémie car nous avons noté une augmentation des cas fébriles testés positivement pour le paludisme. Selon les éclaircissements de Pr. Milijaona, Chef du département Paludisme à l'Institut Pasteur de Madagascar, cette

épidémie pourrait trouver sa source dans une saison des pluies prolongée et dans un dérèglement général du système de santé dû à la crise politique et sociale qui affecte le pays: grève, rupture de stock de médicaments et de TDR, endettement du système de santé, etc.

1.4 L'importance des ancêtres

Dès que l'on passe l'entrée du *fokontany*, on a l'impression de basculer dans un autre monde, caractérisé par une grande ruralité à quelques kilomètres à peine de la ville. Un poteau sacré est érigé à l'entrée du village, appartenant à un lignage, il est le lieu où sont offertes les offrandes aux ancêtres. Durant notre passage, les villageois étaient mobilisés pour une fête de l'anniversaire de la mort d'un ancêtre. Le *fokontany* a fonctionné au ralenti pendant la durée de la procession.

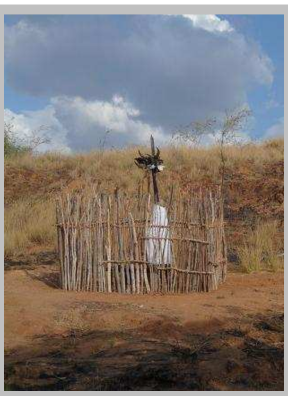

POTEAU SACRÉ

2.1 Changement dans le choix de fokontany

La méthodologie initiale a été revisitée pour des raisons liées aux conditions climatiques : en effet, Marosampanana, le *fokontany* préalablement sélectionné au cours de la mission exploratoire a été rendu inaccessible à cause de la pluie. Un nouveau *fokontany* de la même commune, Ampandriakilandy a alors été sélectionné, répondant aux critères d'accessibilité et d'intérêt pour notre étude.

En fin de séjour, les conditions climatiques nous le permettant, nous nous sommes rendus dans le *fokontany* initialement sélectionné afin d'échanger avec les villageois, se trouvant dans un contexte sanitaire très différent du *fokontany* de remplacement : la formation sanitaire la plus proche du village (l'hôpital d'Antsohihy) est à 1h30 de marche. Cependant, par sa situation géographique (isolement par rapport aux structures de soin mais accès facile en voiture en cas d'absence de pluies) ce *fokontany* est le théâtre de passages fréquents d'ONG lui conférant un rôle d'experimentateur, exposé successivement à différents projets. En conséquence, nombreux sont les messages de sensibilisation qui y aboutissent et grande est la confusion par rapport au contenu des messages. Contrairement à Ampandriakilandy où l'éventail diversifié d'interprétations quant à la maladie exprime un libre droit de penser et de s'exprimer, à Marosampanana l'agent de santé communautaire a suivi notre trace afin de s'assurer du contenu des discours tenus par nos interlocuteurs.

2.2 L'entrée par l'épidémie

La région investiguée ici est particulière de par l'épidémie de paludisme qui l'a frappée en milieu d'année 2012. Nous sommes rentrés en conversation avec les habitants par le biais de faits partagés sur le thème de l'épidémie, en tentant d'en identifier les causes perçues, les moyens de se protéger mis en place ainsi que les pratiques de soin associées à celle-ci. Rapidement, il nous est apparu que le lien était majoritairement inexistant dans le discours des interlocuteurs entre la maladie et le moustique, ce qui nous a donc conduits à nous interroger sur l'idée que nos interlocuteurs avaient du paludisme. Le canevas d'entretien initialement conçu a donc été largement modifié au profit de questions plus adéquates, au vu du contexte particulier de la zone.

La première thématique abordée dans notre canevas d'entretien concerne la santé en général et plus précisément les maladies préoccupantes en termes de fréquence et de gravité pour les interlocuteurs. L'objectif ici était de déterminer si le paludisme apparaissait comme une préoccupation dans la région investiguée. Nous avons ensuite tenté d'identifier les itinéraires thérapeutiques empruntés en fonction des maladies citées.

3.1 Maladies considérées comme graves et fréquentes

De manière unanime, l'ensemble de nos interlocuteurs a cité la fièvre ou le *tazo* comme étant une maladie grave dans le *fokontany*. En second lieu, c'est la bilharziose qui a été citée et enfin la diarrhée. La notion de « gravité » d'une maladie renvoie selon les dires des interlocuteurs à une impossibilité de la guérir par les voies habituellement empruntées en cas de maladie : l'automédication et l'attente de voir les symptômes disparaitre. Les maladies graves sont donc celles qui tuent quand on tarde à se référer à une structure de santé. L'idée également de récurrence de la maladie apparait comme une autre nuance pour la notion de gravité.

3.1.1 Le tazo *ou la fièvre*

Dans ce cadre, c'est le *tazo* qui préoccupe davantage nos interlocuteurs. Plus précisément, c'est la fièvre apparue en mai 2012 qui a créé une grande inquiétude parmi la population interrogée. En effet, plus fulgurante que la fièvre communément connue, elle n'offrait que quelques heures de délais avant de laisser apparaitre des signes graves tels que la perte de conscience et les vomissements. Peu habitués à référer les malades directement au centre de santé, nombreux sont les patients qui ont succombé à cette fièvre.

> « *C'est difficile que le tazo quitte complètement le corps. Si tu tardes à aller chez le docteur, si tu attends encore une journée, deux jours, voire trois, tu meurs* » [Femme, 55 ans][19].

Cependant, comme discuté de manière approfondie plus loin, peu de personnes ont établi le lien entre le vecteur moustique et l'épidémie. Par ailleurs, à la question de savoir quelles étaient les maladies graves et fréquentes dans la région, peu de personnes ont parlé du paludisme, comme présentant un problème de santé majeur. En effet, sur l'ensemble des personnes interrogées, un soignant seulement a nommé le paludisme comme maladie grave.

> *« La maladie la plus grave en termes de mortalité est le paludisme pendant la saison des pluies. La difficulté liée à cette maladie réside dans le fait que les symptômes sont peu marqués. On a mal le matin, on a de la température l'après-midi puis on attend encore deux ou trois jours avant de venir. La maladie n'est donc pas traitée au stade précoce »* [Soignant, 35 ans].

[19] Les interlocuteurs dont le nom de *fokontany* n'est pas indiqué sont par défaut des habitants du *fokontany* d'Ampandriakilandy.

Selon le médecin du CSB II d'Ampandriakilandy, en moyenne annuelle 70% des consultations sont pour des suspicions de paludisme. Parmi ceux-ci, 90% sont des enfants. Le médecin a évalué une moyenne de 200 consultations par mois pour son CSB, avec un pic de consultation allant jusqu'à 800 pendant l'épidémie.

3.1.2 La bilharziose

La bilharziose inquiète également, plus particulièrement la forme urinaire. Elle semble toucher majoritairement les adultes et l'étiologie est toujours liée au contact avec l'eau : ce sont surtout les hommes qui pêchent dans l'étang ou les enfants qui y jouent qui sont atteints. La consommation d'eau puisée dans les puits creusés à proximité de cet étang a également été citée comme mode de transmission de la maladie.

Cependant, si selon nos interlocuteurs cette maladie inquiète par les souffrances qu'elle provoque, le malade n'atteint jamais le stade avancé car les symptômes sont clairs : évacuation douloureuse de l'urine et sang dans l'urine. La référence au centre de santé se fait au moment de l'apparition de ces symptômes et est donc précoce.

> *« La bilharziose c'est différent du paludisme, en terme de mortalité c'est moins important car la consultation est fréquente et précoce car les symptômes liés à cette maladie sont clairs »* [Soignant, 35 ans].

3.1.3 La diarrhée

Enfin, la diarrhée a également été citée comme un problème récurrent. Sa fréquence a cependant diminué depuis l'installation d'une pompe en novembre 2010. Auparavant, les villageois s'approvisionnaient en eau dans des puits traditionnels qui, creusés trop près des étangs où s'abreuve le bétail et donc probablement trop peu profonds, entrainaient des diarrhées récurrentes. Selon les soignants interrogés, les maladies diarrhéiques restent cependant un problème, surtout en saison sèche.

3.2 Itinéraires thérapeutiques

Les itinéraires thérapeutiques fonctionnent souvent par tâtonnement et les informations circulent par les canaux de communication oraux et officieux. Si un médicament a soigné une maladie, de bouche à oreille l'idée circulera que c'est un bon médicament. Si au contraire un médicament n'a pas donné d'effets satisfaisants, une nouvelle rumeur circulera. Il en est de même pour le recours aux structures sanitaires et pour la confiance accordée au médecin, si les malades en sortent guéris et satisfaits du traitement, il bénéficiera d'une plus grande audience. Les décisions concernant le parcours thérapeutique des enfants sont en général à la charge des mères.

3.2.1 L'automédication comme premier réflexe

La première étape du recours au soin pour les malades est l'automédication. L'épicerie du village contient un minimum de médicaments pour les affections récurrentes (fièvres, paludisme, maux de ventre, etc.). Le malade charge quelqu'un d'aller à la pharmacie d'Antsohihy avec la description des maux dont il souffre, l'épicier interprète alors ces

symptômes en fonction des connaissances qu'il a acquises et propose un médicament. Un autre cas de figure peut survenir si un malade trouve des symptômes communs avec son voisin guéri suite à la prise de tel ou tel médicament.

> *« Ce sont les ragots du voisinage qui déterminent quel médicament prendre pour l'automédication. Par exemple, si lui il a ramené l'enfant chez le docteur et qu'il a guéri, il va raconter à ses voisins que l'enfant va mieux grâce à tel médicament, donc les autres vont faire la même chose si les mêmes symptômes apparaissent »* [Soignant, 30 ans].

Cette étape peut s'accompagner d'une automédication à base de plantes ou d'herbes en tisanes ou en inhalations. Pour le *fokontany* analysé, doté d'un CSB II, le recours non immédiat aux formations sanitaires trouve sa source dans deux raisons : raisons financières et peur des traitements proposés par la formation sanitaire. Plus particulièrement, la peur des injections a souvent été évoquée, pour les adultes comme pour les enfants et on préfère les éviter. Et plus précisément ce sont les effets secondaires ressentis qui ont été cités : risques de complications suite à l'injection (infection de la plaie, etc.) et sensation de « faiblesse » ressentie.

L'hypothèse des obstacles financiers est appuyée par l'agent de santé communautaire qui nous explique que le recours aux formations sanitaires augmentera pendant la période de récolte de riz quand les villageois possèdent une certaine liquidité. L'on observera au contraire une diminution de la fréquentation du CSB pendant la période de soudure car l'argent vient à manquer[20].

La question du recours au soin se pose de la même manière pour un village davantage reculé, Marosampanana, où la structure sanitaire la plus proche se trouve à 1h30 de marche : automédication en premier lieu et recours à l'hôpital uniquement en cas d'urgence.

> *« Ma femme et moi sommes tombés malade une nuit. Le lendemain j'ai chargé un voisin qui s'en allait pour Antsohihy de m'acheter des médicaments. Une fois arrivé, il a décrit mes symptômes à la pharmacie, ils lui ont donné du paracétamol et de la* Nivaquine®*. Mais au bout de 4 jours, la fièvre ne passait pas. Nous avons donc été amenés dans une charrette jusqu'à l'hôpital car nous ne pouvions plus marcher. Là-bas on m'a mis sous perfusion et on m'a donné des piqûres dans les cuisses, ma femme en a reçu sur les fesses. Nous essayons toujours d'éviter l'hôpital car ça coûte trop cher ! »* [Raiamandreny, 75 ans, Marosampanana].

Selon les estimations de cet interlocuteur, « *30% des personnes malades vont prioriser les soins traditionnels avant d'aller à l'hôpital et n'iront que s'ils sont gravement malades. 10% seulement vont directement à l'hôpital sans faire d'automédication* ».

Ainsi, que l'on dispose d'un CSB II à portée de main ou bien que l'on se trouve isolé des structures sanitaires, le premier réflexe semble être le même : l'automédication. Pour le premier cas, le recours à la formation sanitaire intervient en second lieu, mais pour le *fokontany* davantage isolé, c'est le recours aux soins traditionnels qui prend le relais.

[20] Certaines personnes ont émis la possibilité de consulter le docteur à crédit, et de rembourser quand l'argent a été épargné. Mais nous n'avons pas pu vérifier cette possibilité.

3.2.2 Le recours aux structures sanitaires en cas d'aggravation de l'état de santé

La consultation auprès d'un infirmier ou médecin intervient donc, dans le cas de notre *fokontany* d'Ampandriakilandy, en second choix en cas d'échec de la première voie de prise en charge. Ce recours à la médecine « moderne » peut être accompagné d'un recours simultané au guérisseur. L'un et l'autre se complètent plus qu'ils ne se font concurrence.

Dans le cas d'Ampandriakilandy, le médecin semble jouir d'une bonne réputation au sein de la communauté villageoise. Il est originaire d'Antananarivo et habite dans le *fokontany* avec sa famille depuis près de 3 ans. Concernant la prise en charge des accouchements notamment, le médecin collabore avec des matrones traditionnelles qui lui réfèrent les cas d'accouchements difficiles. Le médecin montre des signes d'intégration au *fokontany*.

En cas d'échec ou de non satisfaction du traitement proposé par le CSB, le malade se tournera alors finalement vers le guérisseur traditionnel.

3.2.3 Le recours aux guérisseurs traditionnels

> LE CAS DU BEBE MALADE
>
> Nous sommes interrompues durant notre entretien avec le guérisseur traditionnel par une femme, accompagnée de sa sœur et de sa fille. Celle-ci, âgée d'un an ne cesse de pleurer toutes les nuits et ce depuis plusieurs mois. Après avoir été à plusieurs reprises chez le docteur pour tenter d'identifier le malaise sans amélioration, elle vient voir le guérisseur. L'enfant a reçu de nombreuses piqures au CSB sans amélioration de son état de santé. Le guérisseur démarre alors le *tromba* ou manifestation de possession, il appelle son esprit afin qu'il communique avec l'esprit de l'enfant et découvrent de quoi elle souffre. Après une conversation de quelques minutes avec l'esprit et la présentation de l'enfant sur lequel il pose ses mains, il tend un sachet de plantes séchées à la mère qui lui donne 500 Ar en échange.

Plusieurs raisons nous ont été données expliquant le recours au guérisseur. La première est d'ordre financier. La consultation coûte 500 Ar, ce prix comprend l'identification du mal dont souffre le malade et le traitement. Parallèlement, la confiance a également été identifiée par nos interlocuteurs comme raison de recourir à ses soins. Le guérisseur est originaire du village, tout le monde le connait et il jouit d'une réputation qui lui vaut des séjours dans l'ensemble du pays pour guérir des malades. Le CSB est occupé par un médecin qui vient d'ailleurs et malgré la confiance qu'on lui accorde et son apparente intégration, on sait qu'il n'est là que pour une durée limitée. Il y a également l'idée que certaines maladies sont uniquement guérissables par le médecin et d'autres, uniquement guérissables par le guérisseur. Ces dernières apparaissent souvent de manière soudaine et s'aggravent rapidement, cela pourrait alors venir d'un sort jeté par un voisin. Enfin, on viendra chercher l'aide du guérisseur un cas d'échec de traitement proposé par le médecin car on pensera alors la cause de la maladie est à chercher « ailleurs » (ensorcellement).

3.2.4 Le cas particulier de l'épidémie de paludisme de mai 2012

Le retard de prise en charge des fièvres en général a été signalé par les soignants comme un problème important dans le cadre de la prise en charge du paludisme. Ces retards ont également été notés dans les itinéraires thérapeutiques des villageois rencontrés. Après les premiers signes d'apparition de la fièvre, 2 à 4 jours peuvent s'écouler avant la référence au centre de santé.

L'épidémie de mai 2012 a surpris l'ensemble de la commune et a modifié les errances thérapeutiques habituelles. Apparue en pleine période de travaux intenses précédant la récolte (récolte du riz au mois de mai), les parents passaient le plus clair de leur temps aux champs, enchainant parfois plusieurs jours consécutifs de travail. Les enfants étaient laissés aux soins des plus grands ou des personnes âgées. Les parents voyant leur enfant pris par la fièvre ne se sont pas inquiétés outre mesure et sont repartis travailler, remettant à plus tard la recherche de soin pour leur enfant ou procédant à l'automédication habituelle. Malheureusement, l'évolution de cette fièvre semblait plus rapide et n'a pas laissé le temps aux parents de réagir. Selon les observations de nos interlocuteurs et de l'équipe de l'Institut Pasteur intervenue pendant l'épidémie dans le cadre d'une évaluation de l'efficacité de différents médicaments antipaludiques, la fièvre a emporté un grand nombre de personnes sur l'ensemble de la commune, principalement des enfants. Le nombre de personnes décédées suite à l'épidémie est à ce jour inconnu car les déclarations de décès auprès des autorités administratives ne sont pas systématiquement effectuées dans les zones rurales isolées, principalement touchées. En outre, d'après les observations des villageois et des soignants, ce sont les enfants qui ont été les plus touchés dont la naissance n'a pour la plupart pas été enregistrée.

Un message de sensibilisation diffusé pendant l'épidémie par le personnel du CSB (médecin et dispensatrice de médicaments), les agents de santé communautaires ainsi que l'équipe de l'Institut Pasteur semble avoir été retenu : la nécessité du recours direct au médecin dès l'apparition des premiers signes de fièvre. Ceci présente un réel changement de comportement dans la recherche de soin. En effet, à la question de savoir comment protéger ses enfants durant l'épidémie et pour la saison de pluies à venir, la totalité des personnes interrogées ont cité le recours direct au CSB. Parallèlement, le médecin a observé que depuis la fin de l'épidémie elle n'avait plus pris en charge de cas de paludisme sévère, car la consultation est plusprécoce[21].

3.2.5 Les femmes en charge de la santé des enfants

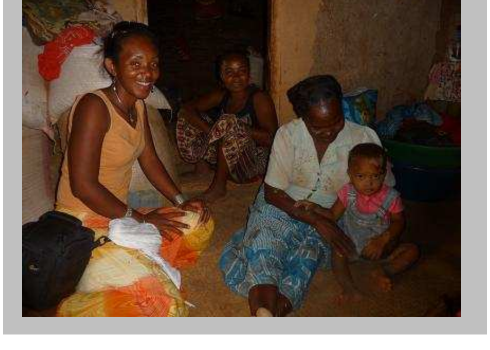

Ce qui a trait à la prise de soin de l'enfant semble être ici globalement la responsabilité de la mère. Les pratiques entourant la moustiquaire (qui dort en dessous, lavage, achat, etc.) semblent également être du ressort des femmes.

[21] Ce changement de comportement a également été noté par l'agent de santé communautaire de Marosampanana.

Les premiers cas de paludisme lié à l'épidémie sont apparus en février pour se terminer fin juin 2012. Cette épidémie et les nombreux morts qu'elle a provoqués ont marqué les mémoires des habitants de la commune d'Ampandriakilandy.

> *« Depuis que je suis consciente de la vie, je n'ai jamais vu une épidémie pareille. C'était vraiment dur. Dès le matin les gens étaient comme des fourmis devant l'hôpital et même le docteur tombait malade à cause de la fatigue car il ne dormait plus tous les jours »* [Homme, 37 ans].

> *« Au mois de mai c'était le plus grave, on avait près de 20 morts par jour dans la commune. Il y a des docteurs de Tana qui sont venus en renfort. Ce sont surtout les habitants des fokontany éloignés qui n'ont pas pu avoir recours aux soins assez vite »* [Femme, 35 ans].

> *« C'était vraiment dur car tous les 3 jours il y avait des enterrements. Il y avait des enfants qui jouaient dans la matinée et après le déjeuner mouraient déjà. Il y a des personnes qui ne croyaient pas à ce moment-là que telle ou telle personne était morte, ils disaient : mais non je viens de discuter avec lui tout à l'heure, je l'ai vu jouer ce n'est pas possible qu'il soit mort»* [Femme, 30 ans].

Absent pour cause de formation sur la PCIMEc avec les agents de santé communautaire jusqu'à mi-avril, le médecin du CSB II a pris en charge à son retour un nombre important de malades souffrant de fièvre : il a noté jusqu'à 1200 personnes pour le mois d'avril.

4.1 Déroulement de la prise en charge des malades durant l'épidémie

Dans un premier temps, le médecin et la dispensatrice de médicament ont pris en charge la première vague de malades. En cas de paludisme simple, les ATC étaient donnés aux patients. En cas de paludisme plus grave, le médecin faisait une injection intramusculaire de quinine dans la cuisse. Conscient que cela peut provoquer des abcès et que c'est interdit dans les recommandations nationales de lutte contre le paludisme, il dit avoir eu en cette période une charge trop importante de travail pour pouvoir mettre chacun sous perfusion, comme recommandé. Le paludisme grave était pris en charge par des injections de quinine.

L'équipe de l'IPM composée d'un médecin, une sage-femme et un doctorant est arrivée en renfort autour du 20 mai. Ils ont été présentés au *fokontany* par le maire au cours d'une assemblée générale, destinée à rendre transparente leur intervention et rassurer les villageois quant aux motifs de leur présence. L'équipe a alors procédé à un dépistage actif (déplacement sur le terrain afin d'atteindre les zones idées) et passif (au CSB II). Selon un soignant, « *parmi les personnes répondant positivement au TDR, 80% étaient des enfants ».*

Après l'arrivée de l'équipe IPM le 20 mai, trois traitements ont été proposés au patient souffrant d'un paludisme simple : ACT, DMF[22] et le DPT[23]. Ce dernier est un

[22] **DMF** = TPI donc normalement uniquement utilisé pour les femmes enceintes. L'IPM a voulu ici tester l'efficacité du médicament sur les adultes afin de vérifier s'il y a des résistances au médicament. 3 comprimés sont donnés en une seule prise.

médicament testé et répondait à un protocole strict : le malade devait piocher une des trois enveloppes présentées contenant le nom de leur traitement. En cas de DPT, le malade devait signer une décharge et bénéficiait, en fin de traitement, d'une somme de 3000 Ar correspondant au défraiement. Une fois le traitement terminé, une moustiquaire était offerte aux patients.

4.2 Une pluralité d'interprétations : rumeurs et confusions

Durant l'épidémie, les rumeurs allaient bon train concernant les causes de cette maladie dont les signes et symptômes étaient inconnus pour la plupart (diarrhée, vomissements, perte de conscience rapide après l'apparition des premiers signes de fièvre). Le besoin était grand alors de trouver une raison à cette recrudescence de malades. Une grande confusion règne aujourd'hui encore dans les esprits de villageois concernant l'origine de la maladie.

> *« Le docteur dit que la maladie vient des moustiques et je crois vraiment que le tazomoka existe. Mais la maladie durant l'épidémie a tué…donc ma question c'est : quelle est cette nouvelle maladie qui a tué alors que le tazomoka c'est guérissable ? »* [Femme, 24 ans].

4.2.1 « Le tazomoka *ne tue pas »*

Malgré les messages de sensibilisation de l'équipe de soignants, une majorité des villageois ne reconnait pas le moustique comme vecteur de la maladie apparue en avril-mai 2012. Si le moustique ne peut pas être le vecteur de cette maladie, il en découle que ça ne peut pas être le paludisme, tel qu'il était connu avant l'épidémie. En effet, à la question de savoir quelle était la cause de l'épidémie, la réponse communément reçue était « *D'après les docteurs c'était le tazomoka* » ou « *Je ne me souviens plus du nom de la maladie* ». En approfondissant cette réponse, il était aisé de comprendre que les explications données par les médecins ne satisfaisaient pas les interlocuteurs. La première raison avancée expliquant cette incrédulité est le faible nombre de moustiques pendant cette période d'épidémie. Le second argument avancé est que le moustique et plus précisément le paludisme n'a jamais tué. Dans ce cadre, certaines personnes ont évoqué l'idée que lors des départs en transhumance pour les pâturages des zébus, ou en période de récolte de riz, il n'est pas rare que l'éleveur ou l'agriculteur doive dormir sur son lieu de travail. Il n'est alors pas muni d'une moustiquaire, mais malgré ça, cette pratique séculaire n'a, selon les interlocuteurs, jamais entrainé la mort.

Enfin, l'observation de nouveaux symptômes ressentis de la maladie a renforcé l'idée que ça ne pouvait pas être le paludisme.

Nous ne sommes donc pas ici face à un problème de manque d'information ou de mécompréhension du message, les villageois réfutent plutôt ces messages en répondant par ces arguments.

> *« Pour moi ça ne peut pas être le tazomoka car cette maladie n'a jamais tué. Puis je ne comprends pas pourquoi cette année ce serait plus fort que les autres (…) Car*

---

[23] **DPT** = médicament testé par l'IPM.

avant on pouvait le soigner avec du paracétamol (…) Puis ensuite le fait que ça fasse si mal à la tête, c'est une raison supplémentaire pour dire que ce n'était pas le tazomoka » [Homme, 70 ans].

A. La vaccination comme cause de l'épidémie

En juin 2011, la campagne de vaccination contre le tétanos a débuté dans la région, planifiée en plusieurs phases. La dernière vague de vaccination était prévue pour le mois de janvier 2012, mais a été reportée au mois de mars pour des raisons de retard dans l'approvisionnement des vaccins. Ainsi, la dernière vague de vaccination a eu lieu juste avant le début de l'épidémie, semant le trouble parmi les parents qui ont interprété ces deux évènements avec un lien de cause à effet. Ce rapport semblait d'autant plus réel que certains enfants, suite aux vaccins, ont fait des poussées de fièvre.

Cette assimilation a eu pour effet immédiat le refus des mères de soumettre leurs enfants à la campagne de vaccination dans le cadre de la SSME (Semaine de la santé de la mère et de l'enfant), prévue dans certains *fokontany* de la commune (notamment Marosampanana).

B. « C'est la saleté qui a tué »

Les nombreux messages de sensibilisation émis durant l'épidémie ont créé une grande confusion dans les étiologies émiques[24] de la maladie. Un message par exemple consistait à dire que les moustiques se reproduisent dans la saleté (buissons, eau stagnante, déchets de tous genres) autour de la maison. En découlait la nécessité de nettoyer l'environnement de vie afin d'éviter une multiplication des moustiques. Ainsi, certaines personnes ont fait le lien direct entre la saleté et la fièvre, sans tenir compte du vecteur moustique.

> *« Peut-être ce sont les aliments ou la saleté qui ont été la cause de l'épidémie. Afin d'éviter d'attraper la maladie, il fallait être propre (soi-même, la maison, les aliments consommés) et manger sainement, se laver les mains »* [Femme, 24 ans].

Cette confusion tient également au fait qu'en peu de temps et sur un territoire délimité, les *fokontany* sont exposés à de nombreux message d'ONG et agences différentes (Sahi, Unicef, Fondation Aga Khan, etc.) offrant chacune un message lié à leurs projets spécifiques : microcrédit, eau et assainissement, hygiène des mains, etc.

C. Une maladie qui vient d'en-haut

Le côté fulgurant de la maladie a suscité nombre d'incompréhensions parmi les enquêtés et a rappelé davantage un empoisonnement ou un ensorcellement que les fièvres avec lesquelles ils sont familiers, évoluant plus lentement.

> *« Durant l'épidémie, un homme amène une dépouille mortelle dans un fokontany voisin. Le matin il est en forme mais en chemin, il se sent mal et doit être hospitalisé car il se sent très mal. De retour au village une fois guéri, tout le monde lui dit qu'il a reçu quelque chose de quelqu'un car son état a évolué trop rapidement »* [Raiamandreny, 75 ans, Marosampanana].

Une autre explication renvoie davantage à l'idée de punition, de malédiction envoyée par les esprits propriétaires des lieux sacrés qui se manifestent suite à la souillure d'un lieu sacré

[24] Dont le point de vue est basé sur les concepts et le système de pensée propre aux personnes étudiées.

par un villageois. Les villageois portaient alors des amulettes pour se protéger contre cette malédiction.

> *« L'épidémie c'était une maladie qui tombe parce que Dieu a vu parmi les villageois quelqu'un qui n'a pas respecté un fady donc il a envoyé la maladie »* [Femme, 55 ans].

D. Autres explications

L'Agent de santé nous a rapporté que la rumeur courait que cette épidémie était due à la surpopulation (non seulement à Antsohihy mais dans l'ensemble du pays) et à la nécessité de retrouver un équilibre démographique. Enfin, Le guérisseur traditionnel avait interprété la mort de nombreux enfants comme la fièvre aviaire, touchant elle aussi particulièrement les jeunes poussins. Pour se protéger, il avait alors conseillé aux villageois de dessiner sur leur front un rond de couleur, c'est ainsi qu'ils ont l'habitude de protéger les poussins contre cette fièvre. Le maire du village ainsi que l'équipe de l'IPM intervenue à ce moment-là, raconte qu'à cette période une foule de personne arboraient ces couleurs protectrices. Quelques interlocuteurs ont cité les changements de températures (élevée la journée, faible la nuit) comme source de la maladie qui les a touchés en cette période.

> *« L'épidémie n'a pas de nom mais dépend de l'arrivée de la pluie. Car il y a un changement de température entre le jour (très chaud) et la nuit (froid). Donc surtout les enfants sont touchés car ils n'ont pas le réflexe de se recouvrir d'une couverture qui pourrait être tombée, adulte si. Donc enfants tombent malades »* [Raiamandreny, 75 ans, Marosampanana].

Enfin, un soignant nous a fait part d'une conversation échangée avec un soignant de l'hôpital d'Antsohihy, durant laquelle il aurait émis une inquiétude quant à l'identification du paludisme qui pour lui s'apparentait davantage à une épidémie de méningite. Les investigations menées dans la zone par l'Institut Pasteur de Madagascar ont confirmé qu'il s'agissait bien de paludisme.

4.2.2 Une reconnaissance peu diffusée du paludisme comme cause de l'épidémie

Une minorité de personnes interrogées a reconnu le lien entre le moustique et la maladie. Parmi ces individus, une partie a reconnu que cette maladie étant le paludisme, d'autres l'interprétaient davantage comme une nouvelle maladie, transmise par le moustique. Ces derniers se basaient sur l'observation que l'épidémie a touché particulièrement les jeunes enfants, qui sont les plus exposés aux piqûres de moustiques. A titre d'exemple illustrant cette seconde catégorie de personnes, un homme a interprété cette nouvelle maladie comme étant la drépanocytose, suite à une émission radiophonique dans laquelle il aurait entendu que cette dernière était une nouvelle maladie transmissible par le moustique.

4.3 Graphe d'interprétation de l'épidémie

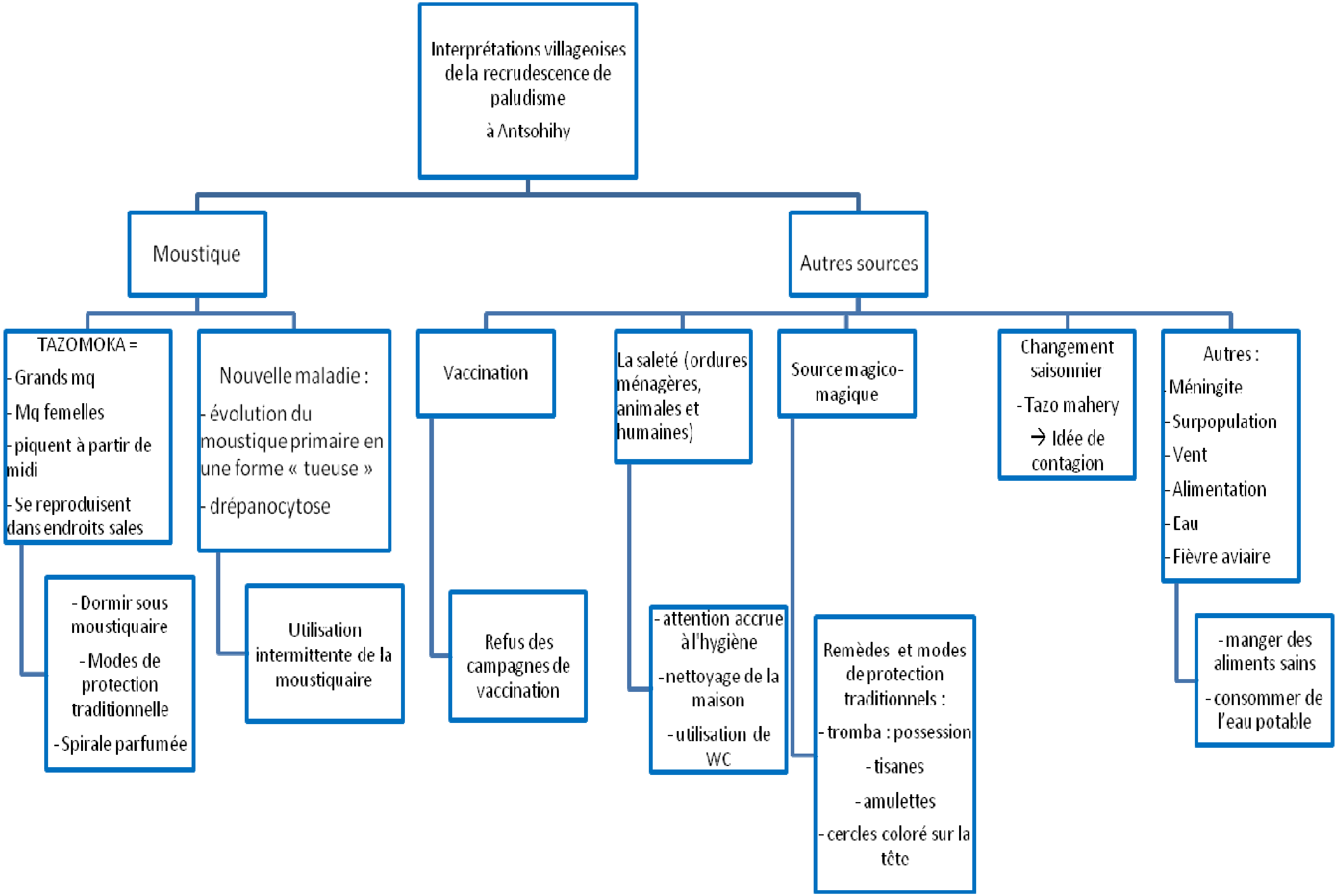
Interprétations villageoises de la recrudescence de paludisme
à Antsohihy
Moustique
Autres sources
TAZOMOKA =
- Grands mq
- Mq femelles
- piquent à partir de midi
- Se reproduisent dans endroits sales
Nouvelle maladie :
- évolution du moustique primaire en une forme « tueuse »
- drépanocytose
Vaccination
La saleté (ordures ménagères, animales et humaines)
Source magico-magique
Changement saisonnier
- Tazo mahery
→ Idée de contagion
Autres :
- Méningite
- Surpopulation
- Vent
- Alimentation
- Eau
- Fièvre aviaire
- Dormir sous moustiquaire
- Modes de protection traditionnelle
- Spirale parfumée
Utilisation intermittente de la moustiquaire
Refus des campagnes de vaccination
- attention accrue à l'hygiène
- nettoyage de la maison
- utilisation de WC
Remèdes et modes de protection traditionnels :
- tromba : possession
- tisanes
- amulettes
- cercles coloré sur la tête
- manger des aliments sains
- consommer de l'eau potable

Ces différentes interprétations de la maladie nous ont alors conduits à nous demander comment les villageois perçoivent le paludisme, tel qu'il apparaissait avant l'épidémie : symptômes et parcours de recherche de soin, traitements adéquats, interprétation des sources et origines de la maladie. Cette apparente rareté de paludisme dans la région avant l'épidémie trouve sa source dans un fait très concret : une personne malade connait rarement le nom des maladies dont elle souffre. Comme nous l'avons vu plus haut, le premier réflexe en cas de fièvre était l'automédication. Les échanges entre le malade et l'épicier se résument à la description des symptômes, suivis par l'achat de paracétamol et de Paludar® (chloroquine)/Palustop® (sulfadoxine-pyriméthamine). Aucun diagnostic n'est donc émis, le patient ne faisant pas le lien entre le médicament pris et le paludisme. En second lieu, si l'automédication a échoué, il ira voir le médecin. Si celui-ci diagnostique le paludisme, il lui prescrira le traitement, mais ne lui donnera souvent ni le nom du médicament, ni le nom de la maladie dont il souffre. Il se contentera de décrire la posologie. La raison évoquée par un soignant pour ce manque de transmission d'information est le faible niveau de scolarité de la majorité des patients, qui ne lui permettrait pas de lire, ni de comprendre des explications trop complexes. Ce manque d'information a été confirmé durant les entretiens avec la population qui dans aucun cas n'a pu donner le nom du médicament reçu suite à des fièvres.

Ainsi le paludisme est nommé souvent par le terme fièvre forte ou *tazo mahery,* du nom du symptôme de « corps chaud » ressenti.

> *« Il y a une différence entre le tazo et le tazo mahery. La première fièvre pourrait s'apparenter à une fièvre mais peu forte, allant jusqu'à 38°. La seconde fièvre est très forte. C'est à celle-là qu'on associe le paludisme »* [Soignant, 30 ans].

Le traitement adéquat est identifié par l'épicier ou le médecin mais rarement communiqué au malade[25]. Ce n'est pas, pour les interlocuteurs, une maladie pouvant entrainer la mort. La fièvre et le paludisme sont fortement assimilés. Selon les dires des interlocuteurs, seul le médecin peut déterminer si c'est l'un ou l'autre. Quoiqu'il en soit, le paludisme n'est pas perçu comme une maladie grave ou préoccupante par la population.

[25] Il semblerait que les malades rencontrés portent peu d'intérêt à mettre un nom sur leur maladie.

Comme on peut aisément l'imaginer, les comportements de prévention sont largement liés aux représentations et interprétations de la maladie. Ainsi, les personnes ne reconnaissant pas le lien entre l'épidémie et le moustique ne changeront pas leurs comportements habituels concernant la moustiquaire dans un objectif de prévention contre la maladie : ils continueront à l'utiliser sporadiquement en fonction de différents facteurs approfondis ci-dessous. Les moustiquaires utilisées sont majoritairement des moustiquaires 4 portes et non des MID (raisons évoquées : préférence de la première et faible distribution de la seconde).

Pour ceux qui les utilisent, elles restent suspendues toute l'année mais elle sera descendue et remontée en fonction des besoins. L'observation de la présence d'une moustiquaire attachée n'est donc pas un indicateur de l'utilisation réelle de celles-ci.

Les messages de sensibilisation diffusés autour de la MID sont connus et peuvent aisément être restitués par les interlocuteurs, notamment l'importance de dormir sous moustiquaire, surtout pour les femmes enceintes et pour les nourrissons. La priorité sera donc donnée à la femme enceinte, au détriment du reste de la famille. Les messages concernant son entretien ont également été retenus : aération de la MID avant utilisation, lavage dans une cuvette avec savon en bloc, séchage à l'ombre.

6.1 Une priorisation au sein des ménages pour l'utilisation de la moustiquaire

Nombre de personnes ont indiqué un manque de moustiquaires pour le ménage, entrainant une plus grande vulnérabilité des membres de la famille ne dormant pas sous moustiquaire. L'on a observé que si un ménage dispose de trop peu de moustiquaires, la mère enceinte ou avec un jeune enfant en bénéficiera en premier, le couple père/mère ensuite et en dernier lieu les enfants de plus de 3 ans.

> *« C'est moi qui dort sous moustiquaire, même si j'ai trois enfants dont le dernier a 6 ans. Parce que les enfants sont déjà grands ils ne dorment pas sous moustiquaires et qu'on m'a dit à l'hôpital que c'était les femmes enceintes qui devaient dormir sous moustiquaire »* [Jeune femme, 26 ans].

La possession de moustiquaires est cependant variée : certains ménages en ont trois en réserve dans l'armoire, d'autres en sont à court. Au vu du nombre de distributions réalisées ces dernières années, il est difficilement compréhensible qu'un ménage n'en dispose pas[26].

[26] En 2009, les distributions de MID de « routine » étaient en place (distribution de MID pendant les CPN pour femmes enceintes et allaitantes). La CAID était également encore d'actualité. En 2010, il y a eu une distribution de masse (offrant deux moustiquaires/ménage), la CAID a été mise réalisée pour la dernière fois dans la région. En 2011, les distributions de « routine » ont été réalisées.

6.2 Pas de dépenses opérées pour la prévention

L'on a observé également au sein des ménages disposant de moustiquaires en nombre insuffisant que rares sont les dépenses émises dans le cadre de la prévention contre le moustique. Le prix d'une moustiquaire est de 5000 Ar sur le marché, celui d'une moustiquaire décorative dotée de dentelles et de rubans de couleurs peut varier de 20 000 Ar (confectionnée par une couturière) jusqu'à 150 000 Ar (moustiquaire 4 portes[27] déjà cousue).

6.3 Une utilisation intermittente de la moustiquaire

L'utilisation de la moustiquaire dépend de plusieurs facteurs. Le premier est la disponibilité gratuite des moustiquaires. Le second est le degré de conscientisation de la mère, quant aux maladies transmissibles par le moustique. Ensuite, cela dépendra de la saison et de la présence ou non de moustiques.

6.3.1 Les avantages de dormir sous moustiquaire

- **Côté pratique**

Le côté pratique de l'utilisation d'une moustiquaire a été mis en évidence par une majorité des interlocuteurs comme première raison de l'utilisation de la moustiquaire : elle protège de la chute d'insectes dans le lit, de la gêne des moustiques (bruits et piqûres). C'est la raison la plus souvent évoquée légitimant l'utilisation de la moustiquaire.

- **Côté confort**

La moustiquaire procure également un sentiment de confort, elle crée pour les couples un espace d'intimité dans un contexte de cohabitation souvent exigüe avec de nombreux enfants. Parallèlement, elle garde la chaleur en hiver et protège du vent.

> *« La moustiquaire permet de se protéger du froid. Car même si les enfants font tomber leur drap en hiver au moins ils continuent à avoir chaud car dans la moustiquaire il fait plus chaud. Ça leur évite d'attraper froid »* [Femme, 55 ans].

Les personnes plus âgées nous ont raconté que lorsqu'ils étaient petits, leurs parents dormaient déjà sous moustiquaires. A cette époque, il semblerait qu'elle était essentiellement réservée aux adultes. Un soignant a évoqué l'idée qu'à l'époque coloniale, les moustiquaires étaient perçues comme un objet de haut standing, signe de richesse et donc apprécié par la population.

- **Côté préventif**

Enfin, quelques interlocuteurs ont également évoqué la protection contre le paludisme.

[27] Ces moustiquaires sont caractérisées pour une ouverture sur chaque versant de la moustiquaire, ce qui facilite l'entrée et la sortie. Elles sont fréquemment décorées de dentelles de couleurs et ne sont pas imprégnées.

6.3.2 *Les raisons d'une absence d'utilisation*

Les raisons évoquées pour la non-utilisation des moustiquaires sont multiples. Certaines personnes mettent en évidence la chaleur et la difficulté de respirer sous une moustiquaire en saison chaude ou en cas de maladie (la température est élevée, besoin d'air et donc impossible de dormir sous moustiquaire). Les périodes de grande chaleur correspondent aux périodes où les moustiques sont nombreux.

L'idée que le moustique ne tue pas a été évoquée également comme raison de non utilisation de la moustiquaire. Comme le paludisme n'est pas considéré comme une maladie grave, il n'est pas nécessaire de surmonter les désavantages liés au fait de dormir sous celle-ci. Certains ont évoqué également la présence de moustiques dès midi. Ces personnes ne voient pas l'utilité de dormir sous une moustiquaire puisqu'elles sont de toute façon en contact avec les moustiques dès l'après-midi. Enfin, certains affirment que globalement, il y a peu de moustiques dans la région. Ils n'ont donc pas besoin de se protéger.

6.3.3 *MID versus moustiquaire du marché*

Selon les interlocuteurs, l'avantage de la MID est qu'elle est imprégnée. La présence du produit est connue des villageois. Elle ne permet donc pas uniquement de se protéger contre les moustiques une fois à l'intérieur de la moustiquaire, elle entraine également l'élimination des insectes, une fois posés sur le tissu imprégné. Un villageois nous a dit être dérangé par l'odeur forte de la MID, les autres considéraient l'imprégnation comme un grand avantage.

Le défaut qui a été souligné est le fait que les MID sont trop courtes. Un soignant nous a expliqué qu'il faisait systématiquement coudre une bande de tissus dans le bas des moustiquaires posées au-dessus des lits de son centre de santé afin de les border sous le matelas, comme prévu dans les recommandations. Le sentiment d'étouffement lié à une moustiquaire trop courte a souvent été mis en évidence.

6.4 Utilisation faite des MID usagées

De nombreuses moustiquaires usagées sont accrochées sur les clôtures, dans les arbres, çà et là dans le village. Rougies par la terre et trouées, elles sont utilisées pour la pêche ainsi que pour la protection contre les poules du riz sur pied ou récolté et stocké dans les cours des maisons. Les villageois affirment que ce sont d'anciennes moustiquaires usagées, elles ont donc selon eux été remplacées par de nouvelles moustiquaires dans les habitations. Selon eux toujours, les nombreuses distributions de moustiquaires de cette année offrent l'opportunité de remplacer assez rapidement une moustiquaire usagée, afin de l'utiliser à d'autres fins.

Certaines femmes ont également évoqué l'accumulation de moustiquaires inutilisées et soigneusement rangées dans les étagères. Celles-ci préfèrent les garder en cas de besoin,

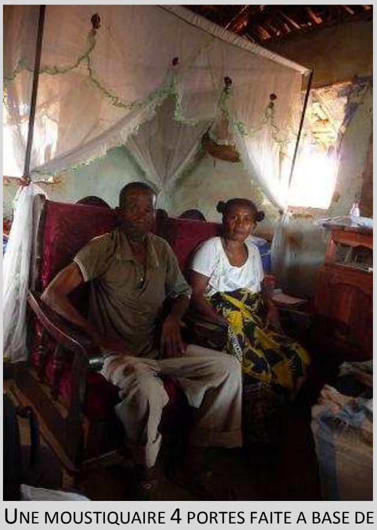

UNE MOUSTIQUAIRE 4 PORTES FAITE A BASE DE MID

plutôt que de les vendre ou les donner à des ménages voisins dans le besoin. Parmi elles, une femme nous a expliqué qu'à l'aide de trois MID elle s'était fait coudre une moustiquaire à 4 portes, considérées comme une moustiquaire de luxe.

6.5 Moustiquaires 4 portes : la « Rolls-Royce » des moustiquaires

A la question de savoir : « quels aménagements voudriez-vous pour votre moustiquaire imprégnée ? », les femmes nous ont en quasi-totalité répondu qu'elles préféraient largement la « moustiquaire 4 porte » vendue au marché à un prix élevé (entre 20 000 et 100 000 Ar). La raison avancée est le côté « à la mode » de ces moustiquaires, dotées de dentelles et de galons de couleur. Le côté pratique a également été avancé : elles ont des portes sur chaque pan, ce qui facilite l'entrée et la sortie tout en évitant l'entrée des moustiques. Etant un objet de grande taille, prenant souvent une place considérable au sein d'habitations modestes (en moyenne 9 m²), les femmes jugent important que l'objet soit joli et elles accordent de l'importance également à la propreté de celle-ci. Pour ce qui est de la couleur, le blanc peut-être maintenu, aucune femme n'a évoqué un problème quant à celle-ci.

6.6 Autres moyens de protection contre les piqûres de moustique

Les interlocuteurs ont cité des modes de prévention traditionnels contre les attaques de moustiques : bruler des bouses de zébu devant l'entrée de la maison ou des feuilles de manguiers. Cela dégage une odeur forte et une fumée qui éloignent les moustiques.

Le drap de lit a été cité par certains comme étant une protection valable contre les piqûres de moustiques. Selon eux, le problème se situait alors au niveau des enfants car ils n'ont pas le réflexe de se recouvrir, une fois le drap tombé durant la nuit.

> « *Si on recevait deux moustiquaires supplémentaires, on en mettrait une dans la cuisine car il faut une personne en permanence à cause des cambriolages et une autre pour les deux enfants car comme elles sont encore petites elles ne savent pas comment se recouvrir de nouveau du draps quand il tombe. Mais les grands ils savent déjà comment se recouvrir du drap quand il tombe, il sait se protéger* » [Homme, 37 ans].

Les spirales anti-moustiques ont également été citées comme prévention prise contre les piqûres.

6.7 « La moustiquaire n'est pas suffisante »

Il a été noté qu'une utilisation systématique (ce qui est rare) de la MID toutes les nuits tout au long de l'année ne peut pas être l'unique protection mise en place pour se protéger contre le paludisme. En effet, en saison chaude, les habitants se regroupent régulièrement sous leurs vérandas avec les grands enfants pour discuter et ne rentrer sous la MID qu'aux alentours de 21h, quand la chaleur est tombée. De même, les soirs de pleine lune les

villageois profitent de la clarté de la nuit pour continuer leurs activités à l'extérieur de la maison.

6.8 Ressenti quant aux traitements contre le paludisme

Deux soignants sur les 5 rencontrés nous ont fait part des nombreux effets secondaires des ACT : maux d'estomac, perte d'appétit, fatigue ralentissant les travaux aux champs. L'un d'eux a noté l'inefficacité du traitement et a préféré revenir, du moins pour les enfants, aux anciennes recommandations : Nivaquine® (chloroquine) donnée en prophylaxie en une prise, une fois par semaine durant toute l'année, jusqu'à un certain âge, non précisé. Ce même soignant estime que les doses de TPI données aux femmes enceintes sont trop faibles et en donne 2 fois par mois, contre 2 doses prévues pour l'ensemble de la grossesse au niveau des recommandations nationales.

De nombreux interlocuteurs ayant reçu le traitement ACT, ont également mis en évidence la grande fatigue ressentie suite à la prise de médicament, ainsi que la perte d'appétit.

Au vu de la méconnaissance généralisée du nom du médicament donné généralement en cas de fièvre et pendant l'épidémie (que ce soit en épicerie ou au dépôt de médicament du CSB), il nous a été impossible de comparer les effets ressentis et les préférences des patients en termes de traitement.

7.1 Incompréhension face à l'apparition de symptômes nouveaux pour une maladie ancienne

Comme nous l'avons dit plus haut, les symptômes nouveaux apparus avec l'épidémie de paludisme en début d'année 2012 ont questionné les villageois quant à la définition de paludisme reconnue jusqu'ici d'après les symptômes suivants : fièvre forte associée à une perte d'appétit. Cette maladie ne tue pas. Elle évolue lentement et peut être guérie grâce aux traitements achetés en épicerie : paracétamol et Paludar® (chloroquine)/Palustop® (sulfadoxine-pyriméthamine). Les nouveaux symptômes (diarrhée, vomissements, perte de conscience) associés à l'épidémie sont alors interprétés comme les signes d'une nouvelle maladie.

7.2 Pluralité des traitements donnés pour une même affection

Outre l'apparition de symptômes inconnus, l'Institut Pasteur, dans le cadre de la lutte contre l'épidémie a proposé plusieurs traitements. L'équipe de l'IPM s'est présentée au cours de l'assemblée générale en précisant que durant leur présence, l'équipe prendrait exclusivement en charge le paludisme, tandis que le médecin du CSB s'occuperait des autres maladies. Donc, tous les malades pris en charge par un membre de l'équipe IPM devaient normalement souffrir de la même maladie : le paludisme. Et pourtant, lors de conversations spontanées entre villageois, les patients ont remarqué des différences de traitements donnés à l'un et à l'autre, ce qui a induit des confusions.

DISCUSSION AVEC DEUX MAÇONS

Deux maçons travaillent à la construction d'une maison. L'un d'eux a contracté le paludisme pendant l'épidémie et s'est retrouvé dans la file de dépistage au mois de mai. Son collègue maçon accompagnait sa petite fille, venue également pour un dépistage. Une fois testés positivement pour le paludisme tous les deux, le premier est confronté à un choix posé par un soignant : ou bien il prend 3 comprimés à avaler (le premier est pris sous supervision du docteur, les deux suivants sont pris à domicile) ou bien il prend un autre traitement qui exige de nombreux aller-retour au CSB. Il opte donc pour le premier, qu'il estime moins contraignant. Son collègue, lui se voit imposé le second traitement et revient toutes les semaines à 4 reprises avec sa fille pour des prises de sang. Une grande confusion s'installe donc dans les esprits des deux hommes : l'un dit, voyant que les symptômes étant les mêmes et étant tous les deux pris en charge par l'équipe de l'IPM, qu'ils doivent forcément souffrir de la même maladie. L'autre, est catégorique, c'est peut-être le *tazomoka* dont ils souffraient tous les deux, mais des paludismes de type différents.

Ceci a entrainé une grande confusion et de nouvelles rumeurs. Une femme explique la différence de traitement par le stade de gravité dans lequel se trouve le malade : l'ATC pouvant être pris à domicile et n'exigeant que trois prises d'un même comprimé serait le traitement réservé aux malades dans le premier stade de la maladie, tandis que le traitement testé, plus lourd et plus contraignant (il exigeait de nombreux allers – retours au

CSB), serait réservé aux malades en état grave. C'est en raison de la lourdeur du traitement que les patients étaient défrayés, toujours selon nos interlocuteurs.

7.3 Quand la concurrence des messages de sensibilisation entraine la confusion

Le nombre important de messages transmis par les ONG, associations ou autres entraine une confusion généralisée parmi les villageois. Un message en remplace un autre, une affiche en remplace une autre, certains acteurs de développement n'hésitent pas à utiliser l'épidémie pour ancrer leurs messages. Ainsi par exemple, de passage pendant l'épidémie à Marosampanana, une ONG travaillant dans le domaine de l'hygiène et l'assainissement a profité du terrain propice de l'épidémie pour dire qu'il faut construire des toilettes et se laver les mains pour éviter de tomber malade. Il y a donc une réelle concurrence des messages des structures de développement entrainant la confusion entre les différentes problématiques.

7.4 Complexité et inadéquation des messages de prévention contre paludisme

Certains aspects des messages diffusés par les acteurs de la lutte contre le paludisme ont été reportés comme étant trop complexes par les villageois et semblent contribuer à une certaine confusion. Le fait par exemple de savoir que seuls les moustiques femelles transmettent la maladie pose question à nos interlocuteurs : « *On nous dit ça mais comment on reconnait un mâle d'une femelle ?* ».

Parallèlement, ces messages accentuant l'importance de dormir sous moustiquaire semblent pour certains d'entre eux inadaptés au quotidien des utilisateurs. En effet, la chaleur entraine les discussions dans la fraicheur de la soirée et les travaux dans les champs ne permettent pas un retour systématique pour la nuit au village.

Le nombre de MID distribuées est problématique : une moustiquaire est offerte pour 3 membres de la famille. Ce mode de distribution ne tient pas compte des *fady* que présente le partage d'un même lit pour un garçon et une fille à partir de l'âge de 10 ans.

Enfin, nombreuses sont les questions qui émergent quant à l'utilisation et la distribution de ces moustiquaires et rares sont les espaces permettant l'échange de réelles informations (versus les échanges de rumeurs et de ragots communes dans le *fokontany*) concernant les moyens de prévention et la maladie.

8.1 Prise en compte des facteurs favorisant un changement de comportement

> *« Avant si on n'avait pas d'argent, on n'allait pas à l'hôpital pour se soigner. Mais depuis l'épidémie, tout le monde a compris que quand on a de la température, il faut aller voir le médecin tout de suite. Avant quand un enfant avait de la fièvre on lui donnait un paracétamol ou des inhalations. Mais en mai quand on faisait ça, la fièvre revenait sans cesse car la maladie n'était pas partie, au contraire, ça s'aggravait »* [Femme, 55 ans].

Pendant l'épidémie de 2012, les villageois ont bien intégré le message selon lequel il fallait adresser les enfants au CSB aux premiers signes de fièvre. La réussite de la transmission du message « référence au CSB aux premiers signes de fièvre de l'enfant » est due à trois facteurs : la peur de l'épidémie et l'état de santé altéré dans lequel se trouvaient les malades, l'observation de l'efficacité de la prise en charge par le médecin et des traitements proposés et, selon certains, le fait qu'une partie des malades aient durant l'épidémie été rémunérés pour prendre leur traitement. Pour qu'un message soit assimilé, le besoin est grand de preuves tangibles et d'explications adaptées au contexte des bénéficiaires. Ainsi, concernant l'épidémie par exemple, ou dans la communication sur le paludisme plus globalement, les explications doivent être plausibles et avoir des fins réelles.

8.2 Repenser l'organisation des distributions et sensibilisations

Il est nécessaire d'adapter les systèmes de distribution de moustiquaires aux *fady* et habitudes locales. Par exemple, à partir d'un certain âge, deux frères ou deux sœurs ne peuvent plus partager le même lit. Parallèlement, de nombreuses questions restent inexpliquées au sein du *fokonolona* : pourquoi une même maladie tue un jour et rend malade un autre jour ? Est-ce le même moustique ? Comment reconnaitre un moustique femelle ? La création d'un espace de partage serait une plus-value essentielle dans le cadre des distributions de moustiquaires, où les villageois pourraient exprimer leurs désaccords (« *ok c'est le moustique femelle qui donne le tazomoka, mais en quoi cette information peut-elle nous servir à mieux nous protéger* ? » ou encore « *Pendant des années, nous sommes allés travailler aux champs, nous y sommes resté dormir sans moustiquaires sans même tomber malades, et cette année cette même pratique a tué des proches* ? ») et poser leurs questions, les confronter à des réponses qui leur paraissent plausibles. Car nombreux sont les ragots, nombreuses sont les rumeurs aussi qui conduisent à la non mise en pratique de comportements de prévention adaptés.

Parallèlement, l'analyse du cas particulier d'Antsohihy a mis en évidence les interprétations plurielles pour une même maladie. Au vu de ces particularités locales, il nous parait donc essentiel de ne pas uniformiser les messages à l'échelle du pays mais au contraire d'adapter les messages aux spécificités locales.

Les pratiques des villageois, qu'elles relèvent du domaine de la santé, de l'agriculture ou autres, fonctionnent toujours par tâtonnements et sont transmises oralement depuis des générations par les canaux de communication officieux. Les pratiques innovantes

entraineront la méfiance et la peur à cause de l'inconnu auquel elles renvoient. Dans la région d'Antsohihy, les campagnes gagneraient beaucoup à intégrer les leaders locaux (Raiamandreny, maires, instituteurs, président des différents comités, toute personne identifiée sur des critères de confiance, de proximité avec la population et non uniquement par défaut les présidents de *fokontany*) dans les campagnes. Afin que ceux-ci, par le biais de la confiance et la familiarité dont ils bénéficient, entrainent, par l'adoption de pratiques adéquates, l'ensemble du *fokonolona*, réticent à l'utilisation des moustiquaires et plus globalement, au recours aux formations sanitaires en premier recours.

III. ZONE 3 : FIANARANTSOA

Le rapport sur la zone d'étude de Fianarantsoa a été rédigé par Emma Raboanary.

Table des matieres

1. Contexte et generalites 61

1.1 Localisation géographique 61

1.2 Contexte épidémiologique du paludisme à Fianarantsoa 61

1.3 Justification du choix de Fokontany 61

2. resultats 62

2.1 Connaissances de la population sur le paludisme 62

2.1.1 Le paludisme est perçu comme un problème de santé qui ne touche pas la population 62

2.1.2 Le paludisme est perçu comme une maladie contractée sur les côtes 62

2.1.3 Connaissances partielles sur les symptômes du paludisme 63

2.1.4 Diverses interprétations sur les causes du paludisme 63

2.2 Problèmes rencontrés par les soignants en matière de prise en charge du paludisme 64

2.3 Comportements thérapeutiques en cas de fièvre non favorables à la détection rapide du paludisme. 65

2.4 Les pratiques préventives de la population **........ 69**

2.4.1 La CAID 69

2.4.1.1 Utilité perçue de la CAID : tuer les puces et les cafards 69

2.4.1.2 Désagréments causés par la CAID 69

2.4.1.3 Perception sur l'efficacité de la CAID 69

2.4.2 Le PECIMEc 70

2.4.2.1 ACT périmés 70

2.4.2.2 La sensibilisation pour la lutte contre le paludisme n'est pas une priorité selon un agent communautaire 70

2.4.3 Quelques moyens évoqués pour se protéger contre les piqûres de moustiques 70

2.4.4 « Anticorps » contre le paludisme : les petites bichiques et l'ananas 71

3. Conclusions et recommandations 72

3.1 Le paludisme n'est pas perçu comme une maladie qui touche la population 72

3.2 Prioriser la sensibilisation sur le paludisme importé 72

3.3 Importance des relations avec les tradipraticiens « officiels » 72

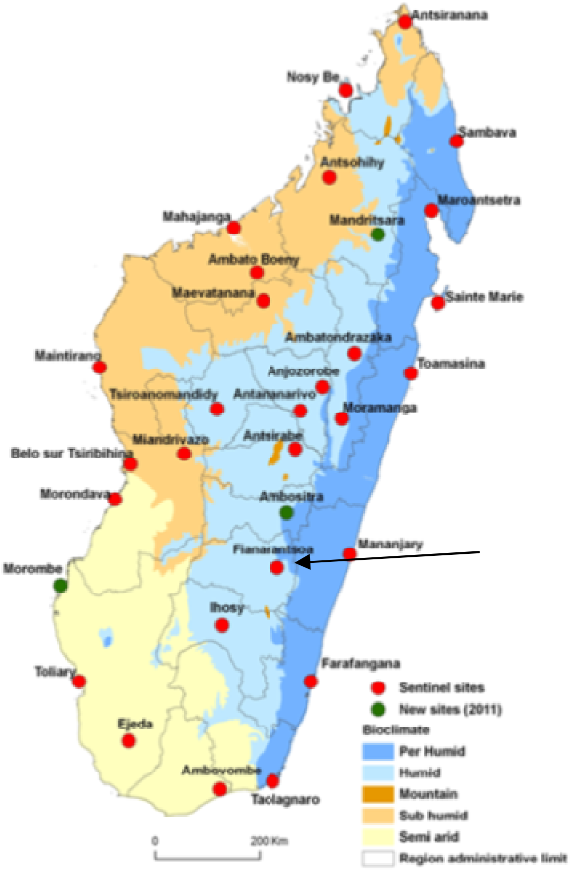

1.1 Localisation géographique

Notre enquête s'est déroulée à Sahamena, un *fokontany* de la commune rurale d'Andrainjato Centre, district de Lalangina, Fianarantsoa II, qui se trouve à environ 10km de Fianarantsoa centre et à proximité du CSBII, du 05 au 17 novembre 2012.

En termes d'organisation sociale, les travaux des villageois sont réalisés par un système d'entraide, sous contrôle des *Ray aman-dReny* ou les aînées qui détiennent un statut social important. Il existe des associations villageoises de solidarité sociale ou *fikambanan'ny tanàna*, où ces *Ray aman-dReny* cotisent plus que les autres, ils ont le pouvoir de décision sur ce qui concerne l'organisation sociale. Notons que ces derniers s'intègrent également dans le pouvoir administratif local et jouent donc un rôle important sur la vie sociale.

1.2 Contexte épidémiologique du paludisme à Fianarantsoa

Comme nous l'avons vu précédemment Fianarantsoa est une zone de transmission nulle ou très faible, contigüe au faciès à l'Est avec présence de paludisme importé. Les stratégies de lutte mises en place sont les suivantes : La CAID, le TPI, le PECIMEc et la Prise en charge.

1.3 Justification du choix de Fokontany

Sahamena est exposée au risque de paludisme importé car, selon les informations recueillies sur terrain, elle constitue une zone de passage des populations qui résident dans le corridor (zone endémique) de la côte est, et car les villageois effectuent des migrations saisonnières en zone d'endémie, généralement pendant 2 à 5 mois par an, afin de faire face à la période de soudure[28]. Toujours selon les données recueillies sur terrain, les mouvements quotidiens de la population s'effectuent à l'intérieur de la localité ou dans les localités environnantes pour effectuer les travaux agricoles (système d'entraide ou salariat en ½ journée). Les hommes ou les femmes transportent quotidiennement les produits agricoles aux divers marchés dans les villes. Tels sont les différents contextes et mouvements de la population

[28] Généralement au mois de novembre au mois de mars

utiles pour comprendre les enjeux pour faire face aux problèmes de santé et en particulier au paludisme.

2.1 Connaissances de la population sur le paludisme

2.1.1 Le paludisme est perçu comme un problème de santé qui ne touche pas la population

Les maladies fréquentes évoquées par les enquêtés étaient le *tazo* ou la fièvre, le *tazo vony* ou la jaunisse, la diarrhée, les maladies respiratoires, la grippe et les maux de dents. En revanche le paludisme est perçu comme un problème de santé qui ne touche pas souvent la population du fokontany aussi bien pour les soignants que pour la majorité des enquêtés.

Les discours des personnes enquêtées, et des soignants montrent que pour la plupart des interlocuteurs, les liens entre le vecteur et la maladie sont connus. Cependant, les moustiques du village ne sont pas perçus comme des vecteurs potentiels. Ce seraient les moustiques des côtes qui transmettraient le paludisme.

> « Ici il n'y a pas de *tazomoka*, ce sont surtout les gens qui viennent d'émigrer vers les côtes qui en attrapent et qu'on traite ici. En général, le plus fréquent c'est le *tazo tsotra* ». [Soignant]
> « ici il n'y pas de maladie transmise par les moustiques ». [Femme, 49 ans]
> « Le *tazo* dû aux piqûres de moustiques existe dans la partie sud de l'île. A Sahamena, il n'y a pas de moustiques qui transmettent le tazo ». [Femme, 24 ans].

Selon les chiffres officiels, le nombre de cas de paludisme confirmé est limité au sein du CSBII de Sahamena.

Année	Nombre de consultations	Nombre de cas de fièvre	Test de diagnostic rapide positifs
**2009**	2022	74	4
**2010**	653	89	6
**2011**	737	95	3

Données SSD Fianarantsoa II, récolte de données en novembre 2012

2.1.2 Le paludisme est perçu comme une maladie contractée sur les côtes

Le paludisme est perçu par la plupart des enquêtés comme une maladie contractée dans les régions côtières, comme dans les parties sud[29] et sud-est[30] de l'île.

[29] Ikalamavony, Ihosy, Ambalavao…
[30] L'exemple évoqué était Manakara.

Les termes utilisés pour désigner le paludisme reflètent parfois cette référence aux zones côtières : l*e tazon'ny tambany*, qui veut dire la fièvre des gens de la côte ou des gens du bas, l*e tazo kilalaka* ou la fièvre de la danse kilalaka[31] ou la fièvre des gens du Sud et enfin le *tazon'ny sakafo* ou la fièvre contractée en consommant les fruits des côtes comme les mangues, les ananas, les litchis, et les jacques (fruits de *Artocarpus heterophyllus*).

> « Le *tazo* dû aux piqûres de moustiques existe dans la partie sud de l'île comme à Manakara. C'est le *tazo kilalaka*. Ici, il n'y en a pas ». [Homme, 24 ans]
> « Les gens ont l'habitude de dire *tazon'ny tambany*. Ils n'utilisent pas le mot *tazomoka,* parce que pour eux c'est une maladie qu'on contracte sur les côtes ». [Soignant]

Une petite minorité de personne a utilisé un nom qui est en lien avec les symptômes marquants : comme le *bemangovitra* ou tremblement intense[32], un terme utilisé pendant la grande épidémie de paludisme dans les années 1980 et qui est resté longtemps dans la conscience populaire ; qui apparemment existe encore.
Le terme *tazomoka* est connu mais n'est pas usité dans le fontontany.

2.1.3 Connaissances partielles sur les symptômes du paludisme

La plupart des personnes enquêtées considèrent qu'un état fébrile est le signe du *tazo* dû aux piqûres des moustiques.
D'autres symptômes ont été évoqués : la sensation de froid, les maux de têtes, le frissonnement, la température élevée et le sensation d'étouffement. Une minorité de personnes a évoqué l'apparition des boutons rouges ou les vertiges (qui ne correspondent pas forcément aux symptômes associés à cette maladie). Et une seule personne a évoqué la convulsion comme symptôme du paludisme.
La connaissance des symptômes du paludisme est donc encore partielle, du fait de la confusion entre le paludisme et la fièvre en général.

2.1.4 Diverses interprétations sur les causes du paludisme

2.1.4.1 Cause liée aux piqûres de moustiques

Nous avons mentionné plus haut que la majorité de nos interlocuteurs connaissent le lien entre la piqure des moustiques et la maladie, mais ces derniers reconnaissent que les moustiques du village ne sont pas perçus comme des vecteurs potentiels du paludisme.

2.1.4.2 Causes liées à la consommation des fruits des régions côtières

La consommation de fruits mûrs ou non mûrs venant des régions côtières est fréquemment évoquée par les enquêtés comme cause de paludisme. L'entourage, et surtout les personnes âgées, seraient, d'après les entretiens, à l'origine de ces croyances. Deux types d'explications sont donnés par ces villageois sur le lien entre la consommation de ces fruits et ce problème

[31] Danse des gens du Sud
[32] Termes que nous avons utilisé pour la traduction littérale de *bemangovitra*

de santé. D'une part, la consommation des fruits non mûrs provoquerait une intolérance de l'organisme. D'autre part, les fruits auraient été piqués par les moustiques, puis ingérés par l'humain, qui « avale » ainsi la maladie transmise par les moustiques.

2.1.4.3 Causes liées à l'hygiène et à l'environnement

Seuls quelques enquêtés ont avancé des causes liées à l'hygiène et à l'environnement.

> « Peut être que le *tazomoka* est transmis par l'air impur, ou par la malpropreté » [Villageoise, 28 ans].

Nous faisons l'hypothèse que cette perception résulte d'une confusion liée à une superposition des messages issus des campagnes de sensibilisation[33], durant lesquelles sont transmises différentes informations concernant plusieurs maladies.

2.2 Problèmes rencontrés par les soignants en matière de prise en charge du paludisme

2.2.1 Retard aux soins

Le retard aux soins a été évoqué par les soignants du CSBII comme l'une des problématiques rencontrées en matière de prise en charge non seulement en cas de fièvre ou de paludisme mais aussi pour d'autres maladies[34]. Ils affirment que les villageois ont l'habitude de recourir à l'automédication en premier recours, puis d'aller vers les tradipraticiens et finalement de s'adresser aux formations sanitaires en cas d'aggravation de la maladie (altération de l'état général et perte d'appétit). Ils associent souvent ce comportement à la confusion de la population entre le paludisme et la fièvre ou bien à des mauvaises habitudes, au manque d'éducation ou enfin aux problèmes financiers.
En revanche, les agents communautaires enquêtés affirmaient que les villageois s'adressaient aux formations sanitaires en premier recours, pas seulement en cas de paludisme mais également en cas de maladie en général qu'elle soit « simple » ou « grave »[35]. Ils expliquent ce comportement par la sensibilisation effectuée pour inciter les populations à systématiquement se rendre au centre de santé en présence d'un état pathologique quelconque.

2.2.2 Rupture de stock d'ACT

Une rupture de stock a été signalée, ce qui conduit parfois le médecin à utiliser un autre traitement comme le paludar® (sulfadoxine pyriméthamine) en cas de paludisme, un traitement également donné aux femmes enceintes atteintes de paludisme.

---

[33] Sensibilisation de masse faite par les soignants avant la consultation dans les formations sanitaires publiques, et campagne de sensibilisation faite par les agents communautaires.
[34] Comme par exemple : la toux, la diarrhée,…
[35] Ces termes sont difficiles à définir car ils renvoient à des réalités subjectives.

2.2.3 La santé : un secteur à améliorer

Pour certains soignants, le secteur de la santé a été délaissé, ce qui porte un impact direct dans l'accomplissement et la qualité de leur travail. Ces derniers ont par exemple fait référence aux manques de ressources humaines, à l'amélioration nécessaire des infrastructures sanitaires, à la motivation salariale des agents de santé et à la nécessité de considérer les motivations de ces divers acteurs de santé à différents niveaux. Par exemple, les agents de santé communautaires, agents aspergeurs et les chefs *fokontany* participent activement à la mobilisation de la population pour la promotion de la santé communautaire. Pour cela, ils doivent délaisser leurs activités agricoles, ce qui a un impact direct sur leur productivité (sur leur rendement et leur revenu mais également sur leur statut social), ce qui expliqueraient parfois leur absence ou leur indisponibilité.

2.3 Comportements thérapeutiques en cas de fièvre non favorables à la détection rapide du paludisme.

Les entretiens mettent en évidence divers comportements thérapeutiques en cas de fièvre : automédication, recours aux formations sanitaires publiques, recours aux tradipraticiens. Une seule personne affirmait ne jamais aller dans des formations sanitaires ni consulter de soignants.

2.3.1 Automédication en cas de fièvre pour cause de problème financier ou confiance accordée aux connaissances empiriques

L'une des raisons évoquées pour le recours à l'automédication est la contrainte financière. Les malades peuvent attendre 2 à 3 jours avant d'aller consulter un soignant, le temps de trouver de l'argent, étant donné qu'ils ne peuvent pas faire crédit dans les centres de santé, ou bien en espérant que la fièvre passe.

Médicaments consommés en automédication en cas de *tazo* ou de fièvre

En cas de fièvre, les villageois achètent des médicaments dans une épicerie comme le paracétamol, le cotrim® (sulfamethoxazole + trimethoprime), l'ibupamol®, (ibuprofène + paracetamol). Mais quelques uns affirment qu'ils sont réticents à en acheter à l'épicerie parce qu'ils craignent qu'il s'agisse de médicaments périmés.

> « Les gens ont l'habitude d'acheter du paracetamol, du cotrim, de l'ibupamol, en cas de *tazo.* Souvent ils ont l'habitude de dire : j'ai mal à la tête je ne me sens pas bien, j'ai de la fièvre, je vais acheter du cotrim à 50 *ariary* » [Soignant]

Plantes consommées en automédication en cas de tazo ou de fièvre

Comme traitement pour soigner le *tazo* : quelques uns disent également qu'ils boivent du café avec une cuillerée à café de *ranomena* (liquide fait à base d'os de

zébu), ou du café avec du piment ou bien du *ravina voampoa.* Ces pratiques reposent sur des connaissances empiriques.

Perception de l'efficacité des plantes médicinales

Selon quelques personnes enquêtées, l'utilisation des plantes médicinales n'est pas toujours efficace, voire elle peut aggraver certaines maladies, auquel cas ce n'est qu'après les avoir utilisées que l'individu se rend dans une formation sanitaire.

Le non-recours immédiat aux formations sanitaires semble lié à un attachement aux pratiques ancestrales, ainsi qu'aux difficultés financières. Un homme expliquait par exemple qu'il utilise des plantes pour « développer une stratégie » afin de faire face à ses problèmes financiers (en retardant le recours aux soins). Cette stratégie lui permet de disposer d'un peu plus de temps pour trouver de l'argent tout en espérant qu'après avoir consommé des plantes, la maladie sera réduite de moitié, ce qui réduira également le coût du traitement chez le médecin.

2.3.2 La médecine traditionnelle

Les difficultés financières ont été évoquées comme l'une des raisons pour lesquelles les villageois vont chez les tradipraticiens. Mais l'enquête montre que le coût du traitement chez un tradipraticien a pu s'élever à 20 000 Ariary, 100.000 Ariary et même jusqu'à coûter un bœuf. D'autres thérapeutes ne fixent pas une somme définie mais demandent aux patients de leur donner ce qu'ils veulent.

Nos interlocuteurs ont affirmé qu'il existe plusieurs tradipraticiens dans le village mais qu'ils ne les consultent pas en raison de leur croyance religieuse. Certains affirment qu'il s'agit de « charlatans » puisqu'ils ne guérissent pas complètement certaines maladies. Nous faisons l'hypothèse qu'il reste difficile pour ces enquêtés d'évoquer en entretien leur recours aux tradipraticiens.

L'une des raisons avancées par certains pour expliquer le recours à la médecine traditionnelle concerne la persistance de l'idée que la maladie vient d'un ensorcellement et que seuls les tradipraticiens peuvent la guérir. Selon une femme de 58 ans, l'idée de l'ensorcellement, lié à la réussite ou la jalousie, hante la plupart des villageois et serait cause de maladie.

Une similarité entre les symptômes des maladies dues à l'ensorcellement et ceux d'autres maladies peut être soulignée, comme l'illustre le discours de cette villageoise :

> « On reconnaît une maladie à un ensorcellement lorsqu'on frissonne, on a le vertige, la température, les courbatures et les manques d'appétit ». [Villageoise, 28 ans]

Cette similarité pourrait créer une confusion et influence les comportements thérapeutiques (consulter à la fois un médecin et un tradipraticien), les maladies dues à l'ensorcellement étant perçues comme des maladies qui ne peuvent être soignées que par les tradipraticiens.

> « Les médecins n'arrivent pas à guérir les maladies dues aux ensorcellements » [Homme, 58 ans]

> « Je préfère prendre à la fois les médicaments prescrits par les médecins et aller chez les soignants traditionnels parce que la médecine moderne peut soigner ce que le traditionnel ne peut pas soigner et inversement ». [Homme, 64 ans]

2.3.3 Les obstacles au recours aux formations sanitaires

Il apparaît que le plus souvent, les malades recourent aux formations sanitaires en cas d'aggravation de la maladie, ou lorsque l'automédication et/ou la médecine traditionnelle n'a pas été efficace.

2.3.3.1 Des réticences liées aux rapports soignants-soignés

L'enquête met en évidence chez certains enquêtés une certaine réticence à fréquenter les formations sanitaires, en lien avec la qualité de la relation soignant-soigné.

> « On a peur du soignant, il est très sévère, la plupart des gens n'osent pas trop aller à l'hôpital[36], lorsqu'il est là… ». [Femmes, 24 ans]

D'autres enquêtés, surtout des femmes, ont exprimé une réticence à fréquenter une formation sanitaire en raison du non-respect du tabou sur l'intimité par certains soignants et de l'utilisation de « phrases directes »[37] par rapport aux relations intimes des couples. Ces « phrases directes » sont mal perçues et gênent les femmes pendant les sensibilisations du planning familial, ou au moment de l'accouchement. En outre, au moment des accouchements, les gestes effectués par les soignants ou les termes utilisés peuvent mettre les femmes mal à l'aise.

2.3.3.2 L'influence des tradipraticiens

Les tradipraticiens dits « officiels »[38] font partie des personnes influentes et ont un statut social très important au sein de la communauté. Selon les informations que nous avons recueillies sur le terrain, le CSBII n'existe que depuis 13 ans environ. La médecine traditionnelle constituait jusque là le seul recours de soin possible en plus de l'automédication. Les soignants traditionnels ont une emprise sociale importante et ce jusqu'à maintenant, certains sont même intégrés dans l'équipe des mobilisateurs.
Actuellement la collaboration entre les soignants modernes et les guérisseurs traditionnels au sein de cette communauté est peu présente. Et les conflits d'intérêt se font ressentir. Les soignants traditionnels expriment leur crainte de perdre leur légitimité et leur reconnaissance. Comme le dit une matrone :

> « Nous n'allons pas nous disputer avec les soignants, ce que nous voulons c'est notre reconnaissance. On interdit les pratiques

[36] Elle utilise le mot hôpital pour désigner le CSB
[37] Phrases utilisées par les interlocuteurs
[38] Ce sont des soignants traditionnels officiels et qui sont reconnus au sein de la communauté. Certains ne font pas encore partie de l'association nationale des tradipraticiens.

traditionnelles comme le massage, l'accouchement par l'intermédiaire des matrones…C'est pourtant notre gagne pain. Bon nombre de gens ont été guéri grâce à notre intervention, les gens ne nous oublient pas, ils se souviennent toujours du bien qu'on leur a fait. C'est très difficile en même temps de repousser les gens qui souhaitent venir vers nous pour se faire consulter, alors que c'est interdit. Les soignants ont déjà prévu de faire une réunion avec nous, mais jusqu'à maintenant on attend… » [Matrone, 56 ans]

2.3.3.3 Devoirs sociaux et festivités liées aux décès

Les communautés accordent une importance capitale à l'accomplissement des devoirs sociaux pendant les festivités liées aux décès, pendant lesquelles règne le principe de don et contre-don nommé *aterikalao*[39]. Selon les explications de certains soignants, un décès dans la famille entraîne par exemple l'obligation de tuer un zébu pour le partager avec la communauté. Toujours selon lui, face à un problème financier, les villageois préfèrent garder leurs zébus pour en avoir à disposition en cas de décès, que les vendre afin d'obtenir de l'argent pour des soins en cas de maladie grave.

2.3.3.4 Le rôle de la prière

La religion, par le biais de la prière, peut constituer un recours thérapeutiques. Ainsi, une femme nous a confié qu'elle et sa famille ne fréquentent aucun centre de santé et ne consultent aucun soignant par conviction religieuse[40].

« Cela fait 18 ans que je ne suis pas allée voir un soignant pour me faire soigner…Mon mari a été atteint d'une jaunisse, il était guéri après trois mois sans consulter ni se faire traiter par un soignant. Nous avons seulement eu recours à la prière, je lui donnais en plus du *ravina voampoa* ou feuille d'angive, puis je l'ai encouragé à manger des légumes et des fruits pour faire revenir sa force ». [Femme, 24 ans]

2.3.4 Connaissances du traitement

Seule une minorité des personnes enquêtées connaît le nom ACT et les médicaments administrés en cas de *tazomoka*. La source d'information qu'elles ont évoquée était surtout la radio.

[39] Je donne et je reçois
[40] Religion : Jesosy famonjena ou Jésus sauveur

2.4 Les pratiques préventives de la population

2.4.1 La CAID

2.4.1.1 Utilité perçue de la CAID : tuer les puces et les cafards

La CAID est davantage perçue par la majorité des personnes enquêtées comme un insecticide qui tue les puces et les cafards, mais pas spécifiquement les moustiques. Leurs préoccupations se portent davantage sur le fait de se débarrasser des puces et des cafards, que sur les moustiques.

> « …l'objectif du DDT[41], je ne sais pas. Pour nous, ce sont les puces et les cafards qui nous gênent, il nous arrive même d'acheter des insecticides à 5000 ariary dans la pharmacie à Fianarantsoa pour pouvoir s'en débarrasser ». [Femme, 58 ans]

2.4.1.2 Désagréments causés par la CAID

Quelques désagréments causés par la CAID ont été signalés par certains enquêtés, comme : la persistance des odeurs, décrites comme insupportables et qui seraient à l'origine de maux de tête et de toux. D'autres enquêtés ont rapporté que le produit utilisé tuait les œufs de poule au moment de la ponte.
Malgré ces désagréments, la CAID est dans la majorité des cas acceptée puisque la décision émane des autorités locales qui exercent un pouvoir social important au sein de la communauté. Le Président de *fokontany* a néanmoins signalé quelques cas de refus.

> « On accepte parce que c'est le *Fanjakana*[42], de toute façon si on n'accepte pas, on est perdant, puisque c'est pour notre bien qu'ils font ça » [Homme, 24 ans].

2.4.1.3 Perception sur l'efficacité de la CAID

La majorité des personnes enquêtées ont exprimé leur sentiment que le produit n'était pas efficace. Rares sont ceux à avoir affirmé le contraire. La plupart des enquêtés établissent un lien entre la clarté et l'efficacité du produit : plus le produit est clair, moins il serait efficace. Cette perception fait naître une suspicion au sein de la population sur l'existence de trafic d'insecticide qui serait le fait des agents aspergeurs, comme l'exprime ce villageois :

> « Je trouve que le produit n'est pas efficace, surtout lorsqu'on constate que le produit est clair. Au lieu d'utiliser un sachet pour une maison, ils utilisent peut être 1 sachet pour 2 à 3 maisons ». [Homme, 24 ans]

---
[41] La CAID est connu sous le nom de DDT
[42] Le *Fanjakana* = l'Etat

Un autre villageois affirmait avoir acheté une petite quantité de poudre de DDT[43] parce que ce dernier est perçu comme étant plus fort et efficace pour tuer les puces que le produit utilisé lors de la CAID.

> « Nous avons constaté par hasard que la poudre de DDT tue les puces, si bien que nous en avons acheté. Les agents aspergeurs nous disaient qu'ils n'ont pas le droit d'en vendre. Nous ne voulions acheter qu'une tout petite quantité si bien qu'ils ont accepté et nous l'a vendu à un prix très modeste ». [Homme, 64 ans]

2.4.2 Le PECIMEc

2.4.2.1 ACT périmés

Concernant la PECIMEc, les Agents communautaires dont certains assurent également la gestion de la pharmacie à gestion communautaire ou PHAGECOM affirment qu'ils disposent de TDR et d'ACT. Un des soignants a souligné que les ACT qui sont destinés aux enfants de moins de 5 ans seront périmés peu de temps après l'enquête. Certaines mères que nous avons rencontrées ne connaissaient pas l'existence de ces Agents communautaires. D'autres villageois les perçoivent comme des agents qui distribuent des vitamines, et qui font des vaccins pour les enfants de moins de 5 ans. Notons que la formation de ces agents avait eu lieu en 2011.

2.4.2.2 La sensibilisation pour la lutte contre le paludisme n'est pas une priorité selon un agent communautaire

Les avis des agents communautaires enquêtés sur l'utilité de sensibiliser la population sur le paludisme sont partagés. Un agent communautaire a affirmé qu'il n'est pas utile de sensibiliser les villageois sur le paludisme parce que c'est une maladie qui n'existe « pas vraiment ».

> « Le *tazomoka* n'existe pas vraiment, ma sensibilisation est surtout axée sur l'allaitement, l'alimentation, le respect de l'hygiène et l'utilité de la planification familiale ». [Agent communautaire]

D'autres agents communautaires demandent au contraire davantage de formations sur ce problème de santé en matière de prise en charge et d'utilisation de TDR.
D'après les entretiens menés avec les villageois, les Agents communautaires ne constituent pas des sources d'informations sur le paludisme. Ce sont surtout les soignants du CSB, les campagnes de distribution de MID, la radio (qui a été cité auparavant comme source d'informations sur le nom du médicament) et enfin l'entourage qui sont cités parmi les canaux d'informations sur le paludisme.

2.4.3 Quelques moyens évoqués pour se protéger contre les piqûres de moustiques

La dernière campagne de distribution de moustiquaires a été réalisée en 2007 à Sahamena. Seules les femmes enceintes et celles qui avaient des enfants de moins de 5 ans ont pu en

[43] La CAID est connu sous le nom de DDT

bénéficier. Cependant nous avons effectué quelques observations et posé des questions auprès de nos interlocuteurs par rapport à l'utilisation de la MID.
La plupart des enquêtés affirment utiliser la moustiquaire pour se protéger des piqûres des moustiques (mais pas spécialement contre le *tazomoka*) pendant la saison des pluies durant laquelle il y a une prolifération de moustiques. D'autres moyens locaux sont aussi utilisés par certains afin de se débarrasser des moustiques comme :

- suspendre les feuilles de tomates sur les fenêtres pour faire fuir les moustiques avant de les fermer.
- fermer les fenêtres avant la tombée de la nuit.
- brûler des plantes pour faire de la fumée qui fait fuir les moustiques.
- utiliser les insecticides en spirales (moins utilisés en raison du coût).

2.4.4 « Anticorps » contre le paludisme : les petites bichiques et l'ananas

Quelques personnes ont mentionné l'existence d'anticorps contre le paludisme. Ces anticorps seraient induits par la consommation de petites bichiques et d'ananas.
L'un de nos interlocuteurs a affirmé avoir entendu par une personne de son entourage que les bichiques[44] peuvent être des anticorps contre le paludisme et la bilharziose. D'après cet interlocuteur, le fait d'en manger souvent et cuits[45] habituerait l'organisme à affronter les parasites et renforcerait l'immunité.
Un autre enquêté mentionne l'ananas comme source potentielle d'anticorps. Il raconte avoir eu le *bemangovitra* en 1985. Il savait que cette maladie était transmise par le moustique. Il aurait observé que les moustiques étaient très attirés par l'ananas et qu'ils lui transmettaient des parasites par l'intermédiaire de leurs piqûres. Il aurait fait l'expérience de consommer fréquemment de l'ananas afin de s'immuniser contre le paludisme, ce qui aurait été efficace : il affirme en effet ne plus avoir été atteint de paludisme en dépit des piqûres de moustiques.

[44] Alevins consommés à Madagascar.
[45] Ne nécessitent pas une préparation spéciale, mais cuits tout simplement

3.1 Le paludisme n'est pas perçu comme une maladie qui touche la population

Pour le cas du *fokontany* de Sahamena, le paludisme n'est pas perçu comme une maladie qui touche la population du *fokontany*. Il est en revanche perçu comme une maladie qu'on contracte sur les côtes du pays, par l'intermédiaire de piqûres de moustiques ou en consommant des fruits mûrs ou non mûrs. Il apparaît que les connaissances de la population enquêtée sur le paludisme sont partielles, notamment en ce qui concerne la reconnaissance des symptômes et l'étiologie de la maladie. Les connaissances ne sont pourtant pas nulles puisque la majorité des enquêtés établissent un lien entre le vecteur et la maladie. Par ailleurs, les pratiques thérapeutiques de la population enquêtée en cas de fièvre ne contribuent pas à la détection rapide du paludisme, car la plupart attendent généralement que la maladie s'aggrave avant de se rendre dans une formation sanitaire, en raison de différents facteurs, économiques, sociaux et culturels.
En prenant en compte les constats effectués dans cette zone d'étude, il semble que deux types d'actions contribueraient à améliorer la prévention et la prise en charge du paludisme.

3.2 Prioriser la sensibilisation sur le paludisme importé

Dans cette zone, Il apparaît important de prioriser les actions de sensibilisation concernant le paludisme importé notamment sur les causes, les risques, et les comportements à adopter, afin que la population puisse se protéger en cas de déplacements dans les zones à risque de transmission. Les connaissances par rapport aux zones endémiques ou zones à risque de transmission pour nos interlocuteurs étant limitées, il serait important de diffuser des informations sur ces zones. Des affiches mises à jour régulièrement pourraient notamment être placées dans les endroits les plus fréquentés (CSB, Fokontany, Mairie) en fonction de la saisonnalité du paludisme. La population pourrait ainsi informer ses proches ou se protéger en cas de déplacement dans ces zones.

Cette sensibilisation pourrait s'accompagner d'une information sur les résistances causées par l'automédication ou sur les modes de transmission du paludisme afin notamment de limiter l'importance des interprétations personnelles.

3.3 Importance des relations avec les tradipraticiens « officiels »

Le conflit d'intérêt ressenti par les soignants traditionnels pourrait affecter la fréquentation de la population dans le CSB. Il serait nécessaire de mener une réflexion sur la manière d'intégrer les guérisseurs traditionnels dans la prise en charge et la prévention du paludisme.
Cet effort va de pair avec l'amélioration de la qualité des relations soignants-soignés et des pratiques professionnelles. Cette amélioration est nécessaire afin de renforcer l'accès aux soins et la compréhension, par la population, des enjeux de prévention et de recours aux soins.

IV. ZONE 4 : MANANJARY

Le rapport sur la zone d'étude de Mananjary a été rédigé par Chiarella Mattern.

Table des matières

1. Contexte et generalites **75**
1.1 Situation géographique et caractéristiques socio-économiques 75
1.2 Les stratégies de lutte contre le paludisme 76
2. Note Methodologique **77**
2.1 Changement dans le choix du *fokontany* 77
2.2 La semaine de distribution de moustiquaires 77
3. Maladies et itineraires therapeutiques **78**
3.1 Maladies simples/maladies graves 78
3.1.1 L'évolution de la fièvre en trois temps 78
3.1.2 Diarrhée 79
3.1.3 Parasites 79
3.2 Itinéraires thérapeutiques 79
3.2.1 « La débrouille » est plus économique : l'automédication 79
3.2.2 « Si la maladie reste, le doute s'installe » 80
3.2.3 Le recours aux structures sanitaires en cas d'aggravation de l'état 82
3.3 Perception de l'efficacité des traitements 83
3.4 La santé des enfants : une affaire de femmes 83
4. Le paludisme : un probleme qui semble resolu **84**
4.1 Une maladie connue mais un traitement méconnu 84
4.2 Une faible prévalence observée 86
4.3 Identification de la MID comme raison de la diminution du paludisme 87
4.4 Absence de TPI dans les discours des femmes 87
5. La moustiquaire fait partie du decor **88**
5.1. Mananjary : un terrain favorable à la sensibilisation 88
5.1.1 Utilisation assidue de la moustiquaire 88
5.1.2 L'adoption de la moustiquaire : un *fombandrazana* 88
5.1.3 Préférences en matière de moustiquaires 89
5.1.4 Autres moyens de protection contre les piqûres de moustique 89
5.2 Déroulement de la distribution 90
5.2.1 Evaluation rapide des messages retenus 90
5.2.2 Usages faits des anciennes moustiquaires 91
6. Conclusions **92**
6.1 La moustiquaire comme partie intégrante du décor 92
6.2 Paludisme : un problème réellement résolu ? 92
6.3 Blocages à l'accès au traitement 92
7. recommandations **93**
7.1 Renforcement du lien entre convulsions, fortes températures et paludisme 93
7.2 Renforcement de la gestion des soins à l'échelle communautaire 93
7.3. Mise en évidence des mécanismes de gratuité des soins existants 94
7.4 Prise en compte des spécificités culturelles pour les opérations de distribution 95
7.5 Rendre « attractifs » les messages de sensibilisation 95

1.1 Situation géographique et caractéristiques socio-économiques

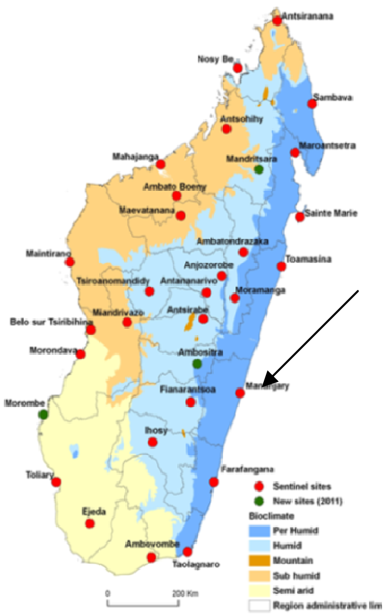

Le *fokontany* dans lequel nous avons travaillé regroupe 4 familles et est composé de 1183 personnes. Les habitations traditionnelles sont sommaires, construites en *ravinala* « arbre voyageur », le toit se construit avec les feuilles de l'arbre (*raty*), le mur avec les pétioles de *ravinala* ou en bambou et le plancher se fait avec le tronc de l'arbre.

La majorité des villageois dorment sur des nattes. Une partie des familles a aménagé une cuisine extérieure mais pour la grande majorité, c'est à l'intérieur de l'habitation principale que les repas sont préparés.

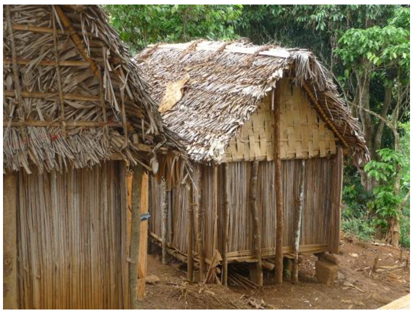

Quand les enfants atteignent l'âge de 14 ans, ils quittent la maison familiale pour s'installer dans une habitation à proximité de celle-ci, souvent construite par le père de famille. Tant qu'il n'est pas marié, le jeune continue à partager le repas familial. Les parents subviennent à l'achat des matériaux pour la construction de la maison mais c'est au jeune qu'incombe la responsabilité de l'équipement. Une fois mariée, la jeune fille ira rejoindra le lieu de vie de la famille de son mari. Le jeune homme une fois marié se construira une maison plus grande, non loin de celle de ses parents.

La principale source de revenu est l'agriculture et plus précisément la culture de riz. D'août à novembre l'homme et la femme travaillent ensemble aux champs. La récolte de riz de contre-saison se fait en décembre. Cette récolte coïncide avec le début de la saison de pluies, et il y a un risque d'inondation et de mauvaises récoltes ; l'autosuffisance peut durer juste 2 mois, ce qui fait que la période de soudure a lieu en février jusqu'à la prochaine récolte du riz. Les fruits à pain et le maïs frais sont alors consommés pour substituer le riz. La grande récolte de riz s'étend de novembre à mai. A partir du mois de juin, le riz n'est pas cultivé les patates et brèdes sont alors consommées en priorité.

Les activités principales du quotidien de la femme sont la préparation des repas et l'agriculture. Elles développent également des activités génératrices de revenus telles que la vannerie ou le maraichage. Les hommes travaillent quant à eux dans les champs toute l'année, défrichent pour préparer les cultures, mettent le feu à leur champs et ensuite, sèment et récoltent.

Une journée type commence avec le lever du soleil et est rythmée par les travaux dans les champs et la préparation des repas. Vers 16h ou 17h les paysans reviennent au village, prennent leur repas du soir vers 19h et se couchent vers 20h ou 21h. Les soirs d'été, les adultes discutent dehors pendant que les enfants jouent.

1.2 Les stratégies de lutte contre le paludisme

Le paludisme pour la région de Mananjary et plus globalement pour l'ensemble de la côte Est relève du faciès équatorial. La zone est caractérisée par une endémicité forte et une transmission continue. Les stratégies de lutte contre le paludisme mises en place dans la région sont la MID (distribution de masse en 2009 et 2012 et distribution de routine durant les CPN et campagnes de vaccination) et le TPI. La PCIMEc est également opérationnelle depuis plus d'un an dans cette zone. Les ACT sont gratuitement offerts aux patients testés positifs au sein des formations sanitaires publiques ; auprès des Agents communautaires c'est l'Actipal® (artesunate-amodiaquine) qui est vendu à 100 Ar. Ce coût est considéré par les villageois comme pas cher, mais peut constituer un obstacle au traitement.

2.1 Changement dans le choix du *fokontany*

CARTE DE LA COMMUNE DE TSARAVARY

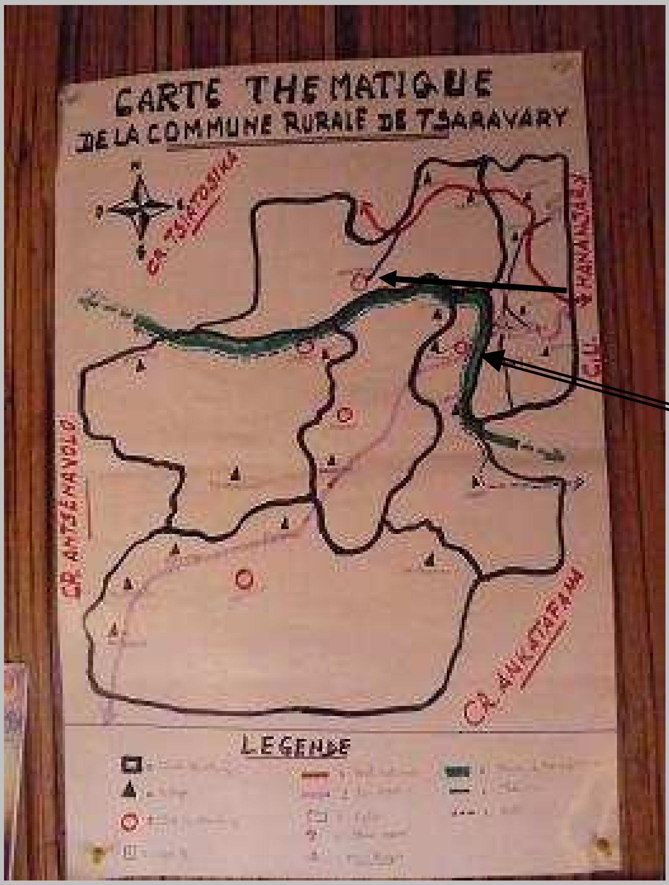

Le *fokontany* d'Andranomaresaka, de la commune de Tsiatosika choisi en premier lieu a été rendu inaccessible à cause de la dégradation du premier pont menant vers le village. Un second *fokontany* d'une autre commune (Tsaravary) avait été identifié durant la mission préparatoire correspondant aux critères requis : Ampasimbola.

Ce *fokontany* est constitué de plusieurs hameaux, dont Ambohitsara, situé de l'autre côté de la route, accessible via une piste de 2 km. Le hameau principal ainsi que ce dernier ont été investigués par souci de complétude d'un panel diversifié de comportement de recours au soin. La commune est coupée d'est en ouest par le fleuve Mananjary (en vert sur la carte) et rend, en saison des pluies, le CSB II accessible en pirogue uniquement pour les 4 *fokontany* du sud.

2.2 La semaine de distribution de moustiquaires

Notre séjour à Mananjary a concordé avec la semaine de distribution de moustiquaires prévue et organisée par le Programme nationale de lutte contre le paludisme (PNLP) en partenariat avec PSI et le BDEM du lundi 26/11 au samedi 01/12. Dans l'ensemble du district les distributions ont été mises en place par les Agents de santé communautaires suite à un recensement réalisé en août dernier, visant à comptabiliser le nombre de membres par ménages. Des banderoles, affiches et spots radio indiquaient les lieux et dates de distributions. Nous avons profité de notre présence durant cet évènement ponctuant la lutte contre le paludisme dans la région pour sonder rapidement les préférences et réactions quant à la qualité des moustiquaires reçues et à l'organisation globale de la distribution.

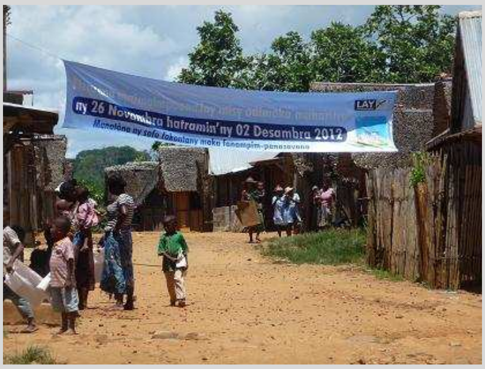

3.1 Maladies simples/maladies graves

Les maladies considérées comme fréquentes sont dans l'ordre de fréquence d'apparition : la fièvre simple, la fièvre forte entrainant des convulsions, les maladies diarrhéiques et les parasites[46].

Une maladie est considérée comme grave lorsque les premiers symptômes soignés sur base de comprimés (paracétamol et quinine principalement) ne passent pas et que la maladie se généralise. Les signes de gravités cités sont le manque d'appétit, le refus de boire, l'apparition de tremblements et les difficultés pour respirer.

> « *Une maladie est grave quand on prend des médicaments aujourd'hui et que l'état ne s'améliore pas. Dans ce cas, il faut aller à l'hôpital car les médicaments qu'on donne au village ne sont peut-être pas adéquats. Certaines personnes meurent en cours de route car elles ont trop tardé* » [Femme, 26 ans].

L'accessibilité géographique et l'accessibilité financière sont les facteurs cités conduisant à l'aggravation d'une maladie au départ simple. En d'autres mots, selon nos interlocuteurs, il y a peu de maladies que les médicaments ne peuvent pas soigner. Les réels obstacles sont alors l'argent et la distance des formations sanitaire. L'idée d'aggravation de maladie due au retard de prise en charge est connue par les interlocuteurs qui ont conscience du danger que ça présente.

> « *La diarrhée ça fatigue. Quand on est loin du docteur ça tue, mais quand on est proches ça peut se soigner* » [Jeune homme, 25 ans].

Une différence est faite dans les discours des interlocuteurs, entre maladies simples et graves. Les premières (toux, parasites, paludisme, etc.) sont soignables par l'automédication à base de comprimés achetés au sein des réseaux de distribution de proximité (vendeurs ambulants, épiceries, Agents communautaires) et/ou à l'aide de tisanes/inhalations. Celles-ci sont considérées comme des maladies « pas chères » à prendre en charge. Les secondes (diarrhées, fièvres fortes, etc.) sont nécessairement référées au médecin qui les traitera à l'aide d'injections. Ces dernières engendreront des frais plus élevés.

3.1.1 L'évolution de la fièvre en trois temps

Selon les dires de nos interlocuteurs, la fièvre simple, ou *marokoditra* (avoir la chair de poule), peut évoluer en fièvre forte *tazo mahery* et finir en convulsions, le *tratravorina*[47]*,* qui se manifeste par une perte de conscience et des vertiges. Le lien entre ces différentes étapes est connu : si la fièvre dans sa forme simple n'est pas soignée dès les premiers signes, elle risque de s'aggraver dans des formes sévères et peut entrainer la mort. Les convulsions sont

[46] Les problèmes de tension, maux de tête, de dents, de dos, la tuberculose, la bilharziose, le paludisme, la toux et douleurs aux articulations ont été cités.

[47] Traduisible en français par le battement d'aile de l'oiseau en référence aux mouvements des bras observés pendant les convulsions.

interprétées comme étant les signes d'une maladie à un stade avancé, ils sont un « signe de malheur ».

> « *Les convulsions se reconnaissent car les yeux montent, les mains tremblent, les dents sont fermées. Cet état demande beaucoup de soins. C'est souvent dans cet état là que l'enfant est amené chez docteur. Ça touche seulement les enfants, pas les adultes, ils peuvent en mourir* » [Femme, 52 ans].

La première forme de fièvre se manifeste par une sensation de froid, malgré une température élevée du corps. Elle apparait suite à une grande fatigue, à une exposition trop longue au soleil ou à des changements de températures (spécifiques aux périodes d'entre-saisons). Cette fièvre est « facilement soignable » à l'aide de comprimés, tandis que la seconde doit être spécifiquement prise en charge par le médecin qui octroiera un traitement à base d'injections. Le recours au médecin doit être fait le plus rapidement possible dès l'apparition des convulsions qui peuvent entrainer la mort. Les parasites et maladies respiratoires ont été cités comme causes de cette fièvre forte. Les enfants et personnes âgées sont plus touchés par ces fièvres.

3.1.2 Diarrhée

La diarrhée est considérée comme une maladie chère à prendre en charge. Par conséquent, les malades ont un recours tardifs aux soins adaptés. Les Agents communautaires peuvent prendre cette maladie en charge quand il s'agit d'un patient de moins de 5 ans. Quand la maladie persiste, ceux-ci réfèrent à un centre de santé. Selon les dires de nos interlocuteurs, la diarrhée est causée par les inondations et l'eau sale consommée. Tout le monde est susceptible d'être atteint.

3.1.3 Parasites

Les vers « longs et plats » ont souvent été cités. On est infesté pas ces vers suite à la consommation d'aliments insuffisamment cuits.

3.2 Itinéraires thérapeutiques

3.2.1 « La débrouille » est plus économique : l'automédication

> « Je préfère me faire dès aujourd'hui à l'idée que si mon enfant tombe malade il pourrait mourir comme ça je suis préparée. Car l'argent bloque tout et je n'en n'aurai pas suffisamment pour le soigner » [Jeune Femme, 26 ans]

Le premier réflexe suite à l'apparition des signes d'une maladie est l'observation au fil des heures de l'évolution de celle-ci et l'automédication en fonction des symptômes observés. L'entourage (membres de la famille) aura un rôle important dans la prise de décision concernant l'orientation à donner à l'itinéraire thérapeutique, des comptes rendus réguliers seront donnés par la mère aux voisins au fil de l'évolution de la maladie. C'est l'expérience de symptômes similaires ressenti qui définira le nom des médicaments à acheter, toujours sous les conseils de l'entourage.

Dans le cas de notre *fokontany*, il existe plusieurs lieux d'achat de médicaments : le recours préféré est l'instituteur qui dispose d'une pharmacie relativement complète, le second est un épicier situé dans un hameau éloigné et enfin, les marchands ambulants profitent du jour de marché à la commune pour passer dans les villages et vendre leurs articles. Le recours à l'un de ces lieux d'achat dépend de la confiance faite aux médicaments dispensés. Une différence est faite entre les bons et les mauvais médicaments. Ces derniers sont reconnaissables par leur inefficacité expliquée par le fait que le médicament est ouvert depuis trop longtemps, le principe actif s'est donc envolé. Les médicaments de l'instituteur ont bonne réputation, ils sont considérés comme plus efficaces. Celui-ci insiste sur le fait qu'il ne prend en charge que les maladies simples, en cas de fièvre forte par exemple (persistance malgré paracétamol), il réfère tout de suite à une formation sanitaire. L'Agent communautaire dispose de médicaments également pour la prise en charge des enfants de moins de 5 ans.

L'argent est le premier argument avancé au non recours aux formations sanitaires. Dès qu'un villageois tombe malade, il devient une charge pour sa famille qui devra endurer tous les frais liés aux recours aux soins. Les frais de déplacement jusqu'au CSB de la commune s'élèvent à 1500 Ar, les frais de consultation varient entre 1000 et 3000 Ar et enfin, les frais liés à l'achat des médicaments préconisés par le médecin sont également considérés comme des dépenses trop élevées. Certains patients devront également débourser de l'argent pour les nuitées passées au centre de santé (1500 Ar/nuit), ainsi que les coûts liées aux allers-retours des membres de la famille, en charge des repas du patient.

> « *On essayera toujours d'abord de nous débrouiller car à l'hôpital il faut amener 10 000 Ar au moins pour se faire soigner. Je sais que le docteur ne fera rien sans argent donc je n'y vais pas* » [Femme, 26 ans].

L'accessibilité géographique est un obstacle supplémentaire dans les itinéraires de recherche de soins. La structure la plus proche, le CSB des Sœurs à Marovahy, est préférée de par sa situation géographique permettant aux malades de faire l'aller-retour à pied en une journée (une heure de marche pour y accéder). Cependant, les consultations et médicaments y sont plus chers qu'au CSB 2 de la commune (1h30 de marche). L'hôpital, lui, n'est accessible qu'en transport (15 km).

Globalement, le recours aux formations sanitaires est considéré comme trop cher. Quand quelqu'un tombe malade on préfèrera donc le prendre en charge à moindres coûts. Cette période d'automédication permettra également de « chercher de l'argent ».

> « *On en peut rien faire d'autre que d'assister à la maladie qui s'aggrave car on n'a pas d'argent* » [Homme, 65 ans].

3.2.2 « Si la maladie reste, le doute s'installe »

Le recours au guérisseur traditionnel nous a semblé controversé à Ampasimbola. Comme dans le reste du pays, les nombreuses églises chrétiennes ou dérivées portent avec elles un discours stigmatisant le recours aux pratiques ancestrales. Les leaders religieux demandent alors à leurs fidèles de poser un choix : l'église ou les ancêtres.

> « *Ici on est chrétiens. On ne peut pas aller voir le guérisseur et en même temps aller à l'église. Mais le savoir-faire transmis par les ancêtres peut être pratiqué* » [Homme, 45 ans].

Dans le cas de ce *fokontany*, la réponse la plus fréquemment reçue, à la question d'un éventuel recours vers un guérisseur était « *nous sommes chrétiens, nous n'allons plus chez le guérisseur* ». Au-delà de l'appartenance à une église chrétienne, certains remettent en question la réelle efficacité d'une prise en charge par le guérisseur. Leurs discours (peut-être influencé par les leaders religieux) font des guérisseurs des charlatans incapables de soigner mais prêts à tout pour soudoyer l'argent des malades. Pour les autres par contre, le recours au guérisseur se maintient en raison du prix faible des consultations.

> *« Je sais que le docteur ne fera rien sans argent donc je n'y vais pas. Le guérisseur lui, ne demande que 200 ou 300 Ar* » [Femme, 26 ans].

Mais si la maladie persiste malgré les efforts déployés pour la guérir (automédication avec comprimés ou suite à une consultation auprès d'un médecin), le doute s'installe.

> « *En fait les gens ne se font pas confiance les uns les autres ici. Si quelqu'un est malade, ils vont croire que c'est la sorcellerie. Ils vont d'abord essayer de régler le problème eux-mêmes via l'automédication et si ça ne marche pas, ils iront voir le guérisseur qui leur dira que c'est à cause des ancêtres qui ne sont pas contents. Si cette étape-là ne marche pas non plus alors seulement ils iront voir le docteur mais ça rend difficile la prise en charge du patient car le guérisseur traditionnel a fait des soins à sa façon »* [Soignant, 50 ans].

La méfiance et la jalousie semblent détenir une place importante au sein des rapports sociaux : on ne peut jamais affirmer qu'on est à l'abri d'un sort, même de la part d'un ami ou un membre de la famille.

ENSORCELEE PAR SA MEILLEURE AMIE

« J'avais mal aux dents et j'ai été voir un guérisseur traditionnel qui m'a dit que c'était parce que quelqu'un avait enterré un objet dans ma maison pour m'ensorceler. Le guérisseur est venu et il a effectivement trouvé un miroir enterré sous ma cuisine. Quand on fait appel à un guérisseur, il ne dit jamais qui l'auteur de l'acte car il ne veut pas engendrer des conflits au sein du village. Mais je crois que c'était ma meilleure amie de l'époque, qui est décédée peu après. Elle a toujours été jalouse car mon mari est parfait, il est fidèle et gentil. Ce n'est pas l'argent qui lui faisait envie mais plutôt mon mari, elle voulait prendre ma place. Je n'ai jamais su de quelle maladie elle est morte » [Jeune femme, 30 ans].

Le guérisseur soigne spécifiquement les maladies qui apparaissent soudainement et qui pourraient être interprétées comme étant un ensorcellement : fièvre forte, convulsions, tremblements, etc. Dans ces cas précis de suspicion d'ensorcellement, la famille du malade fait appel au guérisseur afin de mettre un terme au sort qui est jeté.

Il y a donc une pluralité de discours et d'attitudes vis-à-vis des guérisseurs. Une attitude cependant reste observable et est apparue comme récurrente : la nécessité d'être prudent et de tenir compte de l'emprise que peuvent avoir certains voisins jaloux sur sa propre santé, et l'ensorcellement comme cause possible de la maladie ou de la mort. Ainsi, dès

qu'une maladie dépassera le stade de l'explicable et du tangible (ex : persistance d'une maladie malgré traitement), cela pourrait être un sort jeté par un voisin.

> « *Il arrive que quand le docteur ne parvient pas à détecter le problème du patient il pointe la cause de l'ensorcellement et lui conseille alors, en sous-entendu de se référer plutôt à un guérisseur* » [Instituteur, 38 ans]

Le recours aux guérisseurs intervient soit simultanément au recours à une formation sanitaire, soit juste après l'automédication, avant le CSB, afin d'éviter des frais liés aux consultations. Si le recours au guérisseur est controversé, les savoirs faires transmis par les ancêtres en matière de santé, les *ody gasy* se pratiquent encore : tisanes et inhalations.

> « *C'est sûr que les gens prennent médicaments et simultanément vont voir les guérisseurs mais ne le diront pas à haute voix car ils sont chrétiens »*, [Instituteur, 38 ans].

3.2.3 Le recours aux structures sanitaires en cas d'aggravation de l'état

> « *Si après avoir pris les médicaments l'enfant ne va pas jouer et ne se sent pas mieux, on va chez le docteur* » [Femme, 52 ans].

Comme nous l'avons dit, dans le cas du *fokontany* d'Ampasimbola, le malade désireux de recourir à une structure sanitaire a trois possibilités : CSB II privé de Marovahy (1h de marche), CSB II public (commune de Tsaravary 1h30 de marche) ou l'hôpital de Mananjary (15 km).

Pour les raisons évoquées ci-dessus, la consultation d'un médecin au sein d'une formation sanitaire se fait donc en ultime recours, entre 24 h et plusieurs jours après l'apparition des premiers symptômes. Le danger que présente cette prise en charge tardive semble être connu des interlocuteurs et est pointé comme étant un problème de santé publique important par les soignants également. Un interlocuteur précise « *ce n'est pas une question de méfiance envers le docteur car souvent on revient de chez lui guéri*».

Nous avons observé de la part des patients une connaissance extrêmement faible du contexte de soin (nom des maladies contractées et des traitements pris lors du passage chez le médecin) qui pourrait venir du manque d'informations transmises par le médecin et du peu d'intérêt qu'ont les patients de mettre un nom sur leurs maux. Cela est valable pour la propre santé du malade et de son entourage.

> *« Nous n'avons pas l'habitude de demander au docteur après consultation d'où vient la maladie. Je n'ai pas fait beaucoup d'études donc je n'ai pas l'habitude de faire des analyses sur l'origine de la maladie. Je crois ce que le docteur me dit, c'est lui qui a fait des études sur la santé »* [Homme, 65 ans].

> « *Quand on va rendre visite chez le docteur, il ne donne pas d'explication sur la cause, le nom, etc. de la maladie. Il donne juste le traitement. Le docteur demande au malade les symptômes, de quoi il souffre sans même le toucher ou l'ausculter* » [Femme, 26 ans].

Ce manque d'échange entre soignant/soigné a également été mis en évidence par cette jeune femme, qui regrette la distance que le médecin instaure entre lui et le malade.

Un soignant identifie une difficulté dans le suivi des maladies « simples », qui n'exigent pas une prise du traitement sous supervision médicale : dès que le patient se considère comme guéri il arrêtera le traitement, même s'il n'est pas terminé. Nous avions observé le même phénomène concernant la prise des ACT à Antsohihy.

3.3 Perception de l'efficacité des traitements

La préférence va ici, à l'opposé de ce que nous avons pu observer à Antsohihy, vers les traitements par injections. Ceux-ci sont considérés comme les seuls efficaces. Si un traitement par comprimé leur est proposé, les patients craindront de devoir revenir régulièrement au centre de santé pour changer de traitement. L'existence de traitements gratuits (prise en charge de la malnutrition aiguë, de la tuberculose, du paludisme, etc.) n'est pas connue et mériterait d'être soulignée, afin de supprimer l'obstacle financier, au moins concernant ces maladies.

3.4 La santé des enfants : une affaire de femmes

UNE FEMME ATTEND SA MOUSTIQUAIRE

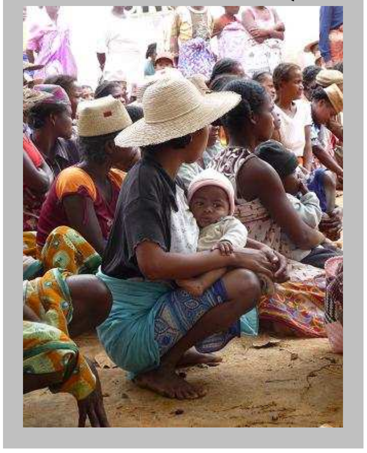

A l'instar de ce que nous avons observé à Antsohihy, ce message fait l'unanimité : ce sont les femmes qui sont en charge de la santé des enfants. Elles font des comptes rendus réguliers à leurs maris mais la décision concernant le recours au soin est de leur responsabilité. C'est elles qui passent le plus clair de leur temps avec les enfants et sont donc les mieux à même de prendre des décisions[48] concernant la santé de leurs enfants.

[48] Il s'agirait de préciser ici quelle est la personne responsable de la santé de l'enfant quand les parents sont absents.

4.1 Une maladie connue mais un traitement méconnu

Le paludisme est une maladie connue dans le *fokontany*. A la question de savoir pourquoi les personnes dorment sous moustiquaire, ou encore s'il existe des maladies transmissibles par le moustique, les personnes évoquent à l'unanimité le paludisme. Le moustique transmettant le paludisme est appelé « *moka be* » et est facilement identifiable de par sa grande taille. Le « simple moustique » ne laisse qu'une irritation là où l'autre crée un gonflement à l'endroit piqué. Les moustiques se reproduisent dans la saleté, d'où l'importance de vivre dans un environnement propre pour éviter d'être en contact avec la maladie.

Ainsi, à la différence d'Antsohihy où le lien n'était pas intégré entre le vecteur et la maladie, le moustique est bien identifié ici comme cause du paludisme. Il a été noté que les périodes de pluies (associée dans les esprits à la saison des letchis) connaissent une augmentation des moustiques et donc de la maladie.

> « *On peut reconnaitre que la pluie va arriver si on entend des moustiques dans la maison* » [Homme, 25 ans].

Cependant, si cette maladie existe encore (rares sont les cas identifiés), elle ne constitue pas un problème de santé majeur pour les villageois, car un traitement existe qui guérit rapidement le malade.

> *« Le tazomoka n'est pas un problème ici. C'est une maladie simple car même quand l'enfant tombe malade et qu'il a de la fièvre pendant 3 jours il continue à manger. C'est une maladie facile à soigner, je n'ai jamais vu des enfants faire des rechutes après des prises de médicament »* [Soignant, 45 ans].

Le traitement fait l'objet de confusion : l'Agent communautaire nous dit par exemple que chez l'enfant, la maladie peut être prise en charge grâce à l'Actipal® (amodiaquine et artesunate). Pour les adultes par contre, toujours selon lui, la maladie exige un régime sans sel prescrit par le guérisseur traditionnel. Selon l'instituteur, le traitement contre le paludisme est le paracétamol pour l'enfant et le Cafépar® pour l'adulte (médicament à base caféine et de paracétamol). Ces deux personnes sont justement les deux référents dans le domaine de la santé dans le *fokontany*, il y a donc lieu de s'inquiéter quant à l'identification et la prise en charge du paludisme par ces vendeurs de proximité.

Le paludisme touche particulièrement les enfants qui sont reconnus comme plus exposés à la maladie. Il en découle que la priorité leur est donnée, en cas de manque de moustiquaire. Ce manque de moustiquaire n'a cependant pas été identifié comme un problème. Les nombreuses distributions (pendant les CPN, les vaccinations, etc.) et la mise à disposition, en nombre suffisant, de MID ont au contraire été mises en évidence.

Une majorité des personnes interrogées identifient le *tazomoka* comme une maladie à part entière. Le *tazomoka* est défini comme une fièvre simple, une seule interlocutrice nous a dit

que la maladie tue, en plus de l'Agent communautaire qui vient de recevoir un renforcement de sa formation suite à la préparation de distribution de moustiquaires. Elle se prend en charge facilement et est reconnaissable par les piqûres sur le bras de l'enfant suite à une nuit passée hors de la moustiquaire (en cas d'oubli ou de trop grande chaleur). Selon les dires des interlocuteurs, la maladie apparait directement suite à une piqûre de moustique. Rares sont les personnes qui disent avoir un membre de la famille ou une connaissance ayant souffert de la maladie. Cette maladie ne tue pas dans le *fokontany*, mais il a été reconnu qu'il existe des formes de paludisme qui tuent ailleurs dans le pays.

> *« Il y a des maladies transmises par les moustiques mais ici ce n'est pas très grave, peut-être les moustiques qui tuent ne sont pas encore arrivés jusqu'ici. Les moustiques sont moins agressifs ici, et de toute façon il existe des moyens pour se protéger contre les moustiques* » [Femme, 52 ans].

> « *Le tazomoka est un problème surtout du côté de Tana, selon ce qu'on entend à la radio. Ici ce n'est pas un problème mais ailleurs oui* » [Femme, 60 ans].

Nos interlocuteurs reconnaissent également que le paludisme a été un problème majeur de par le passé, mais ils notent une baisse importante de cas depuis la distribution en masse des MID.

> « *Avant, en 2000, le tazomoka était un problème les gens en avaient peur mais maintenant qu'on a les moustiquaires et les agents de santé on n'en entend plus parler. C'était surtout les enfants qui étaient touchés et s'ils n'étaient pas bien soignés ils mourraient. Si les parents tentaient d'abord l'automédication la maladie s'aggravait et l'enfant pouvait en mourir »* [Homme, 35 ans].

Dans le discours des villageois, le paludisme « qui tue » appartient donc à une époque révolue.

D'autres identifient des symptômes équivalents entre pour le *tazo mahery* et le *tazomoka* et perçoivent ce dernier comme le premier stade de l'évolution de la fièvre : fièvre au départ simple ou *marokoditra*, pouvant mener à des convulsions s'il n'y a pas de prise en charge rapide. Dans ce cas, seul le médecin est en mesure de diagnostiquer l'une ou l'autre maladie.

> « *Les fièvres que provoquent le tazomoka sont simples mais peuvent se compliquer si elles sont mal soignées. Le tazo mahery lui se caractérise par une forte fièvre assez soudaine* » [Femme, 35 ans].

Ainsi, si la maladie semble être connue, on observe une méconnaissance du traitement adéquat (le nom, le coût, la forme) ainsi que du temps d'incubation précédent la maladie.

> « *On peut reconnaitre le* tazomoka *par la piqûre que ça laisse sur la peau. Si on se fait piquer la nuit, 2 ou 3 heures plus tard on tombe malade* » [Jeune femme, 26 ans].

Il existe également un flou concernant le type de moustique transmettant la maladie. Il règne également une incompréhension quant aux réactions différentes face à une piqûre de

« *moka be* » : certains tombent malade et d'autres pas. Une femme l'explique par l'état de santé de la personne avant la piqûre (si elle est en bonne santé elle pourra plus aisément lutter contre la maladie que si elle est en mauvais état), un homme a cité l'immunité contre le paludisme protégeant certains individus.

4.2 Une faible prévalence observée

La faible prévalence ressentie, voire l'inexistence actuelle du paludisme telle qu'identifiée dans le *fokontany* par les interlocuteurs pourrait souligner d'une part le manque d'identification du paludisme due à une mauvaise transmission d'information entre le patient et le médecin (qui ne dit pas « Vous souffrez du paludisme »). D'autre part, elle souligne également une confusion : quand le *tazo mahery* ou les convulsions surviennent, ce n'est jamais associé au paludisme. Pourtant, cette zone relève d'un paludisme pérenne et les chiffres récoltés au CSB de la commune de Tsaravary confirment que cette zone a connu une recrudescence du paludisme au mois de mai et juin dernier. Le développement d'une immunité par les adultes pourrait être une raison de cette banalisation de la maladie et de la rareté des cas observés. Elle n'a cependant été citée qu'une seule fois comme raison de la vulnérabilité des enfants versus les adultes.

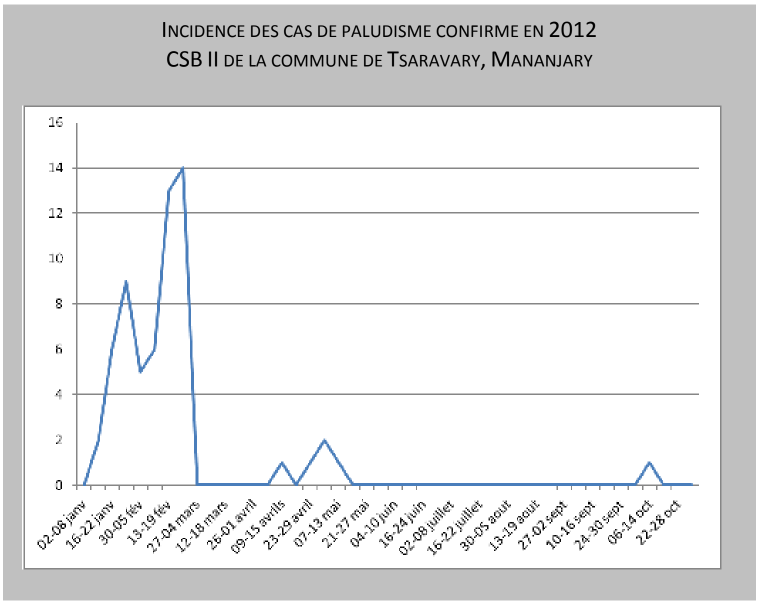

Malgré le petit pic de paludisme observé pour la région en début d'année 2012, peu de personnes répondaient par l'affirmative à la question de savoir si le paludisme a touché des personnes dans le *fokontany* plus particulièrement durant une période de l'année.

Les chiffres à l'échelle nationale vont dans le même sens que les observations des interlocuteurs et des soignants. « Cette diminution significative a été confirmée par une évaluation rapide de l'OMS qui a mis en évidence dans les hôpitaux une diminution de 34% des admissions et de 66% des décès dus au paludisme entre 2001-2004 et 2009. Pendant la même période le recours aux tests de diagnostics rapides ou à la microscopie a augmenté au niveau national passant de 2% à 85% des cas suspects. Le paludisme représentait 19% des consultations externes dans les formations sanitaires en 2001-2005 ; 11% en 2007 et moins de 10% en 2008-2009 »[49]. Du côté des soignants, le paludisme ne présente qu'un pourcentage faible des consultations, du moins en saison sèche : 1 TDR positif pour les 3 premières semaines de novembre pour le CSB de Marovahy, contre 2 pour celui de Tsaravary.

[49] Institut Pasteur de Madagascar, Rapport Sites sentinelles, 2011.

« *Le tazomoka était un gros problème avant 2000. A ce moment-là 80% des malades venaient consulter au CSB pour le tazomoka. Mais aujourd'hui c'est rare* » [Soignant, 40 ans].

Cependant les soignants restent prudents et n'excluent pas que la maladie surgisse plus particulièrement avec l'arrivée de la saison des pluies, dans les mois et semaines à venir.

4.3 Identification de la MID comme raison de la diminution du paludisme

Si la maladie n'est pas perçue comme grave, le comportement de prévention (moustiquaire) est totalement adopté. On aurait pu craindre que ces interprétations entrainent une diminution du recours aux moustiquaires mais, comme nous le verrons au point 5, ce n'est pas le cas.

4.4 Absence de TPI dans les discours des femmes

Malgré la gratuité des TPI et leur distribution officiellement prévue dans les recommandations nationales de lutte contre le paludisme pour la côte est, aucune femme interrogée à ce propos n'a connaissance d'un traitement préventif contre le paludisme spécifiquement offert aux femmes enceintes. Elles citent pourtant un complément en fer, un antiparasite ou encore des vitamines comme médicaments reçus pendant les CPN. Elles notent aussi que pour se protéger contre le paludisme, elles reçoivent la recommandation de dormir sous moustiquaires, mais n'ont jamais entendu parler d'un médicament offert à toute femme enceinte. Une soignante explique cela par la négligence de certains soignants, ou par le fait qu'ils pourraient vendre le traitement. Un soignant nous explique dans ce cadre qu'en cas de rupture de stock de TPI, il conseille aux femmes de s'en fournir par elles-mêmes à la pharmacie de Mananjary. En pharmacie, une dose de TPI s'élève à 1500 Ar, une somme qu'elles préfèrent éviter de dépenser. L'achat du TPI est donc un obstacle à sa prise, les futures mamans préfèreront effectuer la dépense une fois la maladie contractée.

Le taux de fréquentation du CSB au cours de la grossesse pour cette commune n'a pas été évalué. Les observations de non-prise de TPI sont d'autant plus étonnantes que selon un soignant du CSB de Tsaravary, « *90% des femmes de la commune viennent une fois/mois à partir du 5ème mois* ». Il continue en disant que durant la saison des pluies, les femmes enceintes habitant les *fokontany* du sud de la commune dont la route est bloquée par les crues de la rivière Mananjary, peinent à accéder au CSB. Mais malgré cet obstacle, les femmes s'organisent pour y assister.

A nouveau nous pouvons émettre l'hypothèse qu'il y a un manque de communication entre le soignant et la soignée, amenant l'ignorance de cette dernière concernant les médicaments pris et leur utilité. Les ruptures de stocks (apparemment un problème fréquent) pourraient être un second obstacle à la prise de TPI.

5.1. Mananjary : un terrain favorable à la sensibilisation

5.1.1 Utilisation assidue de la moustiquaire

Une majorité d'interlocuteurs ont affirmé dormir toutes les nuits durant toute l'année sous MID. Nous n'avons identifié aucune maison où une moustiquaire n'était pas attachée. La première raison avancée est la protection contre les insectes de tous genres (puces, tiques, moustiques) et leurs piqûres, qui laissent des traces particulièrement sur les bras des enfants. Le produit insecticide, par ailleurs appelé *fanafody* ou médicament est considéré comme efficace pour l'élimination des insectes. La tranquillité qu'apporte une nuit sous moustiquaire a été souvent évoquée. La seconde raison est la protection contre le paludisme. Enfin, la protection contre le froid en hiver a également été avancée. Les raisons évoquées pour la non utilisation est la chaleur et la paresse, ainsi que l'odeur trop forte du produit imprégné.

Avant la première distribution gratuite en 2009, nombreux étaient les ménages qui opéraient des dépenses pour se procurer les moustiquaires : entre 5000 et 10 000 Ar pour une moustiquaire cousue main, 3000 Ar pour une moustiquaire déjà fabriquée. L'avantage souligné des moustiquaires cousues à la main est la possible adaptation de la taille. Depuis que l'accès aux moustiquaires est gratuit via les distributions, rares sont les ménages qui opèrent des dépenses dans ce domaine. A Antsohihy, une majorité des ménages détenant des moustiquaires possédaient des « 4 portes », à Mananjary nous n'avons observé que des MID.

5.1.2 L'adoption de la moustiquaire : un fombandrazana

Dans ce *fokontany*, la moustiquaire fait partie du trousseau de mariage et de naissance, elle appartient à la tradition familiale de la région. A l'époque (qui a été datée à il y a une trentaine d'années) où les individus n'entendaient pas encore parler de paludisme, la moustiquaire était cousue et utilisée pour le prestige qu'elle offrait aux jeunes mariés et futurs parents. A la naissance de l'enfant, la moustiquaire était requise pour le protéger contre les piqûres d'insectes. Aujourd'hui cette habitude est restée et le mariage ou la naissance d'un enfant sont des moments clés dans l'utilisation de la moustiquaire et depuis la gratuité de l'objet, le niveau de richesse n'a aujourd'hui plus rien à voir avec le fait d'en disposer ou non. Les couples rencontrés se souviennent de leur mariage comme le moment de début d'utilisation de la moustiquaire.

> *« La moustiquaire fait partie des objets à posséder comme le matelas, les coussins, les ustensiles de cuisine pour les jeunes mariés (…) Avant c'était juste une mode, maintenant c'est un médicament, c'est imprégné. C'est un médicament pour la santé* » [Femme, 52 ans].

Selon un de nos interlocuteurs, ce message est divulgué par son église, qui met en parallèle la capacité à être un « bon père de famille » en prenant soin de celle-ci et le fait de doter sa maison d'une moustiquaire.

« C'est le rôle d'un homme quand il va se marier d'équiper la maison, quand il se considère comme un adulte, il n'est plus un enfant qui se fiche de dormir ou non sous moustiquaire. La moustiquaire fait partie des équipements nécessaires pour la maison. À l'église on nous explique que le mariage c'est sacré et que comme il deviendra adulte, l'homme doit prendre soin de la maison et la moustiquaire fait partie de ces choses à avoir. Avant c'était une question de mode d'avoir la moustiquaire. Maintenant c'est un médicament, c'est pour la santé » [Homme, 35 ans].

Sur la dizaine de personnes interrogées à la sortie de la distribution de moustiquaires, deux hommes nous ont dit que c'était leur première moustiquaire : le premier venait de se marier, le second venait tout juste d'avoir un enfant.

5.1.3 Préférences en matière de moustiquaires

En ce qui concerne les préférences des moustiquaires, plusieurs aspects sont pris en compte par les utilisateurs : la couleur d'abord, la taille des mailles, la qualité du tissu, le produit dont elles sont imprégnées et enfin la taille de la moustiquaire.

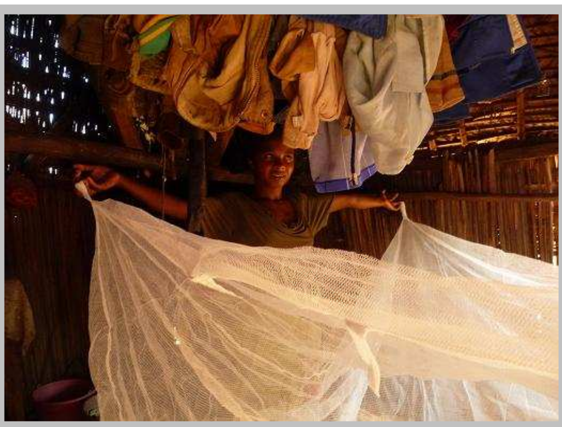

DÉCOUVERTE DE LA MOUSTIQUAIRE

La couleur blanche a globalement été évaluée comme trop salissante bien que certaines personnes aient mis en évidence le côté lumineux des moustiquaires blanches (versus les bleues reçues en 2009). La taille des mailles est un autre aspect essentiel selon eux, plus elles sont petites, plus la sensation d'être protégé sera grande. La qualité des tissus est également essentielle : fluide et léger, ce sont deux caractéristiques dont doivent être dotés ces objets incontournables du quotidien. Le produit dont elles sont imprégnées a la réputation de donner des maux de têtes et des rhumes. Mais malgré ce désavantage, la MID, considérée comme un réel médicament, est préférée aux moustiquaires cousues. Enfin, la taille est importante également. Il nous a été dit que les ménages peuvent dormir jusqu'à 5 personnes sous une seule moustiquaire. Les jeunes enfants jusqu'à 5 ans dorment avec leurs parents. Ensuite, les maisons sont souvent de petites tailles et ne permettent pas aux ménages nombreux de dormir, comme selon la recommandation, à 3 sous moustiquaires, ce qui signifierait parfois qu'il y ait, dans une même maison, 3 moustiquaires.

5.1.4 Autres moyens de protection contre les piqûres de moustique

Au-delà de l'utilisation des moustiquaires, d'autres moyens nous ont été donnés pour intensifier la lutte contre les moustiques dans le *fokontany* : remblayer les étangs, nettoyer les buissons, creuser des trous pour rassembler les déchets au même endroit. Pour chasser les moustiques des habitations, des fleurs d'orangers sont brulées. Un interlocuteur a noté également que le fait de cuisiner dans les habitations éloigne les moustiques.

Les interlocuteurs expliquent donc que l'adoption de la moustiquaire au quotidien trouve sa source tout d'abord dans le nombre important de moustiques et l'observation faite de l'efficacité des MID sur l'éloignement des moustiques. Ensuite, ils sont familiers avec l'utilisation de cet objet depuis plusieurs dizaines d'années, au vu de son importance au sein de la tradition d'équipement rythmant les grandes étapes de la vie d'une famille.

5.2 Déroulement de la distribution

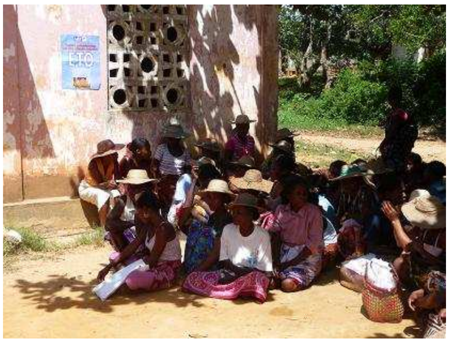

La distribution pour l'ensemble de la région était organisée en une semaine. Le message diffusé à la radio annonçant le déroulement de l'évènement et la gratuité des moustiquaires était réalisé en partie en langue locale, en partie en malgache officiel. Il a été observé que ce sont majoritairement les femmes qui y ont assisté[50]. Le *fokontany* investigué fut le premier de la commune à recevoir les moustiquaires. Les villageois étaient attendus au petit matin dans le chef-lieu de la commune, situé à une heure trente de marche (7km), pour le discours d'ouverture de la semaine de distribution par le maire, suivi d'une courte sensibilisation et enfin la distribution. Une moustiquaire était déballée en démonstration, afin d'illustrer les messages concernant son utilisation. Une équipe de 6 personnes étaient mobilisée : 3 anciens Agents communautaires (un chef de site, un distributeur et un contrôleur), 2 Agents communautaires et un soignant du CSB pour superviser. Concrètement, 2 filtres étaient mis en place pour éviter les abus et assurer une distribution fidèle aux chiffres récoltés quelques mois plus tôt durant le recensement : un à l'entrée du bâtiment, l'autre à la sortie. Une moustiquaire était prévue pour 3 personnes.

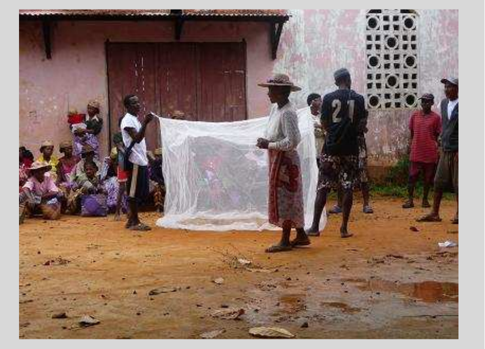

5.2.1 Evaluation rapide des messages retenus

Dans le cas du *fokontany* investigué, la majorité des participants est arrivée après la sensibilisation. Une rapide évaluation des messages retenus démontre une méconnaissance quant à la durée d'imprégnation des moustiquaires (malgré l'inscription en grand du chiffre 5 sur le paquet !), du mode de mise en place (temps d'aération avant de l'attacher dans les habitations). En revanche, la plus grande vulnérabilité des enfants a été retenue, ainsi que l'importance de se protéger contre le paludisme, particulièrement dans le district de Mananjary. L'interdiction de vente a également été notée. Les retardataires s'appuient sur les savoirs déjà acquis lors de la distribution de 2009 (surtout concernant l'entretien de la moustiquaire) et comptent sur leur entourage pour pallier au manque d'information.

Le degré de satisfaction a été rapidement sondé également. Quatre critiques ont été formulées. Une critique concernant l'organisation de la distribution et trois concernant la qualité de la moustiquaire. La première remet en cause la gratuité, officiellement diffusée, des moustiquaires. Selon les dires des interlocuteurs, l'équipe d'Agents communautaires,

[50] Cela s'explique notamment par le fait qu'en cette période de l'année, les travaux aux champs ne requièrent habituellement pas l'aide des femmes.

avec l'appui des autorités administratives locales, a lancé durant la sensibilisation un appel de mobilisation des villageois à participer à leurs frais de défraiement, à la hauteur de 300 Ar/moustiquaire reçue. Une réaction s'est apparemment rapidement fait entendre : la gratuité ayant été affirmée officiellement par la radio et les banderoles, une majorité des personnes ont refusé de se soumettre à cette demande. Une contribution de 500 Ar avait apparemment également été demandée en 2009, mais celle-ci avait été officialisée, les personnes n'ont alors pas remis en cause cette participation. Durant les quelques jours qui ont suivi cet incident, c'est le seul message qui semblait être retenu.

> UN ROLE INATTENDU A ETE ATTRIBUE A NOTRE EQUIPE
>
> Il nous a été raconté qu'à la vue de notre présence durant la distribution, les Agents communautaires auraient arrêté la récolte de 300 Ar, présentée avant notre arrivée comme étant une participation aux frais causés par l'engagement des Agents communautaires dans le processus de distribution. Ceux-ci ont cru voir en nous des agents chargés par les bailleurs de la supervision de la distribution des moustiquaires et ont eu peur des répercussions de cette proposition ressentie comme « obligatoire » de participation à leurs frais.

Concernant la qualité de la moustiquaire, les bénéficiaires trouvent les mailles trop grandes, le tissu trop rigide et la taille de la moustiquaire trop petite. Un autre incident est venu ponctuer la semaine de distribution : des MID issues de la distribution ont été trouvées en vente sur le marché et ce malgré la précaution prise lors de la distribution d'ouvrir préalablement l'emballage et l'interdiction de vente spécifiquement précisée durant la sensibilisation. Le revendeur était originaire du *fokontany* investigué, selon les rumeurs circulant dans l'ensemble de la commune. Dès lors, les moustiquaires étaient retirées de leurs sachets pour la distribution, causant à nouveau des réactions négatives concernant le contact du produit d'imprégnation sur la peau, durant les minutes ou heures de marches pour rejoindre le lien de vie du bénéficiaire. En outre, les villageois ont ainsi été privés des informations inscrites sur les sachets (si tant est qu'ils tiennent compte des informations qui y sont écrites).

5.2.2 Usages faits des anciennes moustiquaires

Les anciennes moustiquaires seront nettoyées et gardées pour les couches des invités. Quelques jours après la distribution de MID, certains villageois affirmaient qu'ils allaient garder les anciennes moustiquaires suspendues sur leurs lits, jusqu'à ce qu'elles soient vraiment trop usées, préservant les autres dans le sachet dans lequel ils les ont réceptionné (pour des raisons de non satisfaction des nouvelles ou de volonté de s'assurer d'avoir encore d'autres moustiquaires de rechange). Une personne nous a dit qu'elle utilisera les moustiquaires trop abimées et trouées pour pêcher.

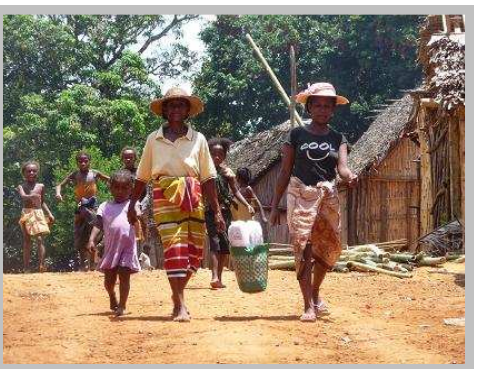

RETOUR AU VILLAGE APRES LA DISTRIBUTION

6.1 La moustiquaire comme partie intégrante du décor

Mananjary (et sans doute plus largement la côte est) est donc un terrain propice à l'assimilation des campagnes de sensibilisation contre le paludisme pour différentes raisons : la place gagnée par la moustiquaire dans l'équipement requis à l'occasion de l'installation d'un jeune couple et la naissance d'un enfant, couplée d'une présence en grand nombre de moustiques et donc de paludisme.

6.2 Paludisme : un problème réellement résolu ?

On l'a vu, les chiffres de l'OMS corroborent les impressions des interlocuteurs comme des soignants, confirmant une baisse du paludisme depuis la mise en place des stratégies de lutte. Cependant, à long terme la minimisation de la maladie perçue comme simple à guérir et rare pourrait être néfaste dans la prise en charge de celle-ci, notamment au vu de la nouvelle saison des pluies qui vient de commencer.

6.3 Blocages à l'accès au traitement

A l'instar des autres zones étudiées, l'automédication est le réflexe premier adopté en cas d'apparition de fièvre chez l'enfant. La mise en place des Agents communautaires permet aujourd'hui déjà d'éviter, à cette partie de la population la plus vulnérable, de tomber dans des épisodes de maladies trop fréquents. Par ailleurs, le problème de l'accès aux soins reste existant, en cas de maladie graves pour les moins de 5 ans, et pour toutes les maladies pour le reste de la population, confrontés aux obstacles d'accès géographique et financier.

7.1 Renforcement du lien entre convulsions, fortes températures et paludisme

Au vu de l'utilisation systématique de la moustiquaire par une grande majorité de ménages tout au long de l'année, il n'est plus nécessaire que le travail de sensibilisation concentre toute son énergie sur la nécessité de l'adoption de cette pratique de prévention. Il s'agirait plutôt de renforcer les lacunes particulières à cette région : méconnaissance du contexte de maladie (nom de la maladie et traitement) et reconnaissance des symptômes liés au paludisme. Car en effet, la diminution des cas de paludisme ressentie par les villageois a entrainé une perte de (re)connaissance de ces symptômes, résumés aujourd'hui par de simples fièvres facilement guérissables. Les convulsions et fièvres fortes ne sont pas associées au paludisme, mais renvoient à ce qu'ils appellent *tazo mahery* qui est pour eux une maladie à part entière.

Cette observation, additionnée au manque d'informations transmises par les médecins une fois le diagnostic réalisé, entraine une minimisation du paludisme, ainsi qu'une méconnaissance concernant les symptômes spécifiques de la maladie. Cependant, à la différence d'Antsohihy où cette méconnaissance entraine des comportements laxistes en ce qui concerne l'utilisation de moustiquaire, ici l'utilisation reste un réflexe au quotidien.

Afin d'accompagner les malades dans l'identification des symptômes du paludisme, il nous semble important de renforcer le lien entre les convulsions et la forte température, cités comme deux problèmes de santé fréquemment encourus dans le *fokontany*, avec le paludisme, qui pourrait en être la cause.

7.2 Renforcement de la gestion des soins à l'échelle communautaire

Avec la mise en évidence des dynamiques locales récurrentes de revente de médicaments dans les *fokontany* par des particuliers et le recours aux Agents communautaires pour la prise en charge des enfants de moins de 5 ans, il nous a été prouvé que les services de proximité (officiels ou clandestins) sont choisis en premier recours dans le parcours de recherche de soin des malades dans cette région. Dans cette optique, la mise en place des Agents de santé communautaire depuis 2011, notamment selon l'approche participative « *Kaominina mendrika salama* » et la prise en charge intégrée dont ils sont responsables (PCIMEc) constituent un grand point d'avancement dans le renforcement de la capacité locale, particulièrement adéquat à cette région où il nous a été répété systématiquement « *que les malades préfèrent d'abord chercher des soins dans leur fokontany avant de se référer au docteur* ».

Parallèlement, nous avons observé également l'importance du poids de l'entourage dans l'orientation des itinéraires thérapeutiques et plus particulièrement le rôle des femmes dans la prise en charge de la santé de leurs enfants. Dans ce cadre, les CPN ont été citées comme

le second vecteur de transmission de connaissances concernant la santé (notamment pour le paludisme, via la distribution de moustiquaires durant les CPN).

Malgré les efforts cumulés, nous avons pourtant observé que ces recours de proximité contiennent de réelles lacunes quant à la prise en charge et la reconnaissance des maladies et traitements adéquats offerts par ces soignants « improvisés ». L'idée d'aggravation de la maladie due à un traitement inadéquat a en effet été citée par une majorité des soignants enquêtés comme problème fréquent découlant de la pratique d'automédication. Ces soignants sont pourtant en première ligne dans le domaine de la prise en charge de la santé mais ils ne sont que peu outillés. Ainsi par exemple, le principal revendeur de médicament dans le *fokontany* ne possède pas de TDR, ni de thermomètre et n'est donc pas en mesure de prendre en charge le paludisme conformément aux recommandations.

Ayant connaissance de cette réalité « clandestine » et ayant pris note de la récurrence du phénomène, il serait nécessaire d'appuyer ces « relais communautaires » (le dispensateur de médicaments, les mères, etc.) afin de renforcer les connaissances de ces personnes ressources, par des formations et un soutien matériel. Ces personnes ressources, cumulées aux Agents communautaires responsables uniquement de la prise en charge des enfants de moins de 5 ans, pourraient constituer des relais afin d'éviter une prise en charge retardée ou inadéquate des maladies « simples » à prendre en charge.

7.3. Mise en évidence des mécanismes de gratuité des soins existants

Une interlocutrice a évalué les couts liés au traitement anti palu à 10 000 Ar, celui de la fièvre forte à 26 000 Ar. La diarrhée a été considérée comme une maladie chère à prendre en charge. A la question de savoir si certains traitements sont gratuits, tous ont répondu qu'il n'y avait aucun traitement disponible gratuitement.

> « *Il existe des médicaments contre le tazomoka ? » « Oui mais ça dépend de l'argent. Si on n'a pas d'argent on se protègera avec les moyens qu'on a (ici par exemple on frotte des oignons sur les plaies). C'est seulement ceux qui ont de l'argent qui peuvent prendre le médicament chez le docteur* » [Jeune homme, 17 ans].

Pourtant il existe plus d'un traitement gratuit : au-delà de la problématique qui nous intéresse plus particulièrement ici à savoir le paludisme, la tuberculose ou encore la malnutrition aiguë sévère est également prise en charge gratuitement dans les CRENAS[51] (au sein des CSB)[52] et d'autres traitements sont délivrés gratuitement. Une politique de recouvrement des coûts dans le secteur de la santé concernant les médicaments et les consommables médicaux et portant le nom de FANOME[53] a été réinstaurée en 2004 par le Ministère de la santé et du Planning familial, mettant en place un système d'exemption pour

[51] Centre de récupération nutritionnelle
[52] Activité du ministère de la santé appuyée par l'Unicef.
[53] Financement pour l'Approvisionnement Non stop en Médicaments Essentiels.

les plus pauvres (étiquetés comme « indigents » en fonction de certains critères)[54]. Cette politique cherche à augmenter l'utilisation des services de santé par les pauvres, par le biais des fonds d'équité. Il existe donc des mécanismes facilitant l'accès aux soins des plus pauvres, qui mériteraient d'être mieux connus et renforcés afin de briser cette image de cherté des soins de santé. Il serait intéressant de confirmer la réelle gratuité des traitements sur le terrain : rupture de stock des traitements entrainant la nécessité d'achat du médicament en pharmacie ou « paiements informel »[55] [O. de SARDAN et RIDDE, 2012 : 23] soutirés par les professionnels de la santé[56] aux patients pour la vente illégale de médicaments.

7.4 Prise en compte des spécificités culturelles pour les opérations de distribution

Une spécificité à prendre en compte en ce qui concerne les distributions de moustiquaires sont les tabous (*fady)* et habitudes concernant l'organisation spatiale des lits. Quand l'enfant fille ou garçon aura atteint l'âge de 6 ans, il quittera le lit de ses parents pour une autre couche, toujours dans l'habitation familiale. Une fois qu'il atteindra l'âge de 14 ans il aménagera dans sa propre maison. Dès l'âge de 9 ans, un frère et une sœur ne peuvent plus partager un même lit. Deux sœurs ou deux frères peuvent par contre dormir sur la même couche jusqu'à ce que l'un soit marié. Une mère célibataire peut dormir avec son fils jusqu'à ce qu'il ait 6 ans, avec sa fille jusqu'à ce qu'elle ait 14 ans.

7.5 Rendre « attractifs » les messages de sensibilisation

Les préférences quant au canal de diffusion de message sont la radio en premier lieu, suivi rapidement par les campagnes au sein des *fokontany*. Pour cette dernière, les échanges particuliers entre les Agents communautaires et les villageois, circulant de porte à porte sont préférés aux assemblées de masse. La langue préférée pour ces messages est la langue locale pour la majorité. Un interlocuteur souligne toutefois qu'ils sont familiers avec le malgache officiel car beaucoup d'émissions radio se déroulent dans cette langue, sans que ce soit un frein à la compréhension. Il nuance cependant qu'il se sentira d'avantage concerné par un message traitant de Mananjary, dans la langue locale.

Les informations inscrites en français et en anglais sur les emballages de moustiquaires ne sont pas regardées. Les bénéficiaires auront recours à l'AC ou à l'entourage pour connaitre les instructions concernant la mise en place de la moustiquaire plutôt que de lire les inscriptions sur le sachet. L'oralité des messages sera donc préférée aux messages écrits.

---

[54] Pour plus d'informations sur les fonds d'équité à Madagascar, voir le rapport : PONCIN X. et LE MENTEC R., 2009, « Politiques d'exemptions pour les soins de santé à Madagascar : le cas des fonds d'équité », AEDES. [http://performancebasedfinancing.files.wordpress.com/2009/10/rapport-final-partie-fonds-dequite.pdf]

[55] O. DE SARDAN J.P., RIDDE V., 2012, «L'exemption de paiement des soins au Burkina Faso, Mali et Niger. Les contradictions des politiques publiques », Afrique contemporaine, n°243, 23 p.

[56] Personnels de santé des formations sanitaires privées, publiques mais également les agents de santé communautaires (AC)

Concernant les moustiquaires enfin, il serait utile de prendre en compte les critères de confort tels qu'édictés par les villageois. En effet, ceux-ci passent le tiers de leur vie sous moustiquaire (toutes les nuits durant toute la vie !). Il est donc essentiel que l'objet leur convienne.

IV. LEÇONS APPRISES

Des perceptions floues du paludisme et des fièvres

Les enquêtes réalisées dans les quatre zones d'étude soulignent combien les perceptions populaires du « paludisme » (*tazomoka*) et plus généralement des fièvres (*tazo*) sont floues. On distingue au moins trois types de fièvre : la fièvre simple (*tazo*), le paludisme (*tazomoka*) et la fièvre forte, souvent associée aux convulsions (*tazo mahery*). Dans les représentations, ces trois types de fièvres ne constituent pas des catégories étanches : *tazo* peut devenir *tazomoka* si elle n'est pas soignée, qui peut se transformer en *tazo mahery*. Il apparaît que le lien entre le vecteur, le moustique, et la maladie n'est pas clair. En effet, à la question : « Connaissez-vous des maladies transmises par le moustique ? », les réponses citent toujours *tazomoka*. Cependant, il apparaît que dans les perceptions, *tazomoka* n'est pas uniquement dû aux piqûres de moustique, mais aussi au manque d'hygiène, à la fatigue, au fait de manger certains fruits. Ces interprétations des causes de la maladie peuvent trouver leurs sources dans la saisonnalité du paludisme (l'apparition des moustiques coïncide avec l'arrivée de certains fruits), ou dans les campagnes de prévention du paludisme, qui mettent en avant la nécessité de débroussailler et de nettoyer les alentours des maisons.

Quel que soit le faciès épidémiologique de la zone étudiée, le paludisme n'est pas perçu par la population comme un problème de santé grave ou préoccupant. Ainsi, l'épidémie survenue à Antsohihy au printemps 2012 a été perçue comme un problème de santé grave, du fait du nombre de décès constatés, mais cette gravité a induit qu'il ne pouvait pas s'agir du paludisme. Par ailleurs, les femmes enceintes et les enfants de moins de 5 ans sont perçus comme étant particulièrement vulnérables face au paludisme. Cette perception est liée aux campagnes de sensibilisation et peut avoir pour conséquence que les hommes et les couples âgés sans enfants se sentent parfois moins concernés par le risque d'avoir le paludisme alors que dans la plupart des régions de Madagascar l'ensemble de la population est à risque, sans restriction d'âge ou de sexe. La plus grande vulnérabilité perçue des femmes enceintes et des enfants n'est pas forcément prise en compte dans les comportements de prévention. Ainsi, si ce sont majoritairement les femmes enceintes et les enfants de moins de 5 ans qui dorment sous moustiquaires dans trois zones d'étude, à Antsohihy, la priorité est donnée au couple et à la femme enceinte.

Les raisons du retard aux soins et du faible recours aux formations sanitaires

Le principal problème dans la prise en charge du paludisme reste le faible recours aux formations sanitaires et le retard aux soins. Ce retard relève de différentes causes :

- Les pratiques usuelles en cas de maladie et en cas de fièvre : tout itinéraire de soins commence par l'automédication, impliquant souvent le recours au revendeur local de médicaments (épicerie, vendeur ambulant). Ce recours peut se faire simultanément à l'automédication sur base du savoir-faire légué par les ancêtres (inhalations et tisanes). Ensuite, il y aura éventuellement un recours (et parfois de manière simultanée) au guérisseur traditionnel et aux Agents communautaires. Le recours aux structures de santé apparaît toujours comme l'ultime recours, lorsque les autres se sont révélés inefficaces.

- Les contraintes et choix financiers : le déplacement vers la structure de santé, la consultation, les traitements (ceux-ci sont supposés être donnés gratuitement en cas

de paludisme, mais la population ne le sait pas) impliquent des dépenses que les familles ne sont pas prêtes à supporter, d'autant plus que la santé n'apparaît pas comme un secteur prioritaire d'investissement. On observe ainsi une évaluation et une hiérarchisation des risques (sanitaires, financiers, sociaux) en cas de maladie, qui mènent à choisir l'attente d'une guérison spontanée qui épargnerait la dépense non souhaitée ou non réalisable ou le recours à des traitements palliatifs supposés moins chers.

- L'éloignement des structures de santé.

- Le manque d'information de la population sur le paludisme : le paludisme est perçu comme une maladie dont on ne meurt pas, qui n'a pas de caractère de gravité, et dont on guérit spontanément ou en ayant recours à des palliatifs.

Attitudes à l'égard des maladies et des traitements médicaux, et relations soignants-soignés

L'analyse des récits concernant les événements de maladie met en évidence le faible niveau de connaissances que les patients ont de leur maladie et des médicaments qu'ils ont pris. Sont ici en cause d'une part le manque de communication entre soignants et soignés, les soignants ayant une perception des patients comme des personnes peu aptes à comprendre leur maladie, et d'autre part le manque d'intérêt des patients à connaître le nom des maladies et des traitements prescrits. L'espace de la consultation médicale ne semble pas perçu par les malades comme un espace où ils peuvent poser des questions sur leur maladie ou ses traitements. Il semble notamment que les patients n'osent pas poser de question par crainte de faire perdre du temps au soignant, dans un contexte où il doit prendre en charge un grand nombre de patients.

Par ailleurs, les individus expriment une grande confiance à l'égard des médicaments donnés par le médecin. Cependant, ils font preuve d'un certain laxisme dans la prise des traitements. En effet, à partir du moment où le malade se sent mieux, il arrête la prise des médicaments (ACT ou traitements donnés pour une autre maladie) lorsque le traitement est administré sans supervision médicale[57].

Les perceptions de l'efficacité des traitements diffèrent d'une zone à l'autre. Alors qu'à Fianarantsoa et Mananjary, les traitements par injections sont considérés comme plus efficaces et aux effets plus rapides, à Moramanga, ce sont les comprimés qui sont considérés comme les plus efficaces (en dépit des effets secondaires liés à la prise de comprimés), et à Antsohihy, on préfère les comprimés aux injections en raison de la peur qu'elles inspirent et des infections qu'elles peuvent provoquer.

L'accès aux ACT : des différences entre les quatre zones

Concernant l'accès aux ACT, des différences entre les quatre zones d'étude en termes de disponibilité et de gratuité ont été mises en évidence. Le rôle des Agents communautaires dans l'accès aux ACT varie d'une zone à l'autre. A Antsohihy, les Agents communautaires disposaient d'ACT qu'ils donnent gratuitement aux malades. A Fianarantsoa, les ACT dont ils disposaient étaient périmés et donc inutilisables. A Moramanga, les Agents communautaires ne disposaient pas de médicaments, ni de TDR. A Mananjary, ils disposaient d'Actipal® qu'ils vendaient 100 Ar le traitement complet pour un enfant. Les enquêtes montrent sur les

[57] Cette attitude a été observée à Mananjary et à Antsohihy, mais la question n'a pas été investiguée à Moramanga et Fianarantsoa.

quatre zones que les Agents communautaires sont généralement perçus par la population comme des acteurs de santé qu'ils consultent en cas de fièvre ou de maladie qui persistent après l'automédication, mais ceux-ci n'ont pas toujours les connaissances ni les médicaments pour les prendre en charge. Leur rôle se limite parfois à orienter le malade vers une structure sanitaire. Et ils sont en demande de formation, de reconnaissance, notamment financière, et de responsabilité.

Le TPI : méconnaissances des femmes et indisponibilité des traitements

Dans les quatre zones étudiées, rares sont les femmes qui affirment avoir pris le traitement préventif contre le paludisme pendant leur grossesse (elles citent pourtant le complément en fer et les vitamines). Plusieurs raisons peuvent expliquer cette méconnaissance du TPI. Soit elles ont pris des médicaments sans qu'on leur explique de quoi il s'agissait et sans qu'elles s'en souviennent (quelques femmes ne se souvenaient pas avoir pris des médicaments, mais en leur demandant d'y réfléchir, elles se sont souvenues avoir pris des comprimés qu'elles ont avalés sans se poser de questions car « tout ce que donne le médecin, c'est bien »), soit les soignants n'ont pas donné le TPI (des ruptures de stocks ont été rapportées). Quoiqu'il en soit, il est nécessaire d'approfondir cette problématique et de faire des recherches complémentaires (enquêtes auprès des soignants et observations de CPN) afin de comprendre pourquoi le TPI n'est pas donné systématiquement aux femmes enceintes, et pourquoi on ne leur explique pas de quoi il s'agit quand il leur est administré.

La MID : une utilisation variable, rarement en lien avec la protection contre le paludisme

Les attitudes vis-à-vis de l'utilisation de la moustiquaire sont variées selon les zones d'enquête. Cette variabilité s'explique par la présence plus ou moins marquée des moustiques et, dans une moindre mesure, par l'importance du paludisme dans la région considérée. En effet, l'utilisation des moustiquaires est toujours justifiée en premier lieu par la gêne occasionnée par les moustiques. La protection contre le paludisme est citée comme deuxième raison d'utilisation de la MID à Mananjary seulement. Dans les trois autres zones, la protection contre le paludisme n'intervient pas en priorité dans les raisons d'utilisation de la MID. Outre la présence des moustiques, il apparaît que de nombreux facteurs favorisent ou au contraire limitent l'utilisation des moustiquaires : la température (chaleur, froid), le vent, la luminosité, la préservation de l'intimité du couple. Le nombre et les caractéristiques des moustiquaires détenues influencent également leur utilisation. Dans les trois zones où la MID fait partie des stratégies de lutte contre le paludisme, lorsque le nombre de moustiquaires est insuffisant dans un ménage, ce sont les enfants de moins de 5 ans et les femmes enceintes qui dorment en priorité sous la moustiquaire. Une attention est également accordée au tissu, à la taille, à la forme, à la couleur et à la grandeur des mailles. En général, sont préférées les moustiquaires au tissu souple et fin, rectangles et suffisamment grandes pour dormir à plusieurs, bleues ou colorées (le blanc étant trop salissant), avec des mailles fines.

Les discours et les observations montrent que le principal message de sensibilisation retenu est qu'il faut dormir sous moustiquaire. La raison de cette nécessité (se protéger contre le paludisme) n'est pas toujours intégrée. Les consignes d'utilisation et de lavage ont généralement été entendues, certaines ont été retenues (suspendre la MID avant de l'utiliser) mais elles ne sont pas toujours appliquées (certains suspendent la MID dans la maison au lieu de l'aérer à l'extérieur, d'autres la font sécher au soleil alors qu'il est indiqué de la faire sécher à l'ombre…). La durée d'efficacité du produit n'est pas connue. Ces

exemples montrent que la multiplicité des discours nuit à une information de qualité et brouillent les messages.

La CAID : de nombreux désagréments et une efficacité rarement perçue

Dans les deux zones d'étude où elle fait partie des stratégies de lutte (Moramanga, Fianarantsoa), la CAID (plus connue par la population sous le nom de DDT) est loin de faire l'unanimité et essuie de nombreuses critiques. D'une part, le lien entre la CAID et le moustique (et à fortiori la lutte contre le paludisme) n'est pas fait. La CAID est en effet perçue comme un moyen de lutter contre les petits insectes (puces et cafards principalement), mais pas forcément contre les moustiques. D'autre part, de nombreux désagréments dus aux aspersions ont été rapportés : mauvaises odeurs, maux de tête, dégradations des murs, décès de poules… Enfin, le produit aspergé est souvent perçu comme peu efficace (voire pas efficace du tout) ou efficace mais pendant un temps très limité. L'inefficacité du produit serait associée à sa couleur trop claire et à son odeur trop faible qui renseigneraient sur le fait que le produit aurait été mélangé à de l'eau, et qu'il aurait été l'objet d'une revente illégale. Certains enquêtés ont témoigné avoir pu acheter de l'insecticide aux équipes d'aspergeurs. Si les insatisfactions à l'égard de la CAID sont générales, rares sont les personnes qui refusent l'aspersion à leur domicile, car ces campagnes émanent des autorités, et les populations ne veulent pas se distinguer par des comportements récalcitrants.

V. RECOMMANDATIONS/PRECONISATIONS

Mieux cibler les supports d'informations et les messages : renforcer les informations concernant les conduites à tenir en cas de fièvre

La multiplicité des messages de prévention, de leurs objectifs et de leurs supports nuit à la diffusion d'une information claire et de qualité. Les populations sont soumises à de nombreux messages émanant de structures nationales et d'ONG diverses, dans le domaine de la santé, du planning familial, de l'assainissement, etc. Malheureusement, ces messages s'accumulent ou se remplacent les uns les autres, ils entrent parfois en concurrence et brouillent l'information. Il paraît donc essentiel que les campagnes d'information et de prévention sur le paludisme soient menées en synergie avec les autres campagnes.

Dans le domaine du paludisme, il est nécessaire de cibler la ou les informations essentielles. Délivrer des informations plurielles et complexes est contre-productif. Par exemple, certains enquêtés savent que le paludisme est transmis par le moustique femelle. Mais cette information ne leur pas utile pour s'en protéger. Il s'agit donc de mieux cibler le contenu des messages à transmettre. A ce titre, il semble essentiel de renforcer les informations sur les conduites à tenir en cas de fièvre, auprès de la population et des acteurs impliqués dans la santé, comme les Agents communautaires.

Il serait également nécessaire de renforcer les connaissances des populations sur la gratuité des traitements contre le paludisme.

Il serait nécessaire de revoir les modalités de réalisation des campagnes d'IEC. Il apparaît que quand l'IEC est faite par les agents de santé, avant la consultation ou en début de consultation médicale, elle est souvent faite de manière trop rapide et elle s'avère inefficace. Il faudrait par conséquent mieux cibler les supports de l'IEC, les agents en charge de la transmettre, et les moments et lieux les mieux adaptés à la compréhension et l'appropriation, par la population, des informations nécessaires.

Prendre en compte les réalités locales et les interdits

Les messages de sensibilisation et de prévention doivent également être adaptés aux réalités locales. Une stratégie et des supports définis à l'échelle nationale ne pourraient être adaptés à l'ensemble du pays. Ainsi, dans une zone comme Mananjary, il est inutile d'insister sur la nécessité de dormir sous moustiquaire puisque l'utilisation de la MID est déjà intégrée dans les habitudes conjugales et familiales. De la même manière, dans les régions et les périodes chaudes, lorsque la population reste à l'extérieur jusque tard le soir, comment justifier l'utilisation de la moustiquaire pendant la nuit seulement alors que les individus sont en contact avec les moustiques toute la soirée ? Ne faudrait-il pas développer d'autres moyens de prévention plus adaptés que la moustiquaire à cette période de la journée ?

Par ailleurs, il est essentiel que les campagnes de distribution de MID prennent en compte les interdits concernant le fait de dormir ensemble (entre frères et sœurs, entre frères, entre sœurs, selon leur âge). Donner une MID pour trois personnes est souvent insuffisant, et il est fréquent que toute la famille ne puisse pas dormir sous moustiquaire.

Par conséquent, afin de renforcer l'efficacité des messages de prévention, la prise en compte des réalités locales et des modes de communication locaux est recommandée, ce qui va à l'encontre d'une uniformisation des messages à l'échelle du pays.

S'appuyer sur les interlocuteurs et modes de communication locaux

Pour s'assurer que les messages de prévention soient transmis de manière à ce que le plus grand nombre ait accès à ces messages, en comprenne le contenu et soit en mesure de les mettre en pratique, il est essentiel de s'appuyer sur les personnes ressources et les modes de communication locaux (dont les langues locales). Par exemple, les messages écrits en français ou en anglais sur les sachets de moustiquaires ne sont pas lus. A l'inverse, les discussions entre villageois constituent de véritables canaux d'informations et de circulation des rumeurs. Il semble important de s'appuyer sur les personnes ressources dans les *fokontany* pour diffuser les messages de prévention, et organiser avec elles des espaces d'échange où la population pourra s'informer et poser des questions – ce qui n'est pas toujours le cas lors des assemblées générales. Les leaders religieux locaux, personnalités ayant une certaine légitimité, pourraient par exemple être impliqués dans les actions de prévention. Des visites à domicile, par des personnes connues, peuvent aussi constituer un moyen efficace de diffusion des messages. De la même manière, afin de limiter les préjugés ou les critiques à l'égard des agents aspergeurs, les CAID seraient mieux acceptées si elles étaient réalisées par des agents connus de la population, des personnes de confiance.
Enfin, puisque ce sont les femmes qui ont en charge la santé des enfants, il paraît essentiel de les impliquer dans les campagnes de sensibilisation.

Renforcer le rôle des AC et des soignants de proximité

L'un des enjeux de l'amélioration de la prise en charge du paludisme à Madagascar est de lutter contre le retard aux soins. Il semble qu'à cet effet le renforcement du rôle des Agents communautaires et des soignants de proximité soit une piste à développer. Les Agents communautaires que nous avons enquêtés sont demandeurs de davantage de formations et de responsabilités dans la prise en charge du paludisme et des problèmes de santé en général. Le renforcement de leur rôle doit s'accompagner d'une reconnaissance en termes de compensation financière, afin de leur permettre de consacrer plus de temps à cette activité sans perdre les revenus générés par leur activité professionnelle[58]. Cette reconnaissance ou contrepartie financière éviterait également certaines pratiques mises en œuvre par des Agents communautaires (demande de participation financière aux villageois lors des distributions de moustiquaires à Mananjary par exemple). Dans les zones les plus isolées, la formation et la rémunération de soignants ambulants ou de proximité pourraient pallier le problème de l'éloignement des structures de santé.

Dans le cadre de ce renforcement des capacités locales, des initiatives déjà mises en places telles que « kaominina mendrika salama » (USAID et Santénet 2) pourraient constituer des pistes à approfondir.

[58] L'un des Agents communautaires enquêtés doit parfois rémunérer quelqu'un pour travailler dans ses champs pour être disponible pour les villageois (consultations, délivrance de médicaments…).

VI. ANNEXES : CANEVAS D'ENTRETIENS

Canevas d'entretien
« Habitants »

Phrase introductive / Consigne inaugurale
Présentation : étudiante / apprentie chercheure / chercheure en sociologie
On s'intéresse aux maladies fréquentes dans la région et à leurs conséquences sur la vie de tous les jours.
On leur demande s'ils sont d'accord pour répondre à quelques questions en précisant que c'est anonyme. Demander à la personne si ça ne l'ennuie pas qu'on enregistre l'entretien (si refus : prise de notes).
Introduire l'entretien en lançant la discussion en fonction du contexte (ex : présence d'enfant / activités de la personne…).

I. Introduction sur les maladies

- Pourriez-vous énumérer les maladies qui existent dans la région/ dont vous avez déjà entendu parler ?
- Quelles sont les plus graves ? Pourquoi / en quoi ?
- Quelles sont les causes de ces maladies ?
- Quel est le lien entre ces causes et le symptôme (exemple : lien entre eau et diarrhée, froid et fièvre, moustique et palu) *Faire expliquer : si la personne évoque l'eau, demander si c'est en buvant l'eau, en s'y baignant, eau froide/chaude, jour/nuit... Idem pour le vent et les autres causes citées.*
- Qu'est-ce qu'on peut faire pour se soigner (*poser la question pour chaque maladie citée)* ?
- Que se passe-t-il/qu'est-ce que vous faites quand un membre de la famille est malade ?
- Qu'est-ce que vous faites en premier ? Et ensuite ? (*Identifier dans quel ordre le recours au soin se déroulera et les différents acteurs de soin consultés)*
- Comment la décision est-elle prise du recours à l'un ou l'autre ?
- Qui prend cette décision, pourquoi ?
- Pourquoi choix de recours ou non recours aux formations sanitaires (accessibilité financière et géographique/perception de l'efficacité)? Dans quel cas allez-vous voir tel acteur de soin *(selon ceux cités précédemment / aborder les structures officielles, privées et publiques)* ? Dans quell cas vous n'y allez pas ?
- Quelles sont les spécialités de chacun des acteurs cités (*ombiasy*, docteur, matrone) / pour quel type de maladie sont-ils efficaces ?
- Quelles sont les méthodes utilisées pour ces différents acteurs (traitements proposés) / Quel type de soin / traitement donnent-ils ?
- Pour chacun : quel est le coût de la consultation/du traitement… ?
- Est-ce que c'est différent (maladie/parcours de soin/acteur consulté/traitement) pour les hommes, les femmes, les enfants, les personnes âgées, les femmes enceintes ? *Faire référence à un membre de la famille : quand c'est votre enfant /*

votre parent…, qu'est-ce que vous faites? Prendre des exemples concrets. + Avoir en tête que les femmes enceintes et les enfants jusqu'à 5 ans bénéficient d'une prise en charge par des relais communautaires, les parcours de soin sont donc différents.
- Perception des acteurs de prise en charge des soins par la personne interviewée : vous personnellement qui préférez-vous aller voir ? Pourquoi ?

II. Perceptions et représentations de la « fièvre » et du « paludisme »

- Qu'est-ce que la fièvre ?
- Quels en sont les signes/symptômes ?
- Quelles en sont les causes ? *(idée de contagion ? lien avec la fatigue ?)*
- Si le moustique est évoqué : quel moustique (tous, certains, lesquels ?) ?
- Que fait-on en cas de fièvre (relancer : automédication/recours aux soins/ qui est consulté ? A quel moment ? Pourquoi on consulte / pourquoi on ne consulte pas ?…) *Différencier à chaque fois pour les adultes/enfants, femmes/hommes/femmes enceintes/personnes âgées*
- *Si des noms de maladie sont évoqués :* qui pose le diagnostic (soi-même, docteur, autre ?) / comment sait-on que c'est ça ?
- Faire préciser si on parle de « tazo » / « tazomoka » / ou autre nom et à quoi cela correspond.

III. Traitements (en cas de fièvre liée ou pas au paludisme)

- Quel médicament prenez-vous en cas de palu ? *(paludar, palustop, ACT, etc.) Distinguer palu auto-diagnostiqué, palu diagnostiqué sur base des symptômes cliniques et diagnostic confirmé par un test (TDR, examen microscopique)*
- Si prise de paludar, palustop (et non ACT) pourquoi ? Qui vous conseille dans la prise de ces médicaments ? S'il est prescrit, par qui ?
- Si prise d'ACT : Comment se prend-il ? Dans quel cas ? Où peut-on se le procurer ? Prix ? S'il est prescrit, par qui ?
- Perception du traitement ACT : qu'est-ce qui vous plaît/déplaît dans ce traitement ? Si plusieurstypes :préférence ?
- Recours au traitement *(tous traitement cité, ancien ou nouveau)*: S'agit-il d'un geste exceptionnel ou banalisé ? Détournement d'usage des médicaments (autres maladies ?) ?
- Prises d'autres médicaments ?
- Quel traitement préférez-vous ? Pourquoi ?

IV. Prévention

1) Pratiques de prévention du paludisme :
    - Que faites-vous pour éviter d'avoir le « paludisme » *(relancer : aspirine, tisane, moustiquaire, cuisiner dans la maison pour chasser les moustiques…)* ? Demander à chaquefois : qui, comment…
      + Questions autour de l'immunité si la personne pense qu'elle n'a pas besoin de se protéger *(certaines personnes se croient immunisées, si c'est le cas, demander : pourquoi ? comment ?)*

- Questions autour de la moustiquaire :

1. A quoi elle sert ? Sous quelle forme est-elle le plus efficace ? Relancer sur :
    a. Couleur *(par exemple le blanc renvoie à la mort…)*
    b. Texture
    c. Forme
    d. imprégnée/non imprégnée (font-ils la différence ?)
2. Possession ? Où vous l'êtes vous procurée ? Quand ? Durée de l'efficacité ? Coût ?
3. Période de la vie ou de la journée où il faut le plus se protéger (enfance, grossesse…) ?
4. Utilisation : quand l'utilisez-vous (saison, période de la vie…) ? Pourquoi ? A quelle fréquence ? Comment est-elle installée *(crochet, punaise… Faire des observations et éventuellement prendre des photos)* ? Chez vous, qui décide de l'utilisation des moustiquaires ? Comment êtes-vous informé sur les modes d'utilisation des moustiquaires ? Autres modes d'utilisations (détournement) ?
5. Pour ceux qui ne l'utilisent pas : pourquoi ?
6. Toxicité des moustiquaires : quels sont les désagréments liés aux moustiquaires ?

2) Connaissances des messages officiels de prévention :
    - Y a-t-il eu des campagnes de sensibilisation sur le paludisme dans le village ?
    - Sous quelle forme, quel support (réunion d'information, prospectus, affiche…) ?
    - Quand ?
    - A votre avis, les messages étaient-ils adaptés ?
    - Qui a été impliqué dans cette sensibilisation (autorités locales, acteurs de soin, agents communautaires, villageois…) ?
    - Préférez-vous les informations transmises par écrit, par oral lors de réunion, à la maison… ?
    - A votre avis, y a-t-il des périodes plus adaptées pour la diffusion des messages de prévention (saison, jour de la semaine, période de travail/congé…) ?
    - Quels sont les informations, les recommandations que vous avez retenues ?
    - Connaissez-vous le PNLP ? Qu'est-ce qu'il représente pour vous ?

3) CAID (identifier le nom local) :
    - A quoi cela sert-il ?
    - Qui a procédé à la CAID dans vos maisons ?
    - Tous les villageois en bénéficient ?
    - Pour quelles raisons pourriez-vous refuser la CAID ?
    - Connaissez-vous des personnes qui l'ont refusée ? savez-vous pourquoi ? *Attention : question délicate à aborder avec prudence*
    - Est-ce efficace ? Pendant combien de temps ?
    - Y a t-il des désagréments ?
    - Qu'avez-vous pensé de la CAID la première fois qu'on vous en a parlé ? Si méfiance pourquoi ? *Faire décrire : comment ça s'est passé la première fois au village ? Tout le monde a-t-il accepté ? Pourquoi ? Y a-t-il des*

méfiances/peurs ? Qui a expliqué aux villageois de quoi il s'agissait ? Est-ce que les leaders traditionnels ou autre autorité villageoise ont été impliqués dans la mise en place de CAID ?

4) TPI pendant la grossesse *(à ne poser qu'aux femmes ayant eu des enfants)* :
   - Avez-vous eu des CPN pendant votre/vos grossesses ? Combien ?
   - Connaissance du TPI ? A quoi cela sert ?
   - Vous a-t-on donné des médicaments pendant la grossesse ? à quoi servaient ces médicaments ? Combien de fois ? *(Chercher à savoir s'il s'agissait du TPI et ce qu'elle en pense : est-ce que vous trouvez que c'est bien ? pourquoi ?Avez-vous accepté tout de suite ? …)*
   - Quels sont les désagréments de ce médicament ?

Volet identification
Age, nombre d'enfant, profession/occupation, niveau scolaire, origine (né au village ou ailleurs), personnes vivant dans le foyer.

Structure de santé la plus proche/temps de marche pour se rendre au centre de santé / transport utilisé

Canevas d'entretien

« Prestataires de soin »

I. Traitement

- Quel traitement contre le paludisme prescrivez-vous à vos patients ? *Préciser :*
  - *Cas de palu simple vs compliqué*
  - *Patient capable de prendre traitement oral ou non*
  - *Autres critères pouvant modifier comportement thérapeutique : noter les critères cités par le prestataire de soins*
- Que pensez-vous de ce nouveau traitement (ACT) ?
- Avez-vous déjà connu des situations de réticence des patients à la prise du nouveau traitement anti-palu (ACT) ? Si oui expliquez.
- En milieu rural, les personnes ont-elles connaissance du changement de traitement ?
- Si oui, ce nouveau médicament est-il accepté ?
- Les anciens traitements (paludar, palustop) sont-ils encore disponibles sur le marché ?
- Ces médicaments sont-ils encore utilisés par les patients ? Si oui, pourquoi ?
- Ces médicaments sont-ils encore prescrits par certains prestataires ? Si oui, pourquoi ?
- Quel traitement pensez-vous que les patients préfèrent ?

II. Délais de prise en charge

- Selon vos observations, les patients atteints de palu arrivent combien de jours/heures après déclaration des premiers symptômes ?
- Viennent-ils directement chez vous ? Si non expliquez le parcours.
- Les patients vous font-ils part de leur parcours thérapeutique avant référence à votre structure sanitaire ?
- Selon vous, le délai de prise en charge dans une formation sanitaire varie-t-elle en fonction du type de patient : homme/femme/femme enceinte/enfant/vieux ?

III. Prévention

- Avez-vous été confronté à des réticences par rapport aux moyens de prévention (CAID, MID, TPI) ? Décrivez les situations et expliquez.
- (+ se renseigner par rapport au taux de couverture des CPN et prise de TPI par les femmes enceintes dans chaque zone), en quoi est dû ce résultat ?
- Comment expliquez-vous que le taux de couverture des CPN est autour de 80% tandis que la prise de TPI est bien plus faible (26%) ?
- Selon vous, d'où vient le blocage : patient ? Prestataire de soin ? Pourquoi ?

IV. Sensibilisation

- Pensez-vous que c'est votre rôle de participer à la sensibilisation des patients (durant les consultations) ?
  - Si oui le faites vous ?
    - quels sont les messages que vous leur communiquez ?
    - quels sont les moyens dont vous disposez pour sensibiliser vos patients (supports, etc.) ?
    - Depuis quand faites-vous de la sensibilisation ?
    - Quels résultats (comportements) avez-vous constaté chez les gens après avoir été sensibilisés ?
    - D'après votre expérience à quoi ces comportements peuvent-ils êtres dus ?
  - Si non, pourquoi ?

- Quels sont les ONG, associations et autres prestataires de soins qui font de la sensibilisation pour la lutte contre le paludisme dans le village ? S'il en existe, qui sont-ils, quels sont leurs rôles ? Depuis quand interviennent-ils ? Comment collaborent-ils avec vous ?

V. Difficultés

Quelles difficultés rencontrez-vous dans la prise en charge du palu ? (manque de moyens, rupture de stock de traitements et de TDR, populations pas suffisamment sensibilisées, rapports difficiles avec le PNLP, etc.)
